# Non-viral Induction of Transgene-free iPSCs from Somatic Fibroblasts of Multiple Mammalian Species

**DOI:** 10.1016/j.stemcr.2021.03.002

**Published:** 2021-04-01

**Authors:** Sho Yoshimatsu, Mayutaka Nakajima, Aozora Iguchi, Tsukasa Sanosaka, Tsukika Sato, Mari Nakamura, Ryusuke Nakajima, Eri Arai, Mitsuru Ishikawa, Kent Imaizumi, Hirotaka Watanabe, Junko Okahara, Toshiaki Noce, Yuta Takeda, Erika Sasaki, Rüdiger Behr, Kazuya Edamura, Seiji Shiozawa, Hideyuki Okano

**Affiliations:** 1 Department of Physiology, School of Medicine, Keio University, 35 Shinanomachi, Shinjuku-ku, Tokyo, Japan; 2 Laboratory for Proteolytic Neuroscience, RIKEN Center for Brain Science, Saitama, Japan; 3 Laboratory for Marmoset Neural Architecture, RIKEN Center for Brain Science, Saitama, Japan; 4 Laboratory of Veterinary Surgery, Department of Veterinary Medicine, College of Bioresource Sciences, Nihon University, Kanagawa, Japan; 5 Department of Pathology, School of Medicine, Keio University, Tokyo, Japan; 6 Department of Marmoset Biology and Medicine, Central Institute for Experimental Animals, Kanagawa, Japan; 7 Research Platform Degenerative Diseases, German Primate Center – Leibniz Institute for Primate Research, Göttingen, Germany; 8 DZHK (German Centre for Cardiovascular Research), partner site Göttingen, Göttingen, Germany

**Keywords:** induced pluripotent stem cell, reprogramming, marmoset, dog, pig, neural stem cell, primordial germ cell, mammalian, episomal vector

## Abstract

Induced pluripotent stem cells (iPSCs) are capable of providing an unlimited source of cells from all three germ layers and germ cells. The derivation and usage of iPSCs from various animal models may facilitate stem cell-based therapy, gene-modified animal production, and evolutionary studies assessing interspecies differences. However, there is a lack of species-wide methods for deriving iPSCs, in particular by means of non-viral and non-transgene-integrating (NTI) approaches. Here, we demonstrate the iPSC derivation from somatic fibroblasts of multiple mammalian species from three different taxonomic orders, including the common marmoset (*Callithrix jacchus*) in *Primates*, the dog (*Canis lupus familiaris*) in *Carnivora*, and the pig (*Sus scrofa*) in *Cetartiodactyla,* by combinatorial usage of chemical compounds and NTI episomal vectors. Interestingly, the fibroblasts temporarily acquired a neural stem cell-like state during the reprogramming. Collectively, our method, robustly applicable to various species, holds a great potential for facilitating stem cell-based research using various animals in *Mammalia*.

## Highlights

• A species-wide method for deriving transgene-free iPSCs has been established
• Primary colony-forming cells showed a neural stem cell-like profile, named iNSLCs
• mRNA-seq was performed for dissecting the difference between iNSLCs and iPSCs
• Single-cell analysis was performed for assessing the heterogeneity of iNSLCs

## Graphical abstract

**Figure:**
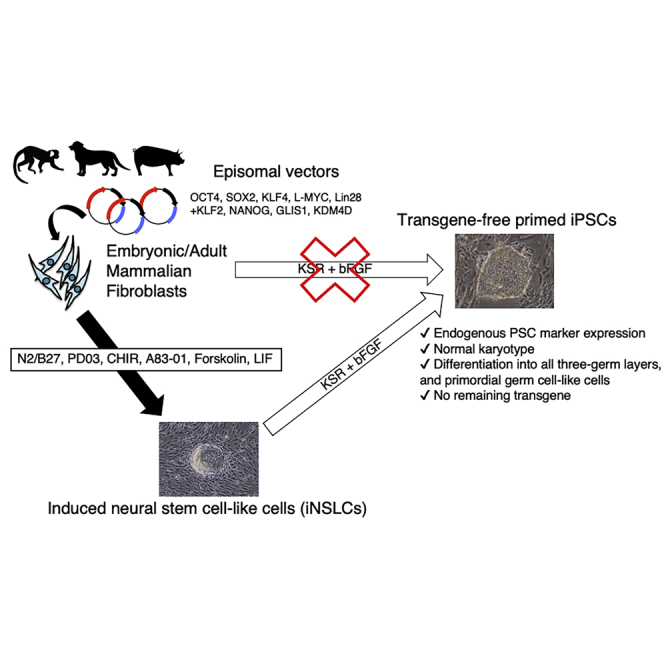

## Teaser

In this article, Okano and colleagues established a species-wide method for deriving transgene-free iPSCs, and discovered primary colony-forming cells showed a neural stem cell-like profile, named induced neural stem cell-like cells (iNSLCs).

## Introduction

Embryonic stem cells (ESCs), derived from the inner cell mass of pre-implantation blastomeres, have potentials for unlimited proliferation by self-renewal and for differentiation into all three germ layers and germ cells (Smith, 2001). As such, ESCs have been considered to be in a “pluripotent” state, referred as pluripotent stem cells (PSCs). The first demonstration of ESC derivation was performed with mice (Evans and Kaufman, 1981; Martin, 1981), and subsequently with non-human primates (NHPs) (Thomson et al., 1995) and humans (Thomson et al., 1998). However, ethical concerns and resource limitations have been imposed on the usage of early blastomeres from several mammalian species, including NHPs and humans. Moreover, the maintenance of *in vitro* culture of early-stage embryos remains challenging, especially for many wildlife mammalian species (Cordova et al., 2017). These circumstances emphasize the necessity of other species-wide approaches for obtaining PSCs.

Alternatively, an unlimited source of cells can be derived from induced PSCs (iPSCs) without ethical and practical limitations. Reprogramming of somatic fibroblasts into iPSCs has been demonstrated in mice (Takahashi and Yamanaka, 2006) and in humans (Takahashi et al., 2007) by the ectopic overexpression of defined factors, such as *OCT4*, *SOX2*, *KLF4*, and *C-MYC*. The resultant iPSCs have a wide range of applicability for disease modeling *in vitro* and for regenerative medicine (Fujimori et al., 2018; Okano and Yamanaka, 2014; Yamanaka, 2012). In addition, developmental studies have proven that iPSCs have a potential for giving rise to new offspring, similarly to ESCs (Bradley et al., 1984). This has been verified in studies with rodents and pigs, in which germline-transmitting chimera formation was achieved through blastocyst injection (Hamanaka et al., 2011; Honda et al., 2017; Okita et al., 2007; Thomas and Capecchi, 1987; Wernig et al., 2007; West et al., 2011) or by directly inducing functional mature gametes (Hayashi et al., 2011, 2012). For over 10 years, the reprogramming technology has been performed and validated in a variety of mammalian species, including great apes (Marchetto et al., 2013), farm animals (Ogorevc et al., 2016), and endangered species (Ben-Nun et al., 2011; Honda et al., 2017). Moreover, iPSCs derived from various species have paved the way for evolutionary studies assessing species differences *in vitro* (Marchetto et al., 2013).

Although the definition of bona fide iPSCs remains elusive, it is well-known that fully reprogrammed iPSCs sustain a pluripotent state in the absence of transgene expression (Okita et al., 2007). At the dawn of iPSC reprogramming, transgene-integrating retroviruses were used for deriving iPSCs (Takahashi et al., 2007; Takahashi and Yamanaka, 2006; Wernig et al., 2007; Yu et al., 2007) and the transgenes were gradually silenced after the iPSCs were fully reprogrammed. However, viral transduction raised concerns for the clinical application of iPSCs. Yu et al. (2009) were the first to report the derivation of iPSCs using non-viral, non-transgene-integrating (NTI) episomal vectors, enabling the generation of transgene-free iPSCs. Since residual transgenes in the iPSCs restrict their utility for *in vivo* and *in vitro* differentiation (Nair, 2008; Okita et al., 2007) and for the generation of iPSC-derived offspring (Du et al., 2015; Maherali et al., 2007; Okita et al., 2007; Wernig et al., 2008), NTI approaches for iPSC derivation have been intensively studied in recent years. Although complete transgene excision from the iPSCs has been demonstrated in rodents (Li et al., 2017; Wu et al., 2014) and in humans (Okita et al., 2011, 2013; Yu et al., 2009), it still remains a challenge in other species.

In this study, we demonstrated the derivation of transgene-free iPSCs from somatic fibroblasts of multiple mammalian species from three different taxonomic orders, including the common marmoset (marmoset; *Callithrix jacchus*) in *Primates*, the dog (*Canis lupus familiaris*) in *Carnivora*, and the pig (*Sus scrofa*) in *Cetartiodactyla*, by combinatorial usage of small molecules and NTI episomal vectors. We also demonstrated the differentiation potential of these iPSCs into all three germ layers and primordial germ cell-like cells (PGCLCs). Interestingly, during the reprogramming process, we observed that the primary colony-forming cells showed neural stem cell (NSC)-like characteristics, which could be sustained over time when the cells were cultured in the same medium used for induction of these cells. Our data suggest that the reprogramming method would be invaluable for deriving transgene-free iPSCs from somatic fibroblasts of various mammalian species.

## Results

### Derivation of Primary Colonies from Marmoset Fibroblasts Using Episomal Vectors

**Figure 1:**
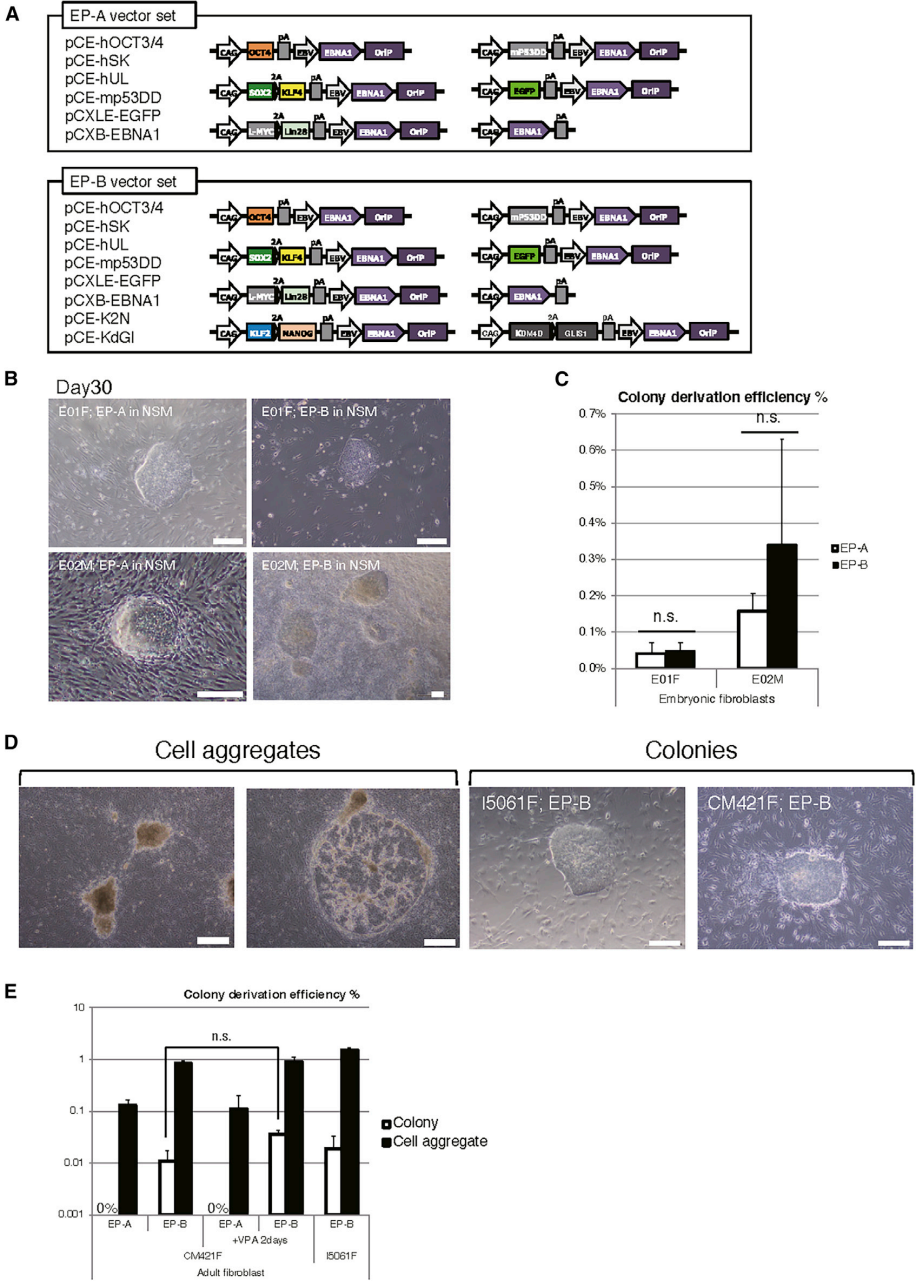
Derivation of Primary Colonies from Marmoset Fibroblasts Using Episomal Vectors (A) Schematic of plasmids used for vector transfection. (B) Representative images of primary colonies derived from E01F and E02M fibroblasts using NSM at day 30. Scale bars, 200 μm. (C) Derivation efficiency of primary colonies from E01F and E02M fibroblasts using NSM (n = 3, independent experiments). (D) Representative images of cell aggregates (left) and primary colonies (right) derived from I5061F and CM421F fibroblasts. Scale bars, 500 μm (left), 200 μm (right). (E) Derivation efficiency of primary colonies (white box) and cell aggregates (black box) from I5061F and CM421F fibroblasts (n = 3, independent experiments). n.s., not significant.

First, we attempted to assess the derivation efficiency of primary colonies from dorsal skin-derived fibroblasts of embryonic marmosets (named E01F and E02M) using a set of Epstein-Barr virus *EBNA1-* and *OriP*-based episomal vectors (Okita et al., 2013) encoding five reprogramming factors (human *OCT4*, *SOX2*, *KLF4*, *L-MYC*, and *LIN28*), a dominant-negative mutant of mouse *Trp53* (*mp53DD*), and enhanced green fluorescent protein (EGFP) for assessing the transfection efficiency (collectively named the “EP-A vector set,” Figure 1A, top), which is a conventional set of vectors used for deriving human iPSCs (hiPSCs) (Okita et al., 2013). These vectors were delivered into the fibroblasts by electroporation, and the transfection efficiency was calculated to be between 7% and 35% according to the EGFP fluorescence (Figure S1A). After transfection, the fibroblasts were expanded for 3–7 days in a fibroblast medium (M10), and then transferred onto mouse embryonic feeder cells in an induction medium.

However, when using a basic-fibroblast growth factor and knockout serum replacement-based ESC medium (ESM) as the induction medium (Nii et al., 2014; Yoshimatsu et al., 2019a), no colonies appeared from the E01F and E02M fibroblasts 30 days after transfection (n = 3; Figure S1B). This suggested that the conventional method for generating hiPSCs (Okita et al., 2011) was not applicable to marmoset cells. Next, we decided to utilize a medium we previously reported for inducing and maintaining putative naive-state hiPSCs and marmoset ESCs from conventional primed-state cells (termed as NSM) (Kisa et al., 2017; Shiozawa et al., 2020). Using NSM as the induction medium, we observed primary dome-shaped colonies from both E01F and E02M embryonic fibroblasts 30 days after transfection (Figure 1B, left). We then either mechanically isolated the primary colonies for clonal expansion or expanded them in bulk in NSM for further analyses. The colony derivation efficiency from EGFP-positive fibroblasts using the EP-A vector set was calculated to be between 0.040% for the E01F fibroblasts and 0.157% for the E02M fibroblasts (Figure 1C).

We also attempted to derive colonies from ear skin-derived fibroblasts of an adult marmoset (named CM421F). However, only cell aggregates were obtained from the transfected fibroblasts when using EP-A (Figure 1D, left). We speculated that the failure was due to low reprogramming efficiency and, therefore, we tested for additional factors to enhance the efficiency.

Since we previously demonstrated that the combinatorial usage of NSM and the overexpression of six factors (*OCT4*, *SOX2*, *KLF4*, *C-MYC*, *KLF2*, and *NANOG*) can convert the primed state of hiPSCs and marmoset ESCs into a naive-like state (Kisa et al., 2017; Shiozawa et al., 2020), we added an episomal vector, pCE-K2N, which harbors two of these factors (*KLF2* and *NANOG*), to the EP-A vector set. Although we were successful in obtaining primary colonies using NSM with the updated vector set, the colonies were unable to reach confluency after they were passaged or mechanically picked up.

Therefore, we further tested for two additional factors: *GLIS1* for enhancing the reprogramming efficiency (Maekawa et al., 2011), and *KDM4D* for facilitating epigenetic reprogramming (Liu et al., 2018; Matoba et al., 2014). These two factors were introduced into an episomal vector, pCE-KdGl. The new vector set with both pCE-K2N and pCE-KdGl added to the EP-A vector set was named EP-B (Figure 1A, bottom). Using EP-B, we succeeded in deriving primary colonies from ear skin-derived fibroblasts of two adult marmosets (CM421F and I5061F) 30 days after transfection (Figure 1D, right). However, unlike the culture derived from embryonic fibroblasts (Figure 1B), cell aggregates were the major population in the culture derived from adult fibroblasts (Figure 1E). In addition, we evaluated the effect of the supplementation of valproic acid (VPA), a histone deacetylase inhibitor, to the induction medium for 2 days, which was previously used for the derivation of marmoset iPSCs (Debowski et al., 2015). In our culture method, the derivation efficiency of primary colonies was not significantly enhanced with VPA supplementation (Figure 1E).

When using EP-B, we also succeeded in deriving primary colonies from embryonic fibroblasts cultured in NSM (Figure 1B, right), but not in ESM (0%, n = 3). Unlike adult fibroblasts, the colony derivation efficiencies from embryonic fibroblasts were not significantly different with EP-A or EP-B (Figure 1C).

Although the primary colony-forming cells derived from marmoset fibroblasts showed ubiquitous expression of SOX2 (Figure S1C, top) and alkaline phosphatase (AP) (Figure S1D), these cells were negative for PSC markers, such as TRA-1-60 and SSEA4 (Figure S1E). Alternatively, we found that these cells expressed markers of NSCs, such as MSI1 (Sakakibara et al., 1996) and PAX6 (Figures 4 and S6). For that reason, we tentatively named the primary colony-forming cells cultured in NSM as putative iNSLCs (induced NSC-like cells).

### Derivation of Marmoset Transgene-free iPSCs

**Figure 2:**
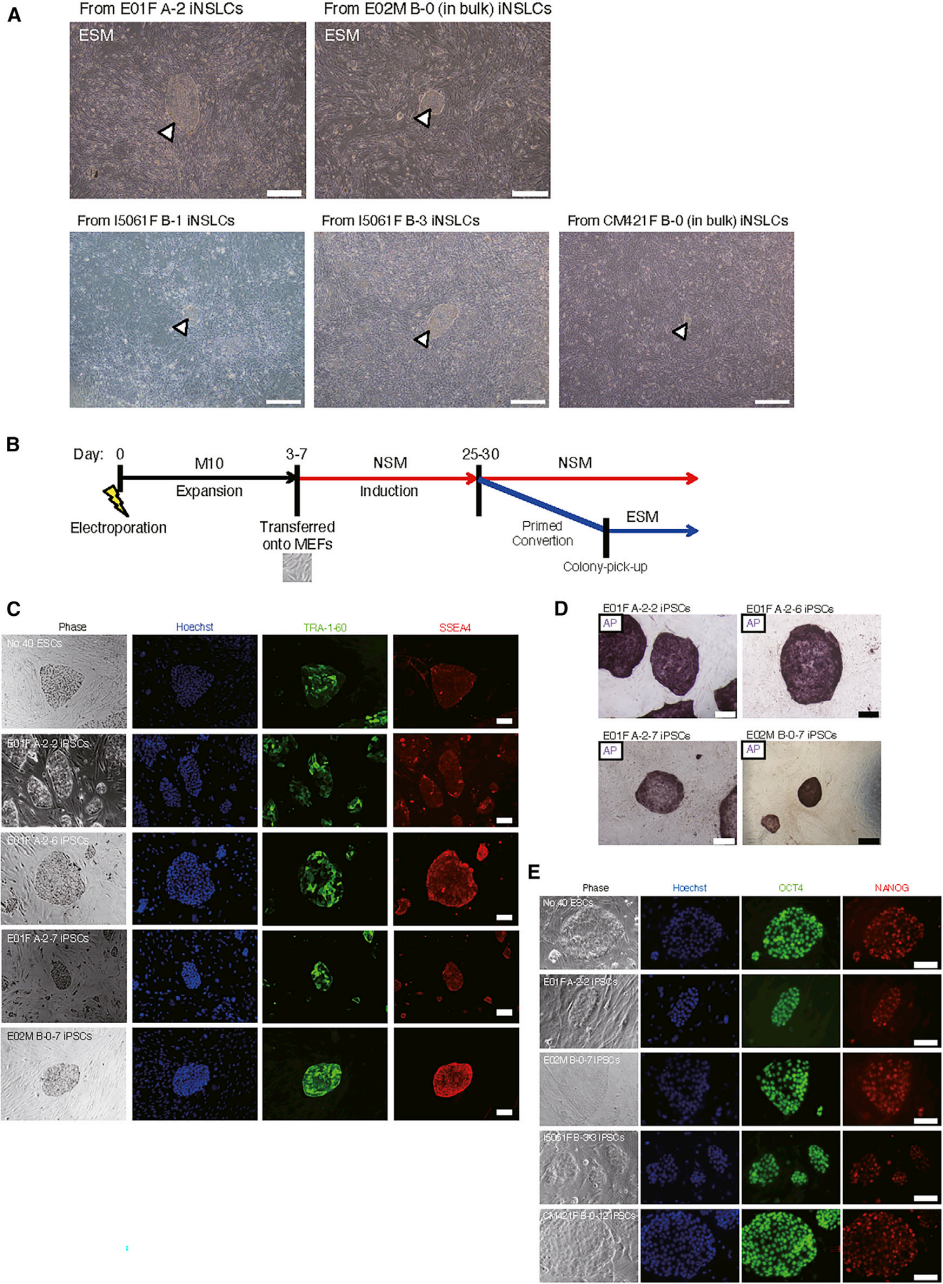
Derivation of Marmoset Transgene-free iPSCs (A) Representative images of putative iPSC colonies. Following primed conversion of iNSLCs, ESC-like (iPSC) colonies appeared (white arrowhead). (B) Timetable for the derivation of marmoset iPSCs. Day 0 was defined as the timing of vector transfection. (C) Immunocytochemical staining of iPSCs using TRA-1-60 and SSEA4 antibodies. No. 40 ESCs were used as positive controls (top). Scale bars, 100 μm. See Figure S2B for iPSC clones derived from adult marmosets. (D) AP staining of iPSC colonies. Scale bars, 500 μm. See Figure S2A for iPSC clones derived from adult marmosets. (E) Immunocytochemical staining of iPSCs using OCT4 and NANOG antibodies. Scale bars, 100 μm.

Since the iNSLCs were TRA-1-60 and SSEA4 negative, we next attempted to investigate their potential to convert into PSCs by culturing these cells in ESM, which is routinely used for culturing marmoset ESCs. Surprisingly, ESC-like colonies emerged after culturing the iNSLCs in ESM for 3 weeks (Figure 2A). On average, from sub-confluent iNSLCs at early passages (P1–2) in one well of a 6-well plate (∼1 × 10^6^ cells), ∼50 ESC-like colonies appeared. This step was termed as "primed conversion" (Figure 2B). In contrast, iNSLCs at late passages (P4–6) were not competent for primed conversion (from over 6 × 10^6^ cells, n = 3). The ESC-like colonies were then mechanically isolated, followed by clonal expansion in ESM for further analyses. Since the morphology of the ESC-like cells cultured in ESM were indistinguishable from that of conventional marmoset ESCs (Sasaki et al., 2005), showing a tightly packed colony structure with defined borders and high nuclear/cytoplasm rate, we termed these cells as putative marmoset iPSCs.

Immunocytochemical analysis of the putative iPSCs resulted in a similar staining pattern of TRA-1-60 and SSEA4 to that of the no. 40 marmoset ESC clone (Sasaki et al., 2005) (Figures 2C and S2B). They were also positive for AP (Figures 2D and S2A), OCT4, and NANOG (Figure 2E). On the other hand, they were not positive for other PSC markers, including SSEA1 and SSEA3, as shown previously (Yoshimatsu et al., 2020), or for NSC markers, including MSI1 and PAX6 (data not shown). To confirm whether the putative iPSCs expressed PSC marker genes endogenously, we performed quantitative reverse-transcriptiase PCR (qRT-PCR) using specific primers for endogenous mRNA sequences of marmoset *OCT4*, *NANOG*, *SOX2*, *KLF4*, *ZFP42* (*REX1*), *LIN28* (*LIN2*8A), *DPPA5*, and *TERT* genes. While fibroblasts only expressed *KLF4*, the putative iPSCs expressed all of the analyzed PSC marker genes endogenously (Figures S2C−S2H).

**Figure 3:**
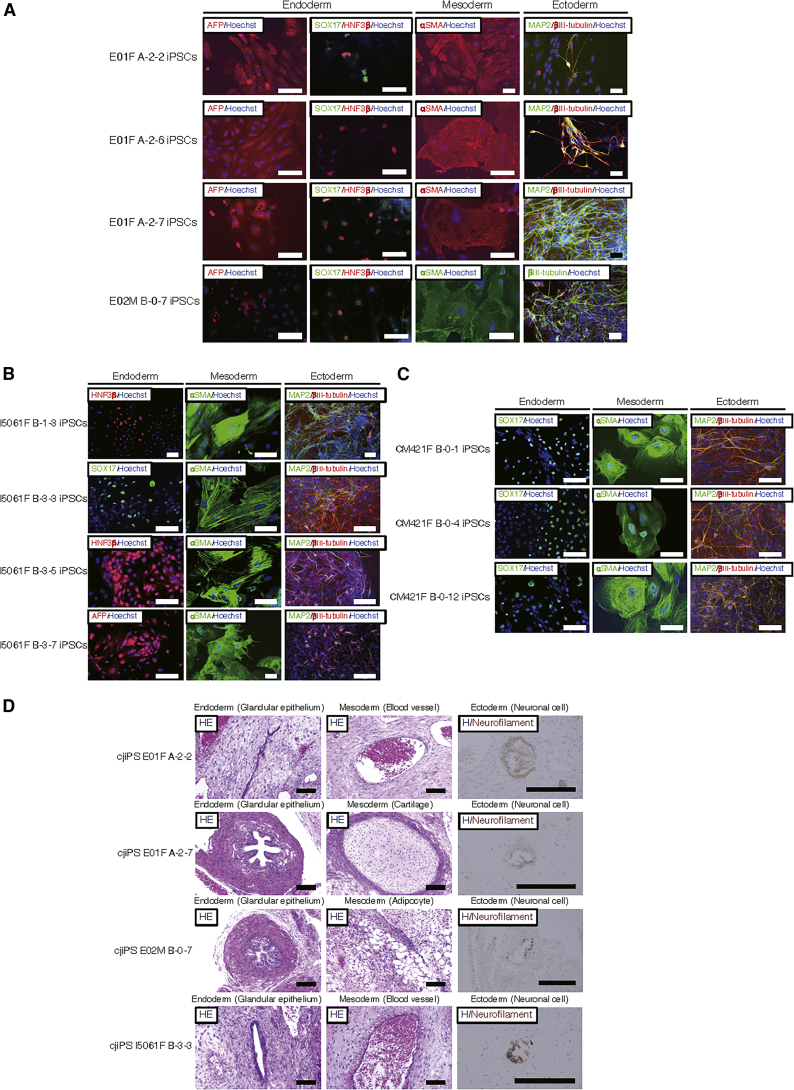
Three-Germ Layer Differentiation of Marmoset iPSCs (A–C) Representative images of endodermal (AFP, HNF3β, SOX17-positive), mesodermal (αSMA-positive), and ectodermal (MAP2, βIII tubulin-positive) cells differentiated from marmoset iPSCs by *in vitro* differentiation assay. Scale bars, 100 μm. (D) Representative images of three-germ layer tissues or cells in teratomas derived from marmoset iPSCs. H&E, hematoxylin and eosin staining; H/Neurofilament, hematoxylin staining with immunocytochemical staining using anti-neurofilament 200 kDa antibody. Scale bars, 100 μm.

At 9–14 passages after the derivation of the putative iPSCs, most clones (5/6 of E01F, 1/2 of E02M, 9/9 of I5061F and, 4/4 of CM421F) showed successful removal of all the episomal vectors, which was confirmed by genomic PCR (Figures S3A−S3D) using highly specific and sensitive primers for the OriP sequence (Yu et al., 2009) (Figure S3E). We also designed and validated specific primers for each episomal vector (Figures S3F and S3G), and confirmed their removal in four representative iPSC clones, including those from two embryonic marmosets (E01F A-2-2 and E02M B-0-7) and two adult marmosets (I5061F B-3-3 and CM421F B-0-12) (Figure S3H), while the parental, primed conversion-competent iNSLCs at an early passage (P1) harbored all episomal vectors (Figure S3I). We performed chromosome counting by G-banding of six representative iPSC lines (Figure S4A), and found 46% of CM421F B-0-4, 88% of I5061F B-3-3, 88% of E02M B-0-7, 84% of E01F A-2-7, 58% of E01F A-2-6, and 76% of E01F A-2-2 retained normal chromosome number (2n = 46), and these euploid cells showed normal karyotype, analyzed by Q-banding (Figure S4B). Furthermore, we confirmed *in vitro* and *in vivo* three-germ layer differentiation potentials of the putative iPSC lines (Figures 3A−3D).

In addition, we explored the differentiation potential of marmoset iPSCs into germ cell linage by the combinatorial usage of cytokines (Hayashi et al., 2011) and transcription factors (Kobayashi et al., 2017) for *BLIMP1-Venus* knockin E01F A-2-2 iPSCs (Figures S5A and S5B). By using an optimized PGCLC induction method for marmoset ESCs (Figures S5C and S5D; Note S1), the iPSCs were differentiated into Venus-positive PGCLCs (Figures S5E–S5I; Note S1; Table S1), whose transcriptome was comparable to those of PGCLCs derived from reporter knockin marmoset ESCs (Yoshimatsu et al., 2019b, 2020).

In sum, the putative iPSCs retained normal karyotypes, and acquired and maintained pluripotency, which was independent of transgene expression. Furthermore, they possessed the capacity to differentiate into tissues of all three germ layers and PGCLCs. Thus, these putative iPSCs will be referred to as transgene-free iPSCs for the subsequent results described below.

### Characterization of the Marmoset iNSLCs

**Figure 4:**
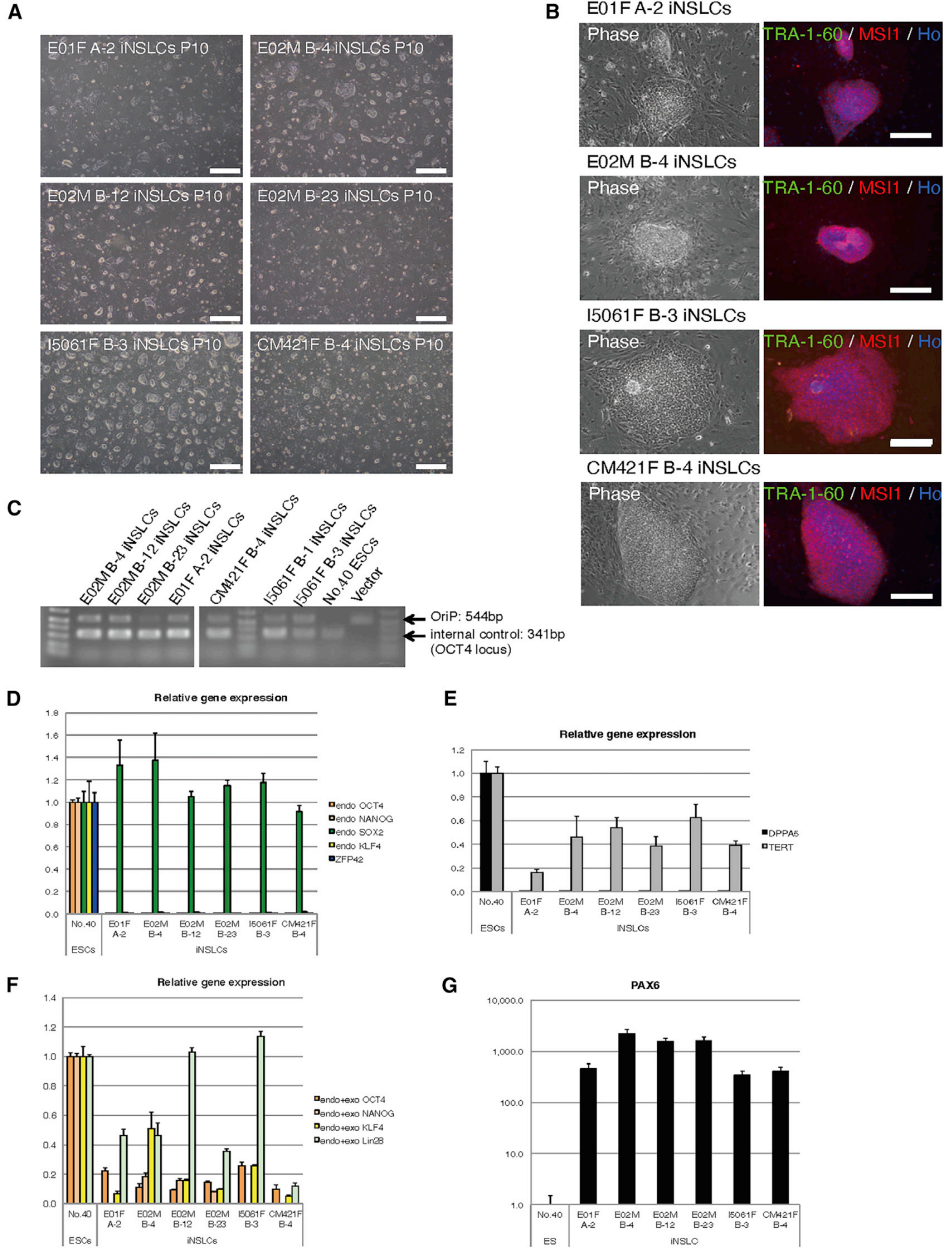
Characterization of the Marmoset iNSLCs (A) Representative images of iNSLC clones cultured in NSM following ten passages (P10). Scale bars, 500 μm. (B) Immunocytochemical staining of the iNSLCs using TRA-1-60 and MSI1 antibody. Ho, Hoechst (nuclear DNA). (C) Genomic PCR analysis for residual episomal vectors. Episomal vectors remained in all of the derived iNSLC clones at P10. (D–G) qPCR analysis of PSC/NSC markers in the iNSLCs. RNA extracted from iNSLCs at passages 4–6 was used (n = 3, independent experiments).

During our reprogramming procedure (Figure 2B), we initially obtained primary colonies considered to be putative iNSLCs, which stained positively for NSC markers, such as SOX2, PAX6, and MSI1, but negatively for TRA-1-60 and SSEA4. We used six marmoset iNSLC clones (E01F A-2, E02M B-4, E02M B-12, E02M B-23, I5061F B-3, and CM421F B-4) for further analyses to confirm that the iNSLCs represented an NSC-like state distinct from the transgene-free iPSCs. Although we showed that the iNSLCs were continuously expandable even after ten passages (Figure 4A) with sustained ubiquitous MSI1 expression (Figure 4B), episomal vectors persisted to remain in the cells (Figure 4C). By qPCR, we revealed that PSC marker genes (*OCT4, NANOG, KLF4, ZFP42*, and *DPPA5*) were not endogenously expressed in the iNSLCs (at passages 4–6), except for *SOX2* and *TERT* (Figures 4D and 4E). Meanwhile, exogenous expression of *OCT4, NANOG, KLF4*, and *LIN28* from the episomal vectors was confirmed (Figure 4F).

Next, we assessed the expression of cell lineage marker genes for elucidating the biological and developmental characteristics of the iNSLCs. By qPCR, we found that the iNSLCs strongly expressed *PAX6* (Figure 4G), whose expression is required for the self-renewal and neurogenesis of NSCs (Sansom et al., 2009). The early ectodermal markers *ZFP521* (Kamiya et al., 2011) and *SOX1* were also expressed in the iNSLCs (Figure S6A). On the other hand, we detected no or very low expression of early mesodermal or endodermal markers, such as *T* and *SOX17*, in these cells, which was even lower than that of the ESCs (Figure S6B).

We also analyzed gene expression of the primed conversion-competent iNSLCs at early passages (P1–2) (Figure 2B). The bulk iNSLCs derived from I5061F fibroblasts using EP-B (I5061F B-0 iNSLCs) showed strong endogenous expression of *SOX2* and *PAX6* (Figures S6C and S6D), while there was only exogenous expression of PSC markers, such as *OCT4* (data not shown). In addition, immunocytochemical analysis revealed that that ∼10% doublecortin-positive putative neuroblasts were present in the iNSLC colonies (Figure S6E). Meanwhile, PAX6 and SOX2 were ubiquitously expressed in the iNSLCs (Figure S6F). Furthermore, we confirmed the high neurogenic potential of the iNSLCs by direct differentiation assays (Note S2; Figures S6H–S6L) (Yoshimatsu et al., 2019a).

### Global Gene Expression Profiling of the Marmoset iPSCs and iNSLCs

**Figure 5:**
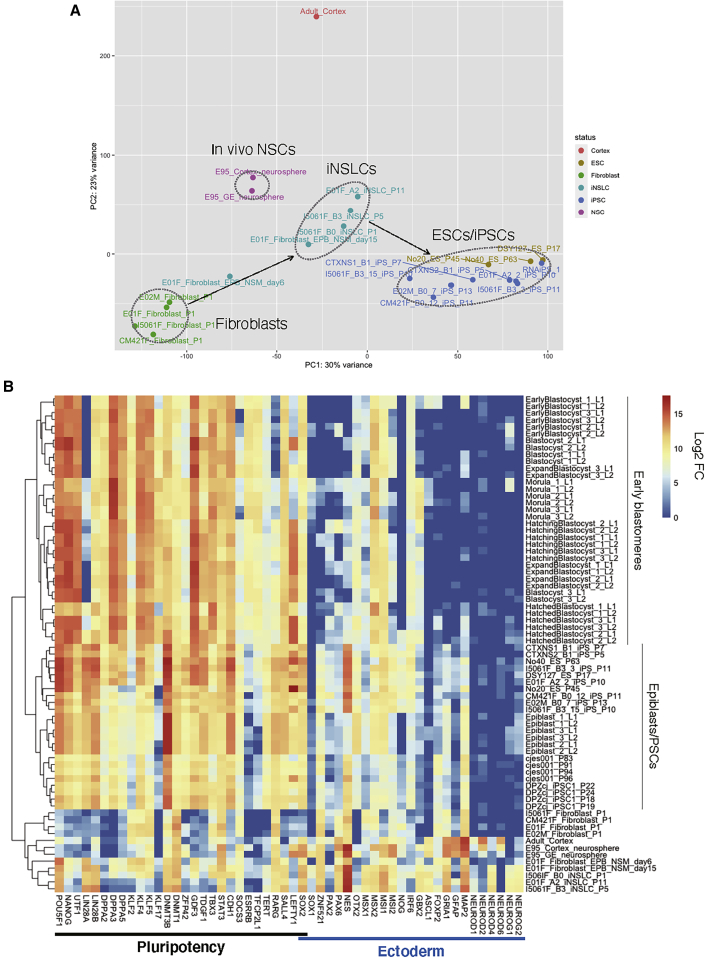
Bulk mRNA-Seq Analysis of Marmoset Cells (A) PCA of marmoset samples, including fibroblasts (FB; E01F, E02M, I5061F, and CM421F fibroblasts), EP-B-transfected fibroblasts cultured in NSM for induction (induced; E01F fibroblast EPB NSM days 6 and 15), iNSLCs (E01F A-2, I5061F B-3, and I5061F B-0 iNSLCs), ESCs (no. 40 ESCs, DSY127 ESCs, and no. 20 ESCs), iPSCs (E01F A-2-2, E02M B-0-7, I5061F B-3-3, I5061F B-3-15, CM421F B-0-12, CTXNS1 B-1, and CTXNS2 B-1 iPSCs), and *in-vivo*-derived neurospheres (in_vivo_NSC; E95 cortex neurosphere and GE [ganglionic eminence] neurosphere). Black arrows show the pseudo trajectories of the reprogramming (fibroblasts to iNSLCs, and iNSLCs to iPSCs). (B) Hierarchical clustering analysis of marmoset samples based on the expression of pluripotency and ectoderm-related markers. We included data of marmoset ESCs (cjes001) and iPSCs (DPZcj_iPSC1), early-stage embryos (morula, early blastocyst, blastocyst, expand blastocyst, hatching blastocyst, hatched blastocyst, and epiblast_1–3_L1−2), and adult marmoset cortex (Adult_Cortex) were described previously (Debowski et al., 2015; Shiozawa et al., 2020; Yoshimatsu et al., 2019a).

In this study, we initially derived iNSLCs from marmoset fibroblasts, after which they were converted into iPSCs. To elucidate the global differences and similarities of gene expression among these cells, we performed transcriptomic analyses by 3′IVT microarray (Note S3) and mRNA sequencing (mRNA-seq) (Figures 5A and 5B).

By bulk mRNA-seq analysis of marmoset samples, including fibroblasts (E01F, E02M, I5061F, and CM421F fibroblasts), EP-B-transfected fibroblasts cultured in NSM for induction (induced; E01F fibroblast EPB NSM days 6 and 15), fibroblast-derived iNSLCs, ESCs, fibroblast-derived iPSCs, *in-vivo*-derived neurospheres (E95 cortex neurosphere and GE [ganglionic eminence] neurosphere), and neurosphere-derived iPSCs (CTXNS1 B-1 and CTXNS2 B-1; see the next section for details). In addition, we included previously deposited data of marmoset ESCs (cjes001) and iPSCs (DPZcj_iPSC1) (Debowski et al., 2015), morulae and peri-implantation epiblasts (Shiozawa et al., 2020), and adult marmoset cortex (Yoshimatsu et al., 2019a). Consistent with the result of the microarray analysis (Figure S7A), Principal-component analysis (PCA) of all the analyzed gene expression clearly divided PSCs, fibroblasts, early-stage embryos, and iNSLCs (Figure 5A). In addition, we performed hierarchical clustering based on the expression of major pluripotency/ectoderm-related genes (Figure 5B). We found marmoset ESCs, iPSCs, and epiblasts were clustered together, while they were segregated from early blastomeres (morulae and blastocysts), fibroblasts, *in-vivo*-derived neurospheres, and iNSLCs (Figure 5B). By differential expressed gene (DEG) analysis of marmoset iPSC and iNSLC samples, we discovered the expression of pluripotency-related genes, including *DPPA2*, *TDGF1*, *UTF1*, *ZFP42*, *EPCAM*, and *NANOG*, was significantly higher in iPSCs, while the expression of neurogenesis-related genes, including *ASCL1*, *PAX6*, *NEUROD1*, and *NEUROG1-2*, were significantly higher in iNSLCs (Figure S7A; Table S2).

To explore the heterogeneity of the early-passage iNSLCs that were competent for primed conversion, we performed single-cell random displacement amplification sequencing (RamDA-seq) analysis (Hayashi et al., 2018). As shown in Figure S7B, we discovered that primed conversion-competent E01F A-2 iNSLCs at an early passage (P1) were clearly segregated from no. 40 ESCs and E01F A-2-2 iPSCs by PCA (iNSLCs: n = 65; ESCs/iPSCs: n = 5). However, based on the respective DEGs, we found each iNSLC showed a diverse gene expression profile, such as neurogenesis-related genes, were not highly expressed in all iNSLCs (Figure S7C), and a small population of iNSLCs showed the expression of *EPCAM*, *ZFP42*, and *DPPA2* (Figure S7D), which were estimated to be specifically expressed in iPSCs by the bulk mRNA-seq analysis (Figures 5B and S7A).

Taken together, the combination of qPCR, immunocytochemistry, and transcriptomic analyses confirmed that the iNSLCs and the transgene-free iPSCs are in two distinct cellular states. Thus, we have developed a unique reprogramming protocol for deriving transgene-free iPSCs from marmoset fibroblasts through an NSC-like state, although single-cell analysis showed that a small population of iNSLCs may already have acquired a pluripotency-directed propensity.

### Reprogramming of *In-Vivo*-Derived Neural Stem Cells toward a Pluripotent State Using ESM

We demonstrated that the marmoset iNSLCs possessed a unique property to be easily re-reprogrammed toward pluripotency, which can be explained by the expression of the residual transgenes in these cells and the fact that they are transcriptionally similar to *in-vivo*-derived NSCs, which were reported to have a higher potential to be reprogrammed into iPSCs compared with fibroblasts in mouse and human cells (Note S4). This motivated us to assess the reprogramming capacity of *in-vivo*-derived NSCs by using only ESM. As a result, we demonstrated iPSC generation from primary NSCs, which were derived from the biopsy of cerebral cortexes from two embryonic marmosets (Note S4).

### Derivation of Transgene-free iPSCs from Canine Fibroblasts

Since we succeeded in the derivation of iNSLCs from human fibroblasts by the same method (Note S5). We sought to test the reprogramming method for fibroblasts of other non-rodent/non-primate mammalian species. Therefore, we attempted to reprogram ear skin-derived fibroblasts obtained from an adult dog (named K9) into iPSCs.

**Figure 6:**
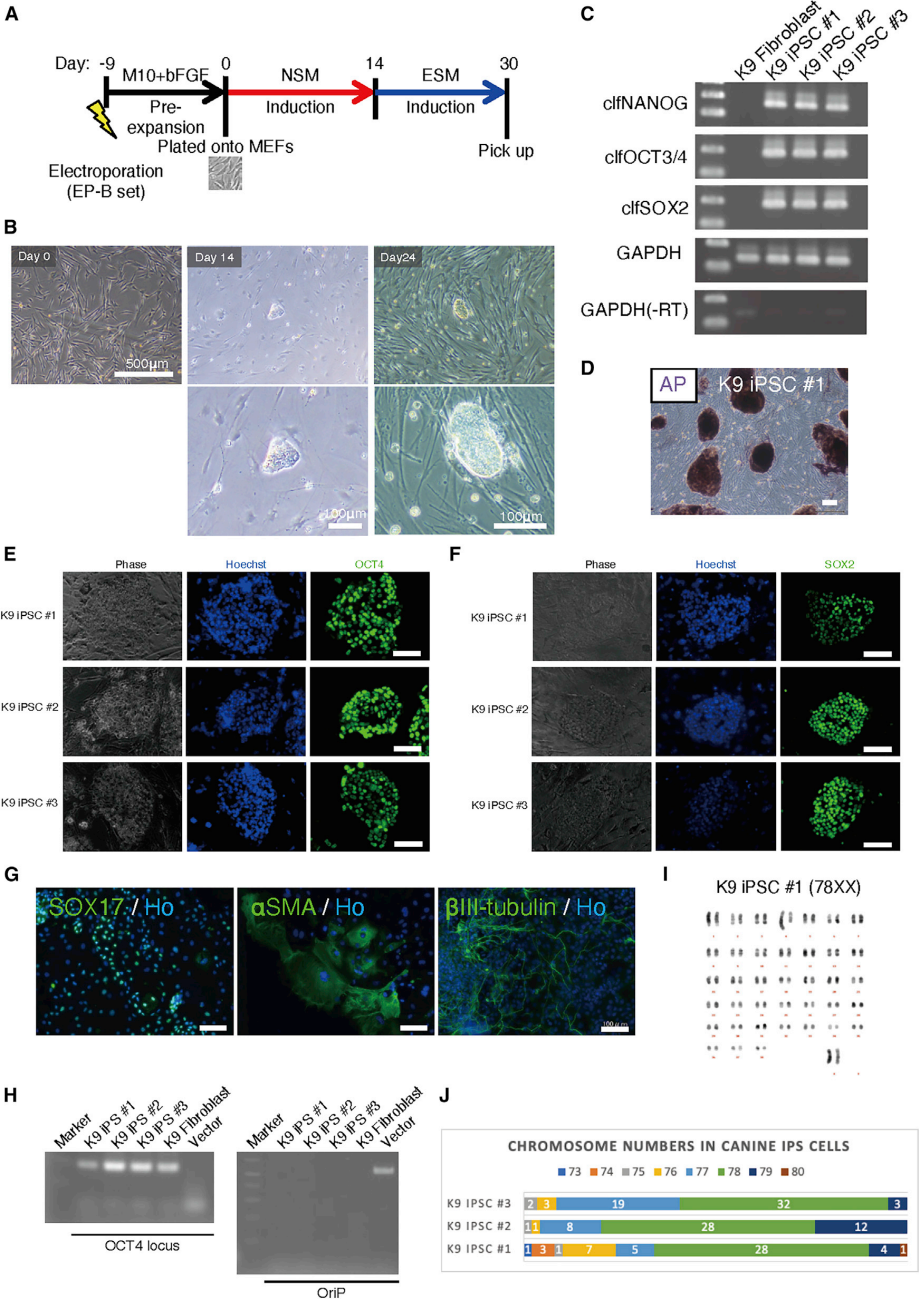
Derivation of Transgene-free iPSCs from Canine Fibroblasts (A) Timetable for the derivation of ciPSCs. (B) Representative images of transfected fibroblasts and primary colonies. (C) RT-PCR analysis of PSC markers in K9 iPSC nos. 1–3 using primers specific for endogenous canine sequences. See Note S6 for further transcriptomic analyses of canine cells. (D) AP staining of K9 iPSC no. 1. Scale bars, 500 μm. (E and F) Immunocytochemical analysis of K9 iPSC no. 1 using primary antibodies of OCT4 and SOX2 (left and center). AP staining of K9 iPSC no. 1 (right). Scale bars, 100 μm. See Note S7 for further assessment of PSC marker immunoreactivity. (G) Representative images of endodermal (SOX17-positive), mesodermal (αSMA-positive), and ectodermal (βIII tubulin-positive) cells differentiated from K9 iPSC no. 1 by *in vitro* differentiation assay (see Note S8 for *in vivo* differentiation assay). Scale bars, 100 μm. (H) Genomic PCR analysis for the detection of residual episomal vectors using specific primers for the OriP sequence (Yu et al., 2009). Primers for the canine *OCT4* locus were used as an internal control. (I) Q-banding-based karyotyping of a euploid cell from the K9 iPSC no. 1 line. High-resolution images of karyotyping are shown in Table S3. (J) Chromosome counting of K9 iPSC nos. 1–3 by G-banding. Fifty cells were used in each cell line. Green bars show euploid (2n = 78) cells. Numerics on bars show the number of counted cells (in 50 cells) harboring each number of chromosomes.

We transfected the EP-B vector set into the K9 fibroblasts, after which they were cultured in NSM following pre-expansion for 9 days (Figure 6A). As seen with the marmoset fibroblasts, primary dome-shaped colonies appeared by day 14 (Figure 6B, center), and putative iPSC colonies (termed canine iPSC [ciPSC]) emerged after culture in ESM (Figure 6B, right). Again, no colony could be derived from the transfected fibroblasts when ESM was used as the induction medium (n = 3), but colony formation was observed when using NSM for induction, although the derivation efficiency was relatively low (0–2 colonies derived from 1 × 10^6^ transfected fibroblasts, n = 6). The mechanically isolated ciPSC clones grew immortally for over 20 passages.

Using RNA extracted from three ciPSC clones (K9 iPSC nos. 1–3), we performed RT-PCR using specific primers for endogenous PSC marker genes (*clfNANOG*, *clfOCT4*, and *clfSOX2*, Figure 6C). Results confirmed that the iPSCs endogenously expressed these PSC marker genes (Figure 6C). We also performed an mRNA-seq analysis of canine iPSCs (Note S6). By immunocytochemistry, we revealed that the ciPSCs were strongly positive for OCT4, SOX2, and AP (Figures 6D–6F). We further explored culture conditions which could enhance PSC marker expression in the ciPSCs (Note S7).

Moreover, we performed *in vitro* and *in vivo* three-germ layer differentiation assays using the K9 iPSC no. 1 clone, which resulted in successful differentiation into cells of all three germ layers (Figure 6G; Note S8). Following five passages after iPSC derivation, episomal vectors were confirmed to be removed from all three ciPSC clones (Figure 6H). Furthermore, we performed karyotyping of K9 iPSC nos. 1–3 clones, and found that 56% of K9 iPSC nos. 1–2 and 64% of K9 iPSC no. 3 retained normal chromosome number (2n = 78) (Figures 6I and 6J).

Thus, we demonstrated that our reprogramming method was applicable to the dog, which belongs to the taxonomic order *Carnivora*, distinct from marmosets and humans (in *Primate*).

### Derivation of Transgene-free iPSCs from Porcine Fibroblasts

**Figure 7:**
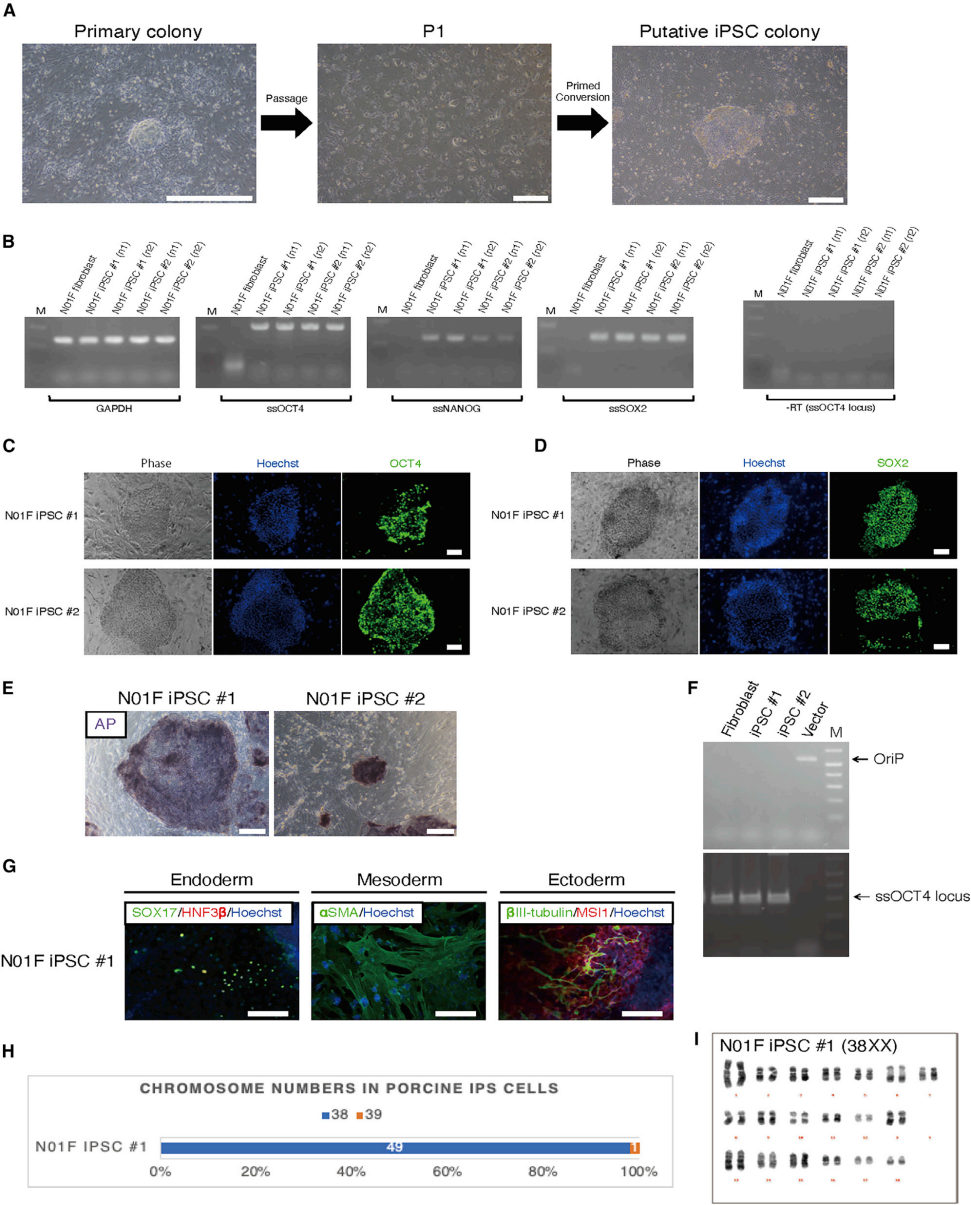
Derivation of Transgene-free iPSCs from Porcine Fibroblasts (A) Representative images of primary colonies (left), passaged cells (center), and putative iPSC colonies (right). Scale bars, 500 μm. (B) RT-PCR analysis of PSC marker genes (*OCT4*, *NANOG*, and *SOX2*) in N01F iPSC nos. 1–2 (n = 2, independent experiments) using primers specific for endogenous porcine sequences. See Note S9 for further transcriptomic analyses of porcine cells. (D and E) Immunocytochemical staining of N01F iPSC nos. 1–2 using primary antibodies of PSC markers. Scale bars, 100 μm. See Note S10 for further assessment of PSC marker immunoreactivity. (E) AP staining of N01F iPSC nos. 1–2. Scale bars, 200 μm. (F) Genomic PCR analysis for the detection of residual episomal vectors using specific primers for the OriP sequence (Yu et al., 2009). Primers for the porcine *OCT4* (*ssOCT4*) locus were used as an internal control. (G) Representative images of endodermal (SOX17, HNF3β-positive), mesodermal (αSMA-positive) and ectodermal (βIII-tubulin, MSI1-positive) cells differentiated from N01F iPSC no. 1 by *in vitro* differentiation assay. Scale bars, 100 μm. (H) Chromosome counting of N01F iPSC no. 1 by G-banding. Fifty cells were used in each cell line. Blue bars show euploid (2n = 38) cells. Numerics on bars show the number of counted cells (in 50 cells) harboring each number of chromosomes. (I) Q-banding-based karyotyping of a euploid cell from the N01F iPSC no. 1 line. High-resolution images of karyotyping are shown in Table S3.

Next, we assessed whether our reprogramming method was applicable to ear skin-derived fibroblasts of a post-neonatal pig (named N01F). We transfected the EP-B vector set to the N01F fibroblasts. Using NSM, we observed the emergence of primary dome-shaped colonies with 0.028% ± 0.012% efficiency (n = 6; Figure 7A, left). After passaging these cells once (Figure 7A, center), we initially tested ESM for iPSC derivation. However, the attempt was unsuccessful since the majority of the cells remained dissociated in ESM and only few colony-like structures emerged. Next, we tested ESM supplemented with activin A (10 ng/mL) and transforming growth factor β1 (10 ng/mL), which are important factors for the maintenance of primed-state pluripotency (James et al., 2005; Nichols and Smith, 2009) and a WNT inhibitor IWP2, since WNT inhibition reportedly enabled the stable maintenance of pluripotency in flat-shaped colony-forming cells (Sumi et al., 2013; Wu et al., 2015). Following 2 weeks of culture using this medium, putative iPSC colonies (termed porcine iPSC [piPSC]) appeared (Figure 7A, right). These colonies were mechanically isolated and expanded for further analyses.

Using RNA extracted from two piPSC clones (N01F iPSC nos. 1–2), we confirmed endogenous expression of PSC markers (*ssOCT4*, *ssNANOG*, and *ssSOX2*) with specific primers for porcine sequences (Figure 7B), and other PSC and NSC marker expression in porcine iNSLCs and iPSCs was further assessed by mRNA-seq (Note S9). AP staining and immunocytochemical analyses showed that the piPSCs strongly expressed AP, OCT4, and SOX2 (Figures 7C–7E), while these cells were negative for NANOG, SSEA4, and TRA-1-60 (Note S10), as well as SSEA1, SSEA3, and TRA-1-81 (data not shown). As performed in ciPSCs (Note S7), we explored culture conditions which could enhance PSC marker expression in the piPSCs (see Note S10). In addition, after five passages, we confirmed the removal of episomal vectors by genomic PCR (Figure 7F). Furthermore, *in vitro* differentiation of one iPSC clone (N01F iPSC no. 1) resulted in successful differentiation of the cells into all three germ layers (Figure 7G). The karyotype of N01F iPSC no. 1 was highly stable, most of analyzed cells (49 out of 50) showed normal karyotype, 38XX (Figures 7H and 7I).

## Discussion

In this study, we generated transgene-free iPSCs from fibroblasts of multiple mammalian species. Using our reprogramming method, we were able to obtain transgene-free iPSCs from both embryonic and adult marmosets, an adult dog, and post-neonatal pigs. We also demonstrated that the resultant iPSCs were successfully differentiated into all three germ layers and germ cell linage. Thus, this method is robust and efficient, and applicable for reprogramming somatic fibroblasts from various mammalian species across different taxonomic orders into iPSCs.

The naive human and marmoset PSCs we recently reported (Kisa et al., 2017; Shiozawa et al., 2020) were characterized by the strong expression of *ESRRB*, which has an important role for the maintenance of the naive pluripotent state in murine ESCs (Festuccia et al., 2012). However, when utilizing the medium for inducing these naive human PSCs in this study, the primary dome-shaped colonies that initially appeared after the transfection of fibroblasts (Figures 1B, 1D, 6B, and 7A) were not naive or primed-state PSCs (Nichols and Smith, 2009), but were presumably NSC-like cells, as shown through multiple analyses.

Collectively, marmoset iNSLCs showed unique properties of gene expression and differentiation capacity similar to NSCs, but clearly distinct from marmoset PSCs. We discuss two rational possibilities that enabled us to obtain transgene-free iPSCs from somatic fibroblasts via an NSC-like state in this study (see Note S11).

The use of this method via an NSC-like state enabled the derivation of marmoset iPSCs completely free of transgenes, which has not been achieved in earlier reports (Debowski et al., 2015; Tomioka et al., 2010; Wu et al., 2010). More recently, we and other groups reported the generation of transgene-free marmoset iPSCs by episomal vectors or RNA-based reprogramming, using chemical inhibitors similar to those supplemented in NSM (Nakajima et al., 2019; Petkov et al., 2020; Vermilyea et al., 2017; Watanabe et al., 2019), but the reprogramming mechanism has not been thoroughly investigated. This study suggests that passing through this NSC-like state facilitates iPSC reprogramming for marmoset fibroblasts. In addition, in sharp contrast to the previous reports, our study confirmed the efficacy of the iNSLC-mediated reprogramming of somatic fibroblasts in species besides the marmoset. In particular, successful derivation of transgene-free ciPSCs and piPSCs is significant, due to the species-specific difficulties in previous studies (see Note S12).

The definition of bona fide iPSCs remains controversial. Tetraploid complementation is the most stringent criterion for evaluating the developmental potential of murine iPSCs (Wernig et al., 2007). Less stringently and more practically, the potential for germline-transmitting chimera formation through blastocyst injection has also been used as a developmental criterion for murine iPSCs (Hamanaka et al., 2011; Okita et al., 2007). However, as there are few reports of non-rodent mammalian iPSCs that are germline-competent, except for one on piPSCs (West et al., 2011), and none on primates, including humans, there is a need for an alternative criterion for these species. In this context, several studies reported that transgene excision in iPSCs seemed crucial for normal development *in vivo* (Du et al., 2015; Okita et al., 2007; West et al., 2011), and transgene-excised hiPSCs have been suggested to be "safer" than transgene-integrated ones, as the reactivation of transgenes can increase tumorigenic risk (Galat et al., 2016). Thus, we propose the absence of transgene(s) to be a tentative criterion for bona fide non-rodent mammalian iPSCs.

In conclusion, we obtained transgene-free iPSCs fulfilling the criterion above in three species, spanning various taxonomic orders. Our method described in this study may facilitate the reprogramming process in the class *Mammalia*.

## Experimental procedures

### Animals and Ethical Statements

All animal experiments were performed in accordance with the guidelines for laboratory animals by the National Institutes of Health, and the Ministry of Education, Culture, Sports, Science and Technology (MEXT) of Japan, and were approved by the institutional Animal Care and Use Committee of Keio University, Nihon University, and RIKEN (approval no. H27-2-306(4)).

Animal care was conducted in accordance with the National Research Council (NRC) Guide for the Care and Use of Laboratory Animals (2011).

Other experimental procedures, including information of animals, cell culture, genomic and transcriptomic analyses, are described in Supplemental Experimental Procedures.

## Author contributions

Conceptualization, S.Y.; Methodology, S.Y.; Software, S.Y., M. Nakajima, T. Sanosaka, and K.I.; Validation and Format Analysis, S.Y., M. Nakajima, A.I., and T. Sato; Investigation and Resources, S.Y., A.I., T. Sanosaka, R.N., M.I., H.W., J.O., Y.T., E.A., E.S., R.B., T.N., K.E., and S.S.; Data Curation, S.Y., M. Nakajima, and T.Sanosaka; Writing – Original Draft, S.Y.; Writing – Review & Editing, S.Y., M. Nakajima, T. Sanosaka, M. Nakamura, T.N., and H.O.; Supervision and Project Administration, H.O.; Funding Acquisition, H.O., K.E., and S.Y.

## Declaration of interests

H.O. serves as a paid scientific advisor at SanBio Co. Ltd. and K Pharma Inc., but these companies had no control over this work. The other authors declare neither financial nor non-financial competing interests.

## Supplemental information

Document S1. Supplemental Experimental Procedures, Figures S1–S7, and Notes S1–S12

Table S1. Differential Expressed Genes (PGCLC Versus PSC)

Table S2.Differential Expressed genes (PSC Versus iNSLC)

Table S3.Q-banding of PSCs

Document S2. Article plus Supplemental information
