# Supplementary material for: Non-viral Induction of Transgene-free iPSCs from Somatic Fibroblasts of Multiple Mammalian Species
Source: Stem Cell Reports. 2021 Apr 1;16(4):754–70. doi: 10.1016/j.stemcr.2021.03.002 (PMC8072067; doi:10.1016/j.stemcr.2021.03.002)
Supplement: Document S2. Article plus Supplemental information [file mmc5.pdf]

Stem Cell Reports
Article
OPEN ACCESS

Non-viral Induction of Transgene-free iPSCs from Somatic Fibroblasts of Multiple Mammalian Species

Sho Yoshimatsu,[1,2,3,*] Mayutaka Nakajima,[1] Aozora Iguchi,[4] Tsukasa Sanosaka,[1] Tsukika Sato,[1] Mari Nakamura,[1] Ryusuke Nakajima,[3] Eri Arai,[5] Mitsuru Ishikawa,[1] Kent Imaizumi,[1] Hirotaka Watanabe,[1] Junko Okahara,[3,6] Toshiaki Noce,[3] Yuta Takeda,[3] Erika Sasaki,[3,6] Rüdiger Behr,[7,8] Kazuya Edamura,[4] Seiji Shiozawa,[1] and Hideyuki Okano[1,3,*]

[1]Department of Physiology, School of Medicine, Keio University, 35 Shinanomachi, Shinjuku-ku, Tokyo, Japan
[2]Laboratory for Proteolytic Neuroscience, RIKEN Center for Brain Science, Saitama, Japan
[3]Laboratory for Marmoset Neural Architecture, RIKEN Center for Brain Science, Saitama, Japan
[4]Laboratory of Veterinary Surgery, Department of Veterinary Medicine, College of Bioresource Sciences, Nihon University, Kanagawa, Japan
[5]Department of Pathology, School of Medicine, Keio University, Tokyo, Japan
[6]Department of Marmoset Biology and Medicine, Central Institute for Experimental Animals, Kanagawa, Japan
[7]Research Platform Degenerative Diseases, German Primate Center – Leibniz Institute for Primate Research, Göttingen, Germany
[8]DZHK (German Centre for Cardiovascular Research), partner site Göttingen, Göttingen, Germany
*Correspondence: yoshima@a7.keio.jp (S.Y.), hidokano@keio.jp (H.O.)
https://doi.org/10.1016/j.stemcr.2021.03.002

SUMMARY

Induced pluripotent stem cells (iPSCs) are capable of providing an unlimited source of cells from all three germ layers and germ cells. The derivation and usage of iPSCs from various animal models may facilitate stem cell-based therapy, gene-modified animal production, and evolutionary studies assessing interspecies differences. However, there is a lack of species-wide methods for deriving iPSCs, in particular by means of non-viral and non-transgene-integrating (NTI) approaches. Here, we demonstrate the iPSC derivation from somatic fibroblasts of multiple mammalian species from three different taxonomic orders, including the common marmoset (*Callithrix jacchus*) in *Primates*, the dog (*Canis lupus familiaris*) in *Carnivora*, and the pig (*Sus scrofa*) in *Cetartiodactyla,* by combinatorial usage of chemical compounds and NTI episomal vectors. Interestingly, the fibroblasts temporarily acquired a neural stem cell-like state during the reprogramming. Collectively, our method, robustly applicable to various species, holds a great potential for facilitating stem cell-based research using various animals in *Mammalia*.

INTRODUCTION

Embryonic stem cells (ESCs), derived from the inner cell mass of pre-implantation blastomeres, have potentials for unlimited proliferation by self-renewal and for differentiation into all three germ layers and germ cells (Smith, 2001). As such, ESCs have been considered to be in a "pluripotent" state, referred as pluripotent stem cells (PSCs). The first demonstration of ESC derivation was performed with mice (Evans and Kaufman, 1981; Martin, 1981), and subsequently with non-human primates (NHPs) (Thomson et al., 1995) and humans (Thomson et al., 1998). However, ethical concerns and resource limitations have been imposed on the usage of early blastomeres from several mammalian species, including NHPs and humans. Moreover, the maintenance of *in vitro* culture of early-stage embryos remains challenging, especially for many wildlife mammalian species (Cordova et al., 2017). These circumstances emphasize the necessity of other species-wide approaches for obtaining PSCs.

Alternatively, an unlimited source of cells can be derived from induced PSCs (iPSCs) without ethical and practical limitations. Reprogramming of somatic fibroblasts into iPSCs has been demonstrated in mice (Takahashi and Yamanaka, 2006) and in humans (Takahashi et al., 2007) by the ectopic overexpression of defined factors, such as *OCT4*, *SOX2*, *KLF4*, and *C-MYC*. The resultant iPSCs have a wide range of applicability for disease modeling *in vitro* and for regenerative medicine (Fujimori et al., 2018; Okano and Yamanaka, 2014; Yamanaka, 2012). In addition, developmental studies have proven that iPSCs have a potential for giving rise to new offspring, similarly to ESCs (Bradley et al., 1984). This has been verified in studies with rodents and pigs, in which germline-transmitting chimera formation was achieved through blastocyst injection (Hamanaka et al., 2011; Honda et al., 2017; Okita et al., 2007; Thomas and Capecchi, 1987; Wernig et al., 2007; West et al., 2011) or by directly inducing functional mature gametes (Hayashi et al., 2011, 2012). For over 10 years, the reprogramming technology has been performed and validated in a variety of mammalian species, including great apes (Marchetto et al., 2013), farm animals (Ogorevc et al., 2016), and endangered species (Ben-Nun et al., 2011; Honda et al., 2017). Moreover, iPSCs derived from various species have paved the way for evolutionary studies assessing species differences *in vitro* (Marchetto et al., 2013).

Although the definition of bona fide iPSCs remains elusive, it is well-known that fully reprogrammed iPSCs sustain a pluripotent state in the absence of transgene expression (Okita et al., 2007). At the dawn of iPSC

This is an open access article under the CC BY license (http://creativecommons.org/licenses/by/4.0/).

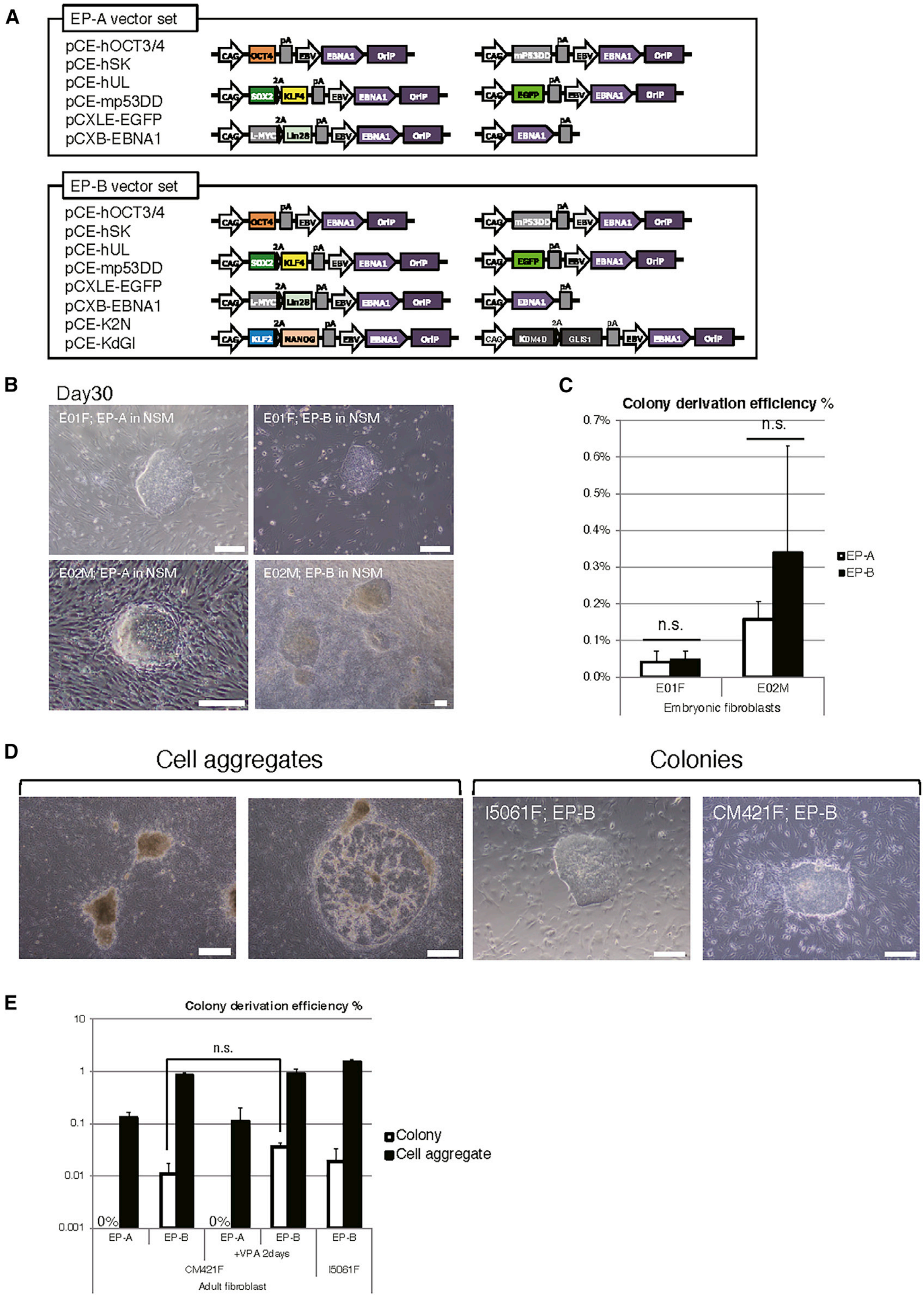

Figure 1. Derivation of Primary Colonies from Marmoset Fibroblasts Using Episomal Vectors

(A) Schematic of plasmids used for vector transfection.

(B) Representative images of primary colonies derived from E01F and E02M fibroblasts using NSM at day 30. Scale bars, 200 μm.

(C) Derivation efficiency of primary colonies from E01F and E02M fibroblasts using NSM (n = 3, independent experiments).

(legend continued on next page)

reprogramming, transgene-integrating retroviruses were used for deriving iPSCs (Takahashi et al., 2007; Takahashi and Yamanaka, 2006; Wernig et al., 2007; Yu et al., 2007) and the transgenes were gradually silenced after the iPSCs were fully reprogrammed. However, viral transduction raised concerns for the clinical application of iPSCs. Yu et al. (2009) were the first to report the derivation of iPSCs using non-viral, non-transgene-integrating (NTI) episomal vectors, enabling the generation of transgene-free iPSCs. Since residual transgenes in the iPSCs restrict their utility for *in vivo* and *in vitro* differentiation (Nair, 2008; Okita et al., 2007) and for the generation of iPSC-derived offspring (Du et al., 2015; Maherali et al., 2007; Okita et al., 2007; Wernig et al., 2008), NTI approaches for iPSC derivation have been intensively studied in recent years. Although complete transgene excision from the iPSCs has been demonstrated in rodents (Li et al., 2017; Wu et al., 2014) and in humans (Okita et al., 2011, 2013; Yu et al., 2009), it still remains a challenge in other species.

In this study, we demonstrated the derivation of transgene-free iPSCs from somatic fibroblasts of multiple mammalian species from three different taxonomic orders, including the common marmoset (marmoset; *Callithrix jacchus*) in *Primates*, the dog (*Canis lupus familiaris*) in *Carnivora*, and the pig (*Sus scrofa*) in *Cetartiodactyla*, by combinatorial usage of small molecules and NTI episomal vectors. We also demonstrated the differentiation potential of these iPSCs into all three germ layers and primordial germ cell-like cells (PGCLCs). Interestingly, during the reprogramming process, we observed that the primary colony-forming cells showed neural stem cell (NSC)-like characteristics, which could be sustained over time when the cells were cultured in the same medium used for induction of these cells. Our data suggest that the reprogramming method would be invaluable for deriving transgene-free iPSCs from somatic fibroblasts of various mammalian species.

RESULTS

Derivation of Primary Colonies from Marmoset Fibroblasts Using Episomal Vectors

First, we attempted to assess the derivation efficiency of primary colonies from dorsal skin-derived fibroblasts of embryonic marmosets (named E01F and E02M) using a set of Epstein-Barr virus *EBNA1*- and *OriP*-based episomal vectors (Okita et al., 2013) encoding five reprogramming factors (human *OCT4*, *SOX2*, *KLF4*, *L-MYC*, and *LIN28*), a dominant-negative mutant of mouse *Trp53* (*mp53DD*), and enhanced green fluorescent protein (EGFP) for assessing the transfection efficiency (collectively named the "EP-A vector set," Figure 1A, top), which is a conventional set of vectors used for deriving human iPSCs (hiPSCs) (Okita et al., 2013). These vectors were delivered into the fibroblasts by electroporation, and the transfection efficiency was calculated to be between 7% and 35% according to the EGFP fluorescence (Figure S1A). After transfection, the fibroblasts were expanded for 3–7 days in a fibroblast medium (M10), and then transferred onto mouse embryonic feeder cells in an induction medium.

However, when using a basic-fibroblast growth factor and knockout serum replacement-based ESC medium (ESM) as the induction medium (Nii et al., 2014; Yoshimatsu et al., 2019a), no colonies appeared from the E01F and E02M fibroblasts 30 days after transfection (n = 3; Figure S1B). This suggested that the conventional method for generating hiPSCs (Okita et al., 2011) was not applicable to marmoset cells. Next, we decided to utilize a medium we previously reported for inducing and maintaining putative naive-state hiPSCs and marmoset ESCs from conventional primed-state cells (termed as NSM) (Kisa et al., 2017; Shiozawa et al., 2020). Using NSM as the induction medium, we observed primary dome-shaped colonies from both E01F and E02M embryonic fibroblasts 30 days after transfection (Figure 1B, left). We then either mechanically isolated the primary colonies for clonal expansion or expanded them in bulk in NSM for further analyses. The colony derivation efficiency from EGFP-positive fibroblasts using the EP-A vector set was calculated to be between 0.040% for the E01F fibroblasts and 0.157% for the E02M fibroblasts (Figure 1C).

We also attempted to derive colonies from ear skin-derived fibroblasts of an adult marmoset (named CM421F). However, only cell aggregates were obtained from the transfected fibroblasts when using EP-A (Figure 1D, left). We speculated that the failure was due to low reprogramming efficiency and, therefore, we tested for additional factors to enhance the efficiency.

Since we previously demonstrated that the combinatorial usage of NSM and the overexpression of six factors (*OCT4*, *SOX2*, *KLF4*, *C-MYC*, *KLF2*, and *NANOG*) can convert the primed state of hiPSCs and marmoset ESCs into a naive-like state (Kisa et al., 2017; Shiozawa et al., 2020), we added an episomal vector, pCE-K2N, which harbors two of these factors (*KLF2* and *NANOG*), to the EP-A vector set. Although we were successful in obtaining

(D) Representative images of cell aggregates (left) and primary colonies (right) derived from I5061F and CM421F fibroblasts. Scale bars, 500 μm (left), 200 μm (right).
(E) Derivation efficiency of primary colonies (white box) and cell aggregates (black box) from I5061F and CM421F fibroblasts (n = 3, independent experiments). n.s., not significant.

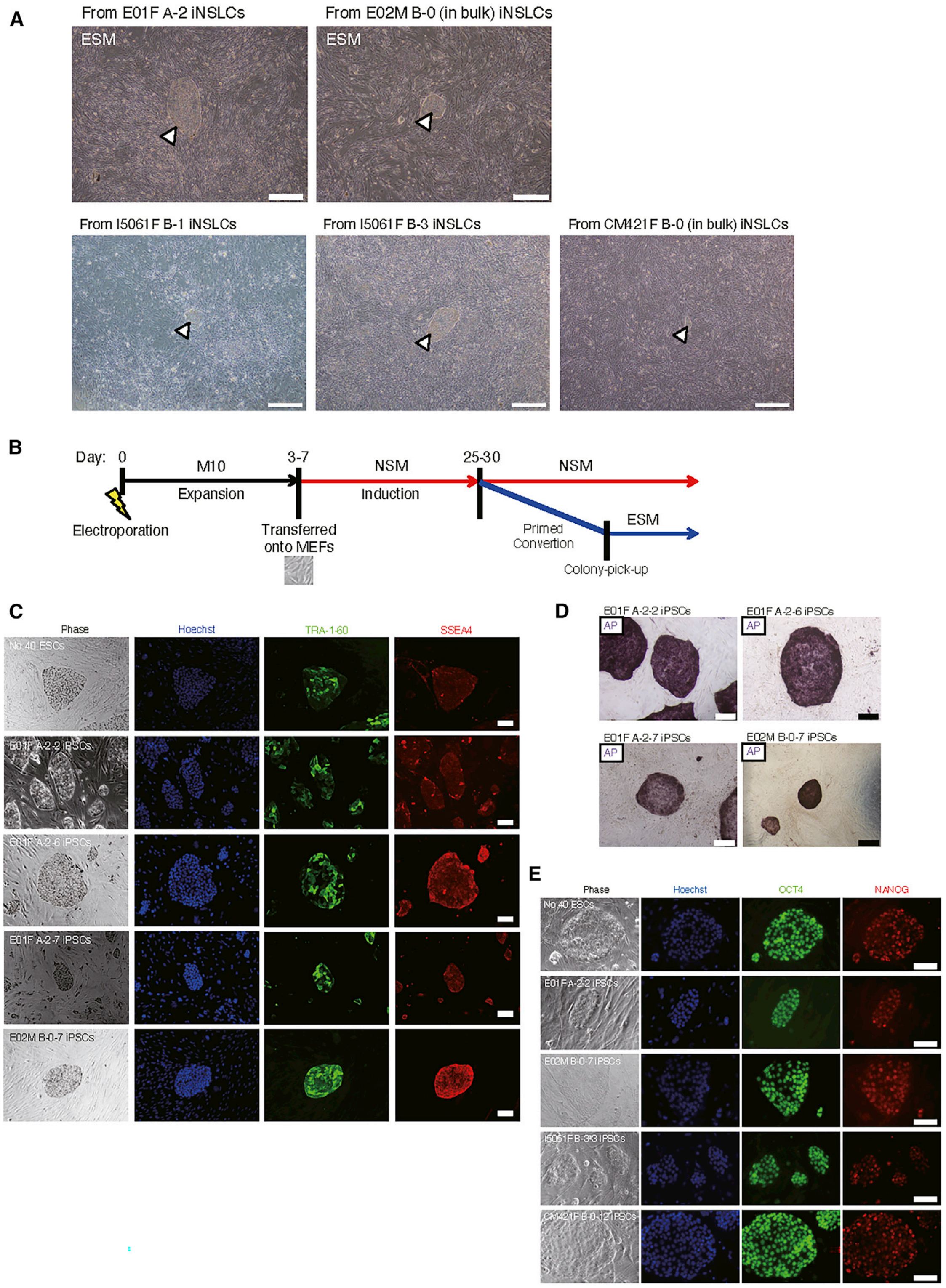

Figure 2. Derivation of Marmoset Transgene-free iPSCs

(A) Representative images of putative iPSC colonies. Following primed conversion of iNSLCs, ESC-like (iPSC) colonies appeared (white arrowhead).

(B) Timetable for the derivation of marmoset iPSCs. Day 0 was defined as the timing of vector transfection.

(legend continued on next page)

primary colonies using NSM with the updated vector set, the colonies were unable to reach confluency after they were passaged or mechanically picked up.

Therefore, we further tested for two additional factors: *GLIS1* for enhancing the reprogramming efficiency (Maekawa et al., 2011), and *KDM4D* for facilitating epigenetic reprogramming (Liu et al., 2018; Matoba et al., 2014). These two factors were introduced into an episomal vector, pCE-KdGl. The new vector set with both pCE-K2N and pCE-KdGl added to the EP-A vector set was named EP-B (Figure 1A, bottom). Using EP-B, we succeeded in deriving primary colonies from ear skin-derived fibroblasts of two adult marmosets (CM421F and I5061F) 30 days after transfection (Figure 1D, right). However, unlike the culture derived from embryonic fibroblasts (Figure 1B), cell aggregates were the major population in the culture derived from adult fibroblasts (Figure 1E). In addition, we evaluated the effect of the supplementation of valproic acid (VPA), a histone deacetylase inhibitor, to the induction medium for 2 days, which was previously used for the derivation of marmoset iPSCs (Debowski et al., 2015). In our culture method, the derivation efficiency of primary colonies was not significantly enhanced with VPA supplementation (Figure 1E).

When using EP-B, we also succeeded in deriving primary colonies from embryonic fibroblasts cultured in NSM (Figure 1B, right), but not in ESM (0%, n = 3). Unlike adult fibroblasts, the colony derivation efficiencies from embryonic fibroblasts were not significantly different with EP-A or EP-B (Figure 1C).

Although the primary colony-forming cells derived from marmoset fibroblasts showed ubiquitous expression of SOX2 (Figure S1C, top) and alkaline phosphatase (AP) (Figure S1D), these cells were negative for PSC markers, such as TRA-1-60 and SSEA4 (Figure S1E). Alternatively, we found that these cells expressed markers of NSCs, such as MSI1 (Sakakibara et al., 1996) and PAX6 (Figures 4 and S6). For that reason, we tentatively named the primary colony-forming cells cultured in NSM as putative iNSLCs (induced NSC-like cells).

Derivation of Marmoset Transgene-free iPSCs

Since the iNSLCs were TRA-1-60 and SSEA4 negative, we next attempted to investigate their potential to convert into PSCs by culturing these cells in ESM, which is routinely used for culturing marmoset ESCs. Surprisingly, ESC-like colonies emerged after culturing the iNSLCs in ESM for 3 weeks (Figure 2A). On average, from sub-confluent iNSLCs at early passages (P1–2) in one well of a 6-well plate (~1 × 10^6 cells), ~50 ESC-like colonies appeared. This step was termed as "primed conversion" (Figure 2B). In contrast, iNSLCs at late passages (P4–6) were not competent for primed conversion (from over 6 × 10^6 cells, n = 3). The ESC-like colonies were then mechanically isolated, followed by clonal expansion in ESM for further analyses. Since the morphology of the ESC-like cells cultured in ESM were indistinguishable from that of conventional marmoset ESCs (Sasaki et al., 2005), showing a tightly packed colony structure with defined borders and high nuclear/cytoplasm rate, we termed these cells as putative marmoset iPSCs.

Immunocytochemical analysis of the putative iPSCs resulted in a similar staining pattern of TRA-1-60 and SSEA4 to that of the no. 40 marmoset ESC clone (Sasaki et al., 2005) (Figures 2C and S2B). They were also positive for AP (Figures 2D and S2A), OCT4, and NANOG (Figure 2E). On the other hand, they were not positive for other PSC markers, including SSEA1 and SSEA3, as shown previously (Yoshimatsu et al., 2020), or for NSC markers, including MSI1 and PAX6 (data not shown). To confirm whether the putative iPSCs expressed PSC marker genes endogenously, we performed quantitative reverse-transcriptiase PCR (qRT-PCR) using specific primers for endogenous mRNA sequences of marmoset *OCT4*, *NANOG*, *SOX2*, *KLF4*, *ZFP42* (*REX1*), *LIN28* (*LIN28*A), *DPPA5*, and *TERT* genes. While fibroblasts only expressed *KLF4*, the putative iPSCs expressed all of the analyzed PSC marker genes endogenously (Figures S2C–S2H).

At 9–14 passages after the derivation of the putative iPSCs, most clones (5/6 of E01F, 1/2 of E02M, 9/9 of I5061F and, 4/4 of CM421F) showed successful removal of all the episomal vectors, which was confirmed by genomic PCR (Figures S3A–S3D) using highly specific and sensitive primers for the OriP sequence (Yu et al., 2009) (Figure S3E). We also designed and validated specific primers for each episomal vector (Figures S3F and S3G), and confirmed their removal in four representative iPSC clones, including those from two embryonic marmosets (E01F A-2-2 and E02M B-0-7) and two adult marmosets (I5061F B-3-3 and CM421F B-0-12) (Figure S3H), while the parental, primed conversion-competent iNSLCs at an early passage (P1) harbored all episomal vectors (Figure S3I). We performed chromosome counting by G-banding of six representative iPSC lines (Figure S4A), and found 46% of CM421F B-0-4, 88% of I5061F B-3-3, 88% of E02M B-0-7, 84% of E01F A-2-7, 58% of E01F A-2-6, and 76% of E01F A-2-2 retained normal chromosome number (2n = 46),

(C) Immunocytochemical staining of iPSCs using TRA-1-60 and SSEA4 antibodies. No. 40 ESCs were used as positive controls (top). Scale bars, 100 μm. See Figure S2B for iPSC clones derived from adult marmosets.
(D) AP staining of iPSC colonies. Scale bars, 500 μm. See Figure S2A for iPSC clones derived from adult marmosets.
(E) Immunocytochemical staining of iPSCs using OCT4 and NANOG antibodies. Scale bars, 100 μm.

and these euploid cells showed normal karyotype, analyzed by Q-banding (Figure S4B). Furthermore, we confirmed *in vitro* and *in vivo* three-germ layer differentiation potentials of the putative iPSC lines (Figures 3A–3D).

In addition, we explored the differentiation potential of marmoset iPSCs into germ cell linage by the combinatorial usage of cytokines (Hayashi et al., 2011) and transcription factors (Kobayashi et al., 2017) for *BLIMP1-Venus* knockin E01F A-2-2 iPSCs (Figures S5A and S5B). By using an optimized PGCLC induction method for marmoset ESCs (Figures S5C and S5D; Note S1), the iPSCs were differentiated into Venus-positive PGCLCs (Figures S5E–S5I; Note S1; Table S1), whose transcriptome was comparable to those of PGCLCs derived from reporter knockin marmoset ESCs (Yoshimatsu et al., 2019b, 2020).

In sum, the putative iPSCs retained normal karyotypes, and acquired and maintained pluripotency, which was independent of transgene expression. Furthermore, they possessed the capacity to differentiate into tissues of all three germ layers and PGCLCs. Thus, these putative iPSCs will be referred to as transgene-free iPSCs for the subsequent results described below.

Characterization of the Marmoset iNSLCs

During our reprogramming procedure (Figure 2B), we initially obtained primary colonies considered to be putative iNSLCs, which stained positively for NSC markers, such as SOX2, PAX6, and MSI1, but negatively for TRA-1-60 and SSEA4. We used six marmoset iNSLC clones (E01F A-2, E02M B-4, E02M B-12, E02M B-23, I5061F B-3, and CM421F B-4) for further analyses to confirm that the iNSLCs represented an NSC-like state distinct from the transgene-free iPSCs. Although we showed that the iNSLCs were continuously expandable even after ten passages (Figure 4A) with sustained ubiquitous MSI1 expression (Figure 4B), episomal vectors persisted to remain in the cells (Figure 4C). By qPCR, we revealed that PSC marker genes (*OCT4, NANOG, KLF4, ZFP42*, and *DPPA5*) were not endogenously expressed in the iNSLCs (at passages 4–6), except for *SOX2* and *TERT* (Figures 4D and 4E). Meanwhile, exogenous expression of *OCT4, NANOG, KLF4*, and *LIN28* from the episomal vectors was confirmed (Figure 4F).

Next, we assessed the expression of cell lineage marker genes for elucidating the biological and developmental characteristics of the iNSLCs. By qPCR, we found that the iNSLCs strongly expressed *PAX6* (Figure 4G), whose expression is required for the self-renewal and neurogenesis of NSCs (Sansom et al., 2009). The early ectodermal markers *ZFP521* (Kamiya et al., 2011) and *SOX1* were also expressed in the iNSLCs (Figure S6A). On the other hand, we detected no or very low expression of early mesodermal or endodermal markers, such as *T* and *SOX17*, in these cells, which was even lower than that of the ESCs (Figure S6B).

We also analyzed gene expression of the primed conversion-competent iNSLCs at early passages (P1–2) (Figure 2B). The bulk iNSLCs derived from I5061F fibroblasts using EP-B (I5061F B-0 iNSLCs) showed strong endogenous expression of *SOX2* and *PAX6* (Figures S6C and S6D), while there was only exogenous expression of PSC markers, such as *OCT4* (data not shown). In addition, immunocytochemical analysis revealed that that ~10% doublecortin-positive putative neuroblasts were present in the iNSLC colonies (Figure S6E). Meanwhile, PAX6 and SOX2 were ubiquitously expressed in the iNSLCs (Figure S6F). Furthermore, we confirmed the high neurogenic potential of the iNSLCs by direct differentiation assays (Note S2; Figures S6H–S6L) (Yoshimatsu et al., 2019a).

Global Gene Expression Profiling of the Marmoset iPSCs and iNSLCs

In this study, we initially derived iNSLCs from marmoset fibroblasts, after which they were converted into iPSCs. To elucidate the global differences and similarities of gene expression among these cells, we performed transcriptomic analyses by 3′IVT microarray (Note S3) and mRNA sequencing (mRNA-seq) (Figures 5A and 5B).

By bulk mRNA-seq analysis of marmoset samples, including fibroblasts (E01F, E02M, I5061F, and CM421F fibroblasts), EP-B-transfected fibroblasts cultured in NSM for induction (induced; E01F fibroblast EPB NSM days 6 and 15), fibroblast-derived iNSLCs, ESCs, fibroblast-derived iPSCs, *in-vivo*-derived neurospheres (E95 cortex neurosphere and GE [ganglionic eminence] neurosphere), and neurosphere-derived iPSCs (CTXNS1 B-1 and CTXNS2 B-1; see the next section for details). In addition, we included previously deposited data of marmoset ESCs (cjes001) and iPSCs (DPZcj_iPSC1) (Debowski et al., 2015), morulae and peri-implantation epiblasts (Shiozawa et al., 2020), and adult marmoset cortex (Yoshimatsu et al., 2019a). Consistent with the result of the microarray analysis (Figure S7A), Principal-component analysis (PCA) of all the analyzed gene expression clearly divided PSCs, fibroblasts, early-stage embryos, and iNSLCs (Figure 5A). In addition, we performed hierarchical clustering based on the expression of major pluripotency/ectoderm-related genes (Figure 5B). We found marmoset ESCs, iPSCs, and epiblasts were clustered together, while they were segregated from early blastomeres (morulae and blastocysts), fibroblasts, *in-vivo*-derived neurospheres, and iNSLCs (Figure 5B). By differential expressed gene (DEG) analysis of marmoset iPSC and iNSLC samples, we discovered the expression of pluripotency-related genes, including *DPPA2*, *TDGF1*, *UTF1*, *ZFP42*, *EPCAM*, and *NANOG*, was significantly higher in iPSCs, while the expression of neurogenesis-related genes, including *ASCL1*, *PAX6*, *NEUROD1*, and *NEUROG1-2*, were significantly higher in iNSLCs (Figure S7A; Table S2).

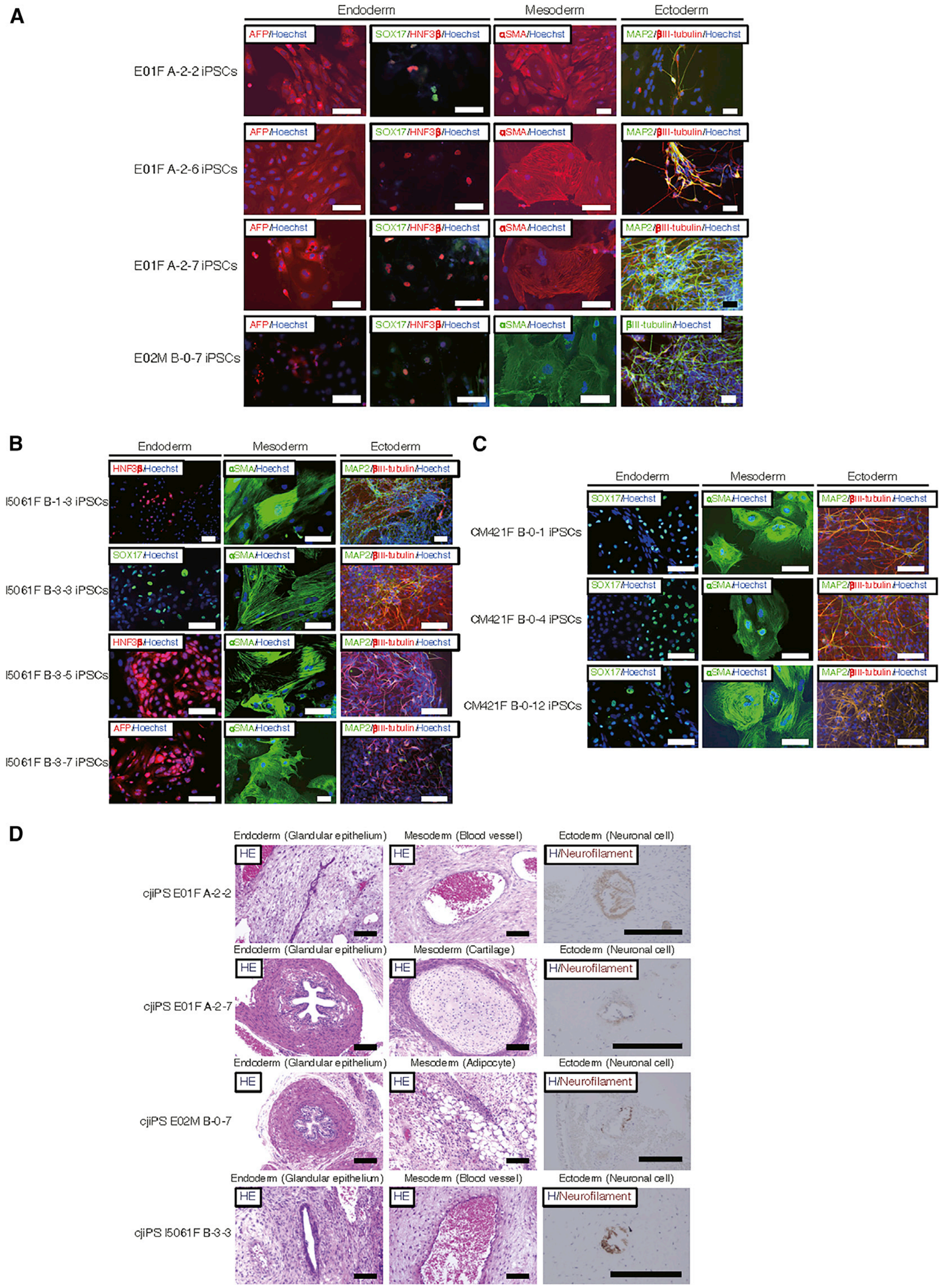

Figure 3. Three-Germ Layer Differentiation of Marmoset iPSCs

(A–C) Representative images of endodermal (AFP, HNF3β, SOX17-positive), mesodermal (αSMA-positive), and ectodermal (MAP2, βIII tubulin-positive) cells differentiated from marmoset iPSCs by *in vitro* differentiation assay. Scale bars, 100 μm.

(D) Representative images of three-germ layer tissues or cells in teratomas derived from marmoset iPSCs. H&E, hematoxylin and eosin staining; H/Neurofilament, hematoxylin staining with immunocytochemical staining using anti-neurofilament 200 kDa antibody. Scale bars, 100 μm.

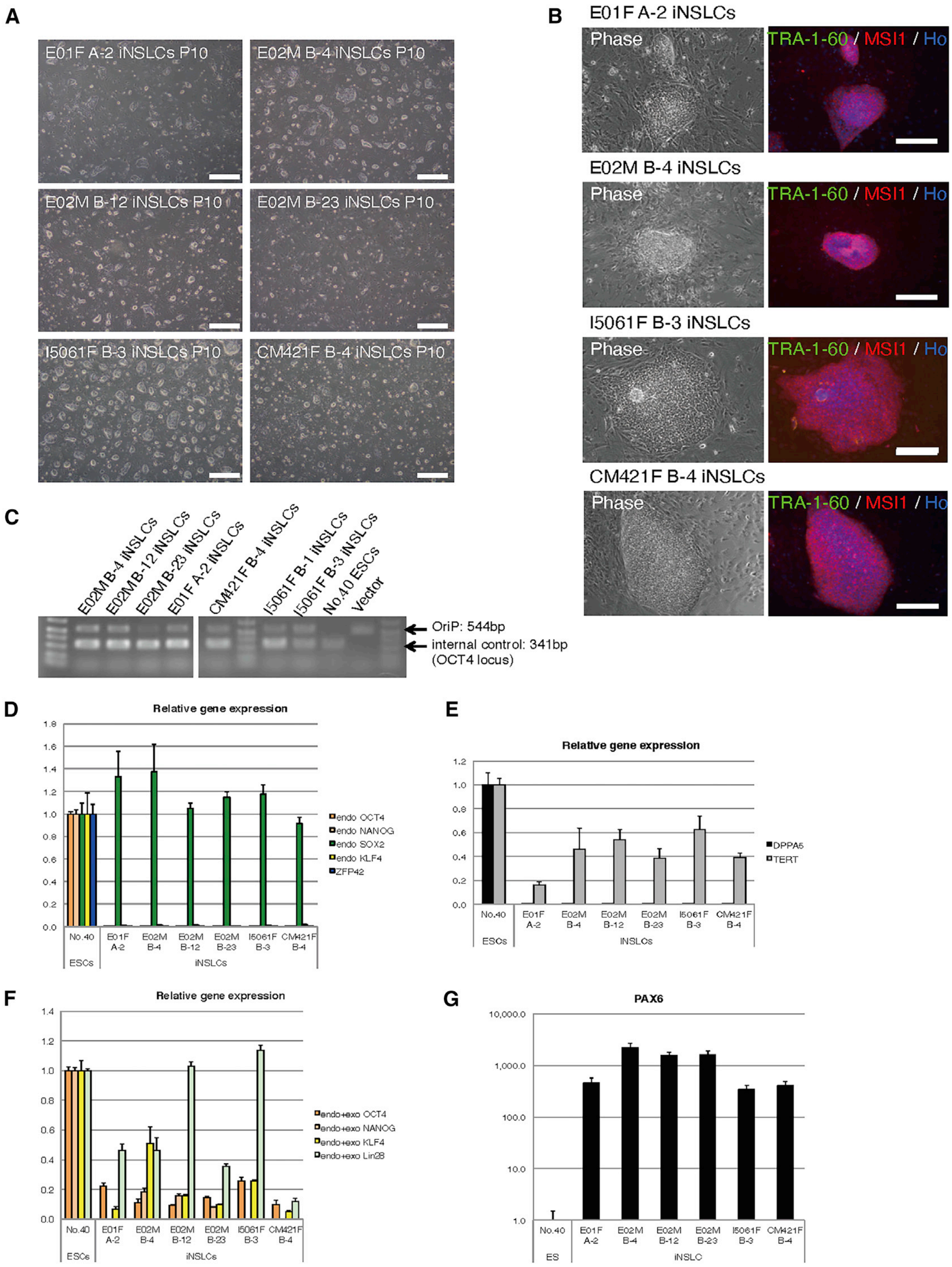

Figure 4. Characterization of the Marmoset iNSLCs

(A) Representative images of iNSLC clones cultured in NSM following ten passages (P10). Scale bars, 500 μm.

(B) Immunocytochemical staining of the iNSLCs using TRA-1-60 and MSI1 antibody. Ho, Hoechst (nuclear DNA).

(C) Genomic PCR analysis for residual episomal vectors. Episomal vectors remained in all of the derived iNSLC clones at P10.

(D–G) qPCR analysis of PSC/NSC markers in the iNSLCs. RNA extracted from iNSLCs at passages 4–6 was used (n = 3, independent experiments).

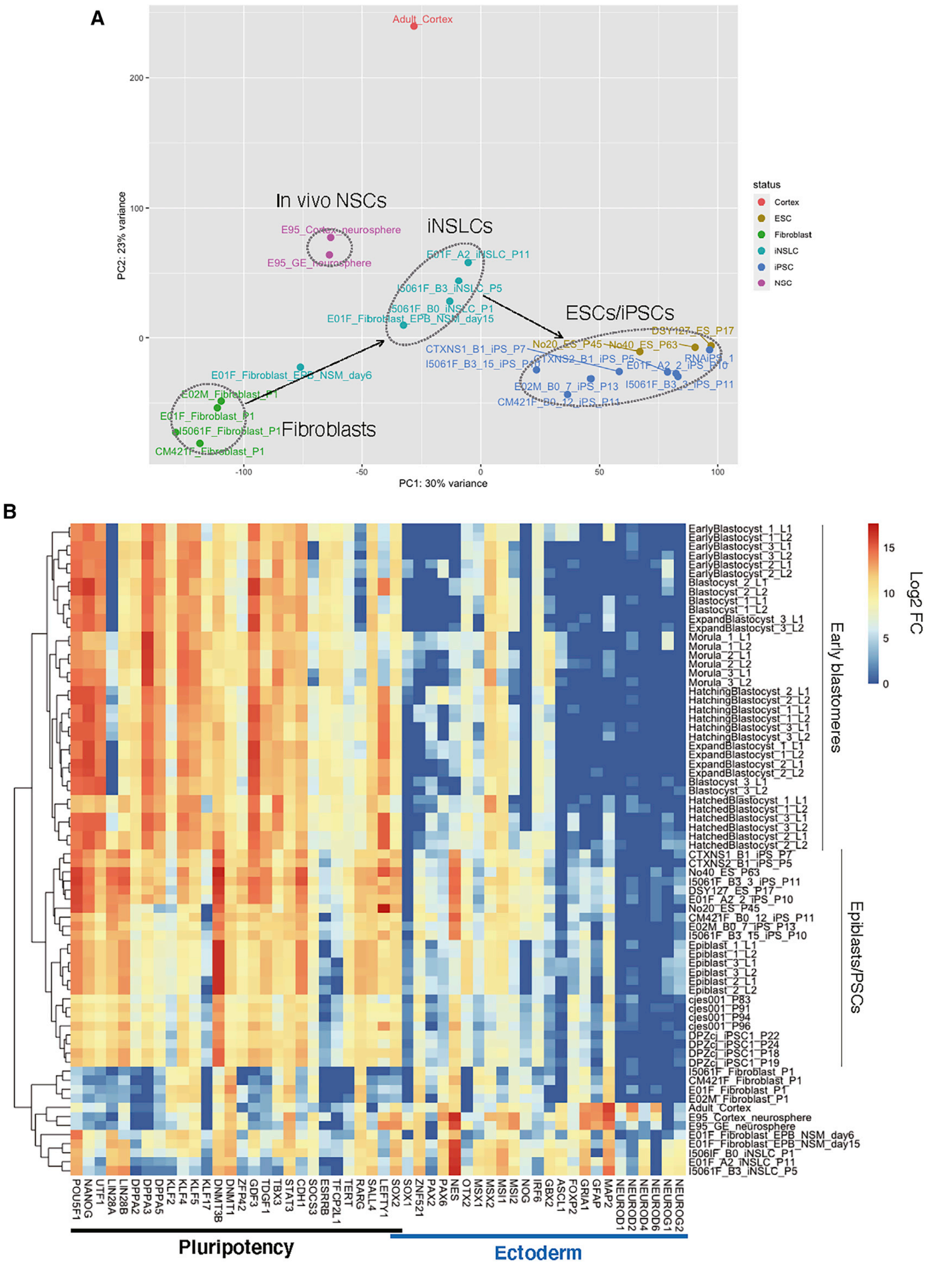

Figure 5. Bulk mRNA-Seq Analysis of Marmoset Cells

(A) PCA of marmoset samples, including fibroblasts (FB; E01F, E02M, I5061F, and CM421F fibroblasts), EP-B-transfected fibroblasts cultured in NSM for induction (induced; E01F fibroblast EPB NSM days 6 and 15), iNSLCs (E01F A-2, I5061F B-3, and I5061F B-0 iNSLCs), ESCs (no. 40 ESCs, DSY127 ESCs, and no. 20 ESCs), iPSCs (E01F A-2-2, E02M B-0-7, I5061F B-3-3, I5061F B-3-15, CM421F B-0-12, CTXNS1 B-1, and CTXNS2 B-1 iPSCs), and *in-vivo*-derived neurospheres (in_vivo_NSC; E95 cortex neurosphere and GE [ganglionic eminence] neurosphere). Black arrows show the pseudo trajectories of the reprogramming (fibroblasts to iNSLCs, and iNSLCs to iPSCs).

(legend continued on next page)

To explore the heterogeneity of the early-passage iNSLCs that were competent for primed conversion, we performed single-cell random displacement amplification sequencing (RamDA-seq) analysis (Hayashi et al., 2018). As shown in Figure S7B, we discovered that primed conversion-competent E01F A-2 iNSLCs at an early passage (P1) were clearly segregated from no. 40 ESCs and E01F A-2-2 iPSCs by PCA (iNSLCs: n = 65; ESCs/iPSCs: n = 5). However, based on the respective DEGs, we found each iNSLC showed a diverse gene expression profile, such as neurogenesis-related genes, were not highly expressed in all iNSLCs (Figure S7C), and a small population of iNSLCs showed the expression of *EPCAM*, *ZFP42*, and *DPPA2* (Figure S7D), which were estimated to be specifically expressed in iPSCs by the bulk mRNA-seq analysis (Figures 5B and S7A).

Taken together, the combination of qPCR, immunocytochemistry, and transcriptomic analyses confirmed that the iNSLCs and the transgene-free iPSCs are in two distinct cellular states. Thus, we have developed a unique reprogramming protocol for deriving transgene-free iPSCs from marmoset fibroblasts through an NSC-like state, although single-cell analysis showed that a small population of iNSLCs may already have acquired a pluripotency-directed propensity.

Reprogramming of *In-Vivo*-Derived Neural Stem Cells toward a Pluripotent State Using ESM

We demonstrated that the marmoset iNSLCs possessed a unique property to be easily re-reprogrammed toward pluripotency, which can be explained by the expression of the residual transgenes in these cells and the fact that they are transcriptionally similar to *in-vivo*-derived NSCs, which were reported to have a higher potential to be reprogrammed into iPSCs compared with fibroblasts in mouse and human cells (Note S4). This motivated us to assess the reprogramming capacity of *in-vivo*-derived NSCs by using only ESM. As a result, we demonstrated iPSC generation from primary NSCs, which were derived from the biopsy of cerebral cortexes from two embryonic marmosets (Note S4).

Derivation of Transgene-free iPSCs from Canine Fibroblasts

Since we succeeded in the derivation of iNSLCs from human fibroblasts by the same method (Note S5). We sought to test the reprogramming method for fibroblasts of other non-rodent/non-primate mammalian species. Therefore, we attempted to reprogram ear skin-derived fibroblasts obtained from an adult dog (named K9) into iPSCs.

We transfected the EP-B vector set into the K9 fibroblasts, after which they were cultured in NSM following pre-expansion for 9 days (Figure 6A). As seen with the marmoset fibroblasts, primary dome-shaped colonies appeared by day 14 (Figure 6B, center), and putative iPSC colonies (termed canine iPSC [ciPSC]) emerged after culture in ESM (Figure 6B, right). Again, no colony could be derived from the transfected fibroblasts when ESM was used as the induction medium (n = 3), but colony formation was observed when using NSM for induction, although the derivation efficiency was relatively low (0–2 colonies derived from 1×10^6 transfected fibroblasts, n = 6). The mechanically isolated ciPSC clones grew immortally for over 20 passages.

Using RNA extracted from three ciPSC clones (K9 iPSC nos. 1–3), we performed RT-PCR using specific primers for endogenous PSC marker genes (*clfNANOG*, *clfOCT4*, and *clfSOX2*, Figure 6C). Results confirmed that the iPSCs endogenously expressed these PSC marker genes (Figure 6C). We also performed an mRNA-seq analysis of canine iPSCs (Note S6). By immunocytochemistry, we revealed that the ciPSCs were strongly positive for OCT4, SOX2, and AP (Figures 6D–6F). We further explored culture conditions which could enhance PSC marker expression in the ciPSCs (Note S7).

Moreover, we performed *in vitro* and *in vivo* three-germ layer differentiation assays using the K9 iPSC no. 1 clone, which resulted in successful differentiation into cells of all three germ layers (Figure 6G; Note S8). Following five passages after iPSC derivation, episomal vectors were confirmed to be removed from all three ciPSC clones (Figure 6H). Furthermore, we performed karyotyping of K9 iPSC nos. 1–3 clones, and found that 56% of K9 iPSC nos. 1–2 and 64% of K9 iPSC no. 3 retained normal chromosome number (2n = 78) (Figures 6I and 6J).

Thus, we demonstrated that our reprogramming method was applicable to the dog, which belongs to the taxonomic order *Carnivora*, distinct from marmosets and humans (in *Primate*).

Derivation of Transgene-free iPSCs from Porcine Fibroblasts

Next, we assessed whether our reprogramming method was applicable to ear skin-derived fibroblasts of a post-neonatal pig (named N01F). We transfected the EP-B vector set to the N01F fibroblasts. Using NSM, we observed the emergence of primary dome-shaped colonies with 0.028% ± 0.012% efficiency (n = 6; Figure 7A, left). After

(B) Hierarchical clustering analysis of marmoset samples based on the expression of pluripotency and ectoderm-related markers. We included data of marmoset ESCs (cjes001) and iPSCs (DPZcj_iPSC1), early-stage embryos (morula, early blastocyst, blastocyst, expand blastocyst, hatching blastocyst, hatched blastocyst, and epiblast_1–3_L1−2), and adult marmoset cortex (Adult_Cortex) were described previously (Debowski et al., 2015; Shiozawa et al., 2020; Yoshimatsu et al., 2019a).

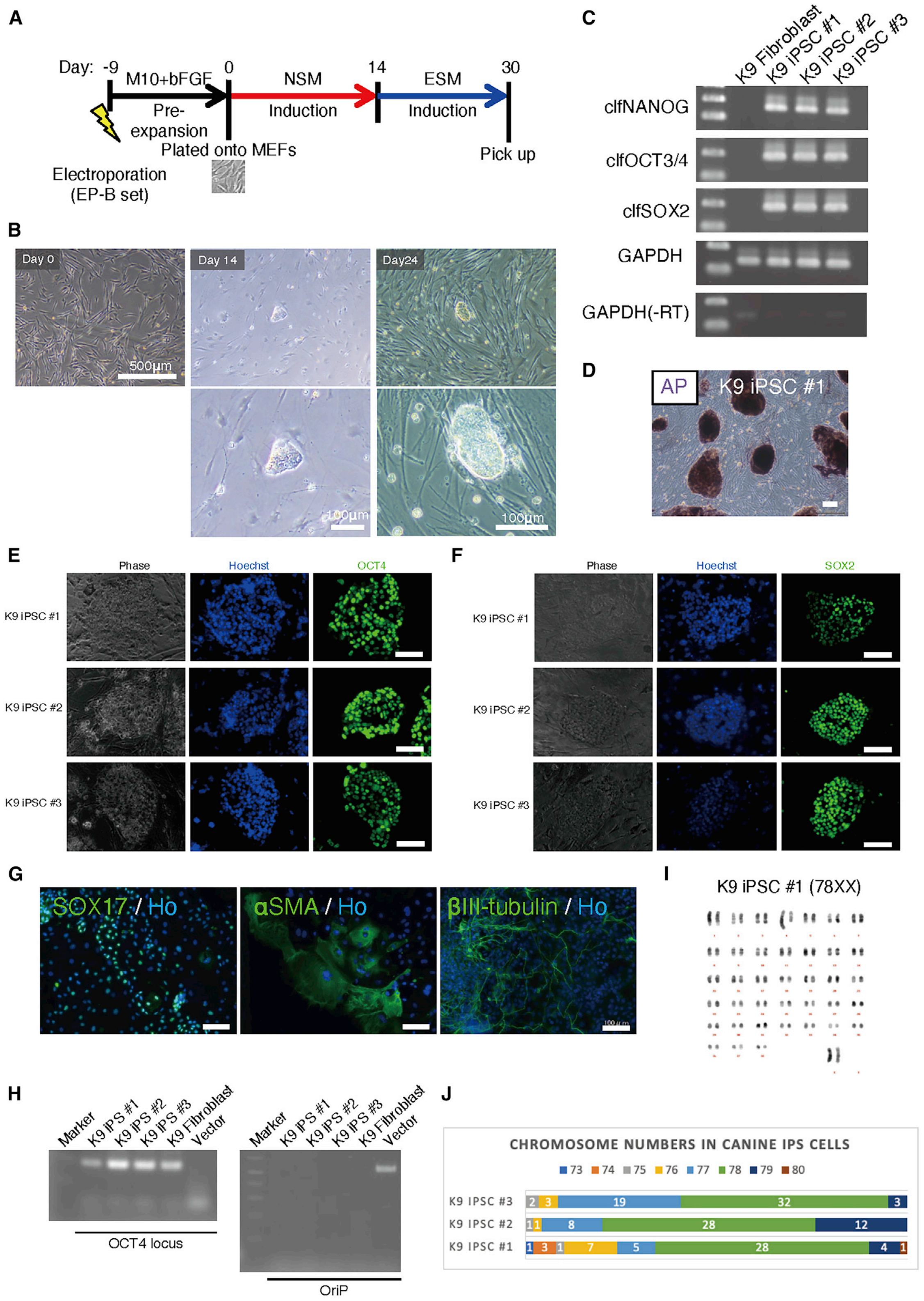

(legend on next page)

passaging these cells once (Figure 7A, center), we initially tested ESM for iPSC derivation. However, the attempt was unsuccessful since the majority of the cells remained dissociated in ESM and only few colony-like structures emerged. Next, we tested ESM supplemented with activin A (10 ng/mL) and transforming growth factor β1 (10 ng/mL), which are important factors for the maintenance of primed-state pluripotency (James et al., 2005; Nichols and Smith, 2009) and a WNT inhibitor IWP2, since WNT inhibition reportedly enabled the stable maintenance of pluripotency in flat-shaped colony-forming cells (Sumi et al., 2013; Wu et al., 2015). Following 2 weeks of culture using this medium, putative iPSC colonies (termed porcine iPSC [piPSC]) appeared (Figure 7A, right). These colonies were mechanically isolated and expanded for further analyses.

Using RNA extracted from two piPSC clones (N01F iPSC nos. 1–2), we confirmed endogenous expression of PSC markers (*ssOCT4*, *ssNANOG*, and *ssSOX2*) with specific primers for porcine sequences (Figure 7B), and other PSC and NSC marker expression in porcine iNSLCs and iPSCs was further assessed by mRNA-seq (Note S9). AP staining and immunocytochemical analyses showed that the piPSCs strongly expressed AP, OCT4, and SOX2 (Figures 7C–7E), while these cells were negative for NANOG, SSEA4, and TRA-1-60 (Note S10), as well as SSEA1, SSEA3, and TRA-1-81 (data not shown). As performed in ciPSCs (Note S7), we explored culture conditions which could enhance PSC marker expression in the piPSCs (see Note S10). In addition, after five passages, we confirmed the removal of episomal vectors by genomic PCR (Figure 7F). Furthermore, *in vitro* differentiation of one iPSC clone (N01F iPSC no. 1) resulted in successful differentiation of the cells into all three germ layers (Figure 7G). The karyotype of N01F iPSC no. 1 was highly stable, most of analyzed cells (49 out of 50) showed normal karyotype, 38XX (Figures 7H and 7I).

DISCUSSION

In this study, we generated transgene-free iPSCs from fibroblasts of multiple mammalian species. Using our reprogramming method, we were able to obtain transgene-free iPSCs from both embryonic and adult marmosets, an adult dog, and post-neonatal pigs. We also demonstrated that the resultant iPSCs were successfully differentiated into all three germ layers and germ cell linage. Thus, this method is robust and efficient, and applicable for reprogramming somatic fibroblasts from various mammalian species across different taxonomic orders into iPSCs.

The naive human and marmoset PSCs we recently reported (Kisa et al., 2017; Shiozawa et al., 2020) were characterized by the strong expression of *ESRRB*, which has an important role for the maintenance of the naive pluripotent state in murine ESCs (Festuccia et al., 2012). However, when utilizing the medium for inducing these naive human PSCs in this study, the primary dome-shaped colonies that initially appeared after the transfection of fibroblasts (Figures 1B, 1D, 6B, and 7A) were not naive or primed-state PSCs (Nichols and Smith, 2009), but were presumably NSC-like cells, as shown through multiple analyses.

Collectively, marmoset iNSLCs showed unique properties of gene expression and differentiation capacity similar to NSCs, but clearly distinct from marmoset PSCs. We discuss two rational possibilities that enabled us to obtain transgene-free iPSCs from somatic fibroblasts via an NSC-like state in this study (see Note S11).

The use of this method via an NSC-like state enabled the derivation of marmoset iPSCs completely free of transgenes, which has not been achieved in earlier reports (Debowski et al., 2015; Tomioka et al., 2010; Wu et al., 2010). More recently, we and other groups reported the generation of transgene-free marmoset iPSCs by episomal vectors

Figure 6. Derivation of Transgene-free iPSCs from Canine Fibroblasts
(A) Timetable for the derivation of ciPSCs.
(B) Representative images of transfected fibroblasts and primary colonies.
(C) RT-PCR analysis of PSC markers in K9 iPSC nos. 1–3 using primers specific for endogenous canine sequences. See Note S6 for further transcriptomic analyses of canine cells.
(D) AP staining of K9 iPSC no. 1. Scale bars, 500 μm.
(E and F) Immunocytochemical analysis of K9 iPSC no. 1 using primary antibodies of OCT4 and SOX2 (left and center). AP staining of K9 iPSC no. 1 (right). Scale bars, 100 μm. See Note S7 for further assessment of PSC marker immunoreactivity.
(G) Representative images of endodermal (SOX17-positive), mesodermal (αSMA-positive), and ectodermal (βIII tubulin-positive) cells differentiated from K9 iPSC no. 1 by *in vitro* differentiation assay (see Note S8 for *in vivo* differentiation assay). Scale bars, 100 μm.
(H) Genomic PCR analysis for the detection of residual episomal vectors using specific primers for the OriP sequence (Yu et al., 2009). Primers for the canine *OCT4* locus were used as an internal control.
(I) Q-banding-based karyotyping of a euploid cell from the K9 iPSC no. 1 line. High-resolution images of karyotyping are shown in Table S3.
(J) Chromosome counting of K9 iPSC nos. 1–3 by G-banding. Fifty cells were used in each cell line. Green bars show euploid (2n = 78) cells. Numerics on bars show the number of counted cells (in 50 cells) harboring each number of chromosomes.

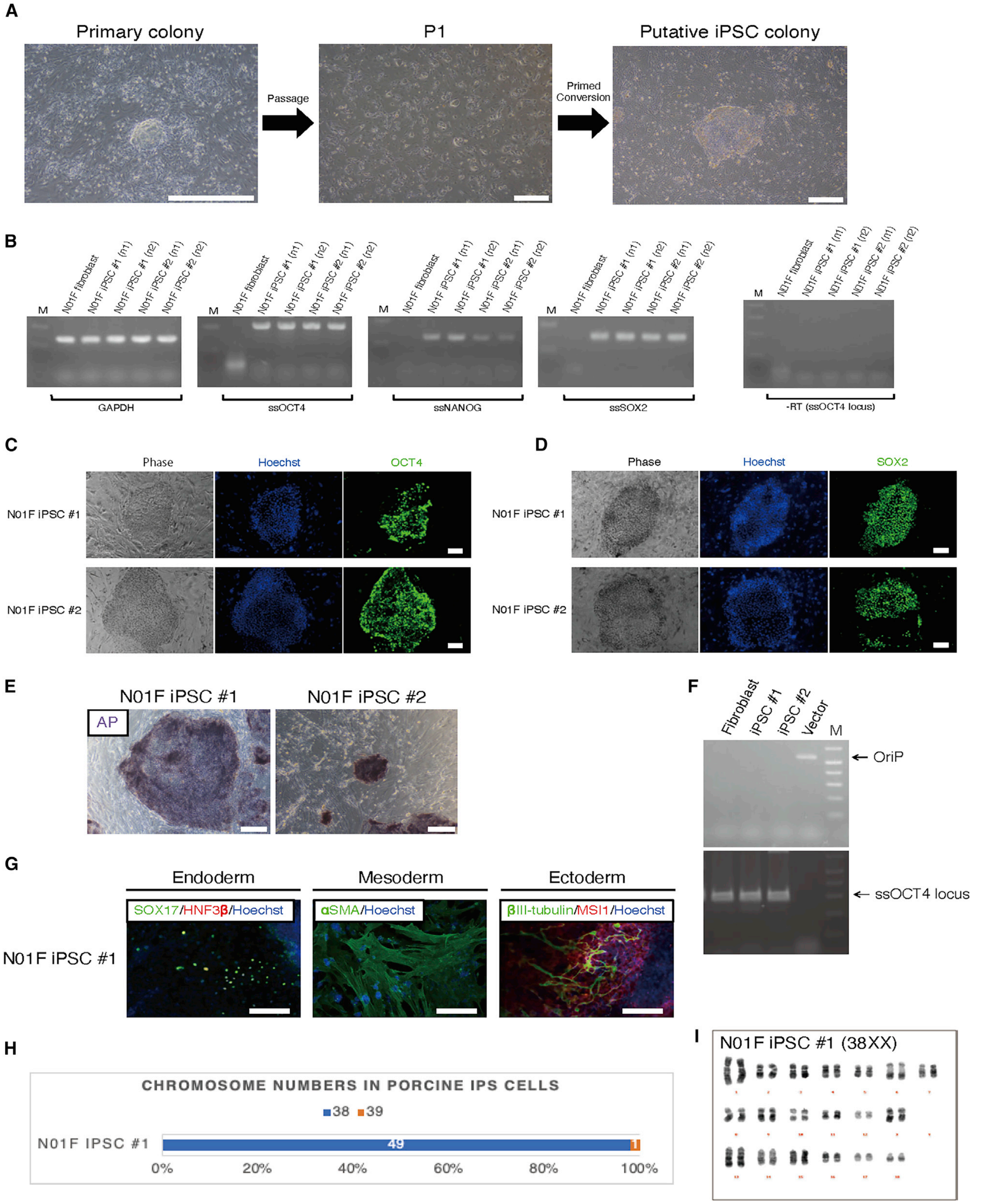

(legend on next page)

or RNA-based reprogramming, using chemical inhibitors similar to those supplemented in NSM (Nakajima et al., 2019; Petkov et al., 2020; Vermilyea et al., 2017; Watanabe et al., 2019), but the reprogramming mechanism has not been thoroughly investigated. This study suggests that passing through this NSC-like state facilitates iPSC reprogramming for marmoset fibroblasts. In addition, in sharp contrast to the previous reports, our study confirmed the efficacy of the iNSLC-mediated reprogramming of somatic fibroblasts in species besides the marmoset. In particular, successful derivation of transgene-free ciPSCs and piPSCs is significant, due to the species-specific difficulties in previous studies (see Note S12).

The definition of bona fide iPSCs remains controversial. Tetraploid complementation is the most stringent criterion for evaluating the developmental potential of murine iPSCs (Wernig et al., 2007). Less stringently and more practically, the potential for germline-transmitting chimera formation through blastocyst injection has also been used as a developmental criterion for murine iPSCs (Hamanaka et al., 2011; Okita et al., 2007). However, as there are few reports of non-rodent mammalian iPSCs that are germline-competent, except for one on piPSCs (West et al., 2011), and none on primates, including humans, there is a need for an alternative criterion for these species. In this context, several studies reported that transgene excision in iPSCs seemed crucial for normal development *in vivo* (Du et al., 2015; Okita et al., 2007; West et al., 2011), and transgene-excised hiPSCs have been suggested to be "safer" than transgene-integrated ones, as the reactivation of transgenes can increase tumorigenic risk (Galat et al., 2016). Thus, we propose the absence of transgene(s) to be a tentative criterion for bona fide non-rodent mammalian iPSCs.

In conclusion, we obtained transgene-free iPSCs fulfilling the criterion above in three species, spanning various taxonomic orders. Our method described in this study may facilitate the reprogramming process in the class *Mammalia*.

EXPERIMENTAL PROCEDURES

Animals and Ethical Statements

All animal experiments were performed in accordance with the guidelines for laboratory animals by the National Institutes of Health, and the Ministry of Education, Culture, Sports, Science and Technology (MEXT) of Japan, and were approved by the institutional Animal Care and Use Committee of Keio University, Nihon University, and RIKEN (approval no. H27-2-306(4)).

Animal care was conducted in accordance with the National Research Council (NRC) Guide for the Care and Use of Laboratory Animals (2011).

Other experimental procedures, including information of animals, cell culture, genomic and transcriptomic analyses, are described in Supplemental Experimental Procedures.

SUPPLEMENTAL INFORMATION

Supplemental information can be found online at https://doi.org/10.1016/j.stemcr.2021.03.002.

AUTHOR CONTRIBUTIONS

Conceptualization, S.Y.; Methodology, S.Y.; Software, S.Y., M. Nakajima, T. Sanosaka, and K.I.; Validation and Format Analysis, S.Y., M. Nakajima, A.I., and T. Sato; Investigation and Resources, S.Y., A.I., T. Sanosaka, R.N., M.I., H.W., J.O., Y.T., E.A., E.S., R.B., T.N., K.E., and S.S.; Data Curation, S.Y., M. Nakajima, and T.Sanosaka; Writing – Original Draft, S.Y.; Writing – Review & Editing, S.Y., M. Nakajima, T. Sanosaka, M. Nakamura, T.N., and H.O.; Supervision and Project Administration, H.O.; Funding Acquisition, H.O., K.E., and S.Y.

DECLARATION OF INTERESTS

H.O. serves as a paid scientific advisor at SanBio Co. Ltd. and K Pharma Inc., but these companies had no control over this work. The other authors declare neither financial nor non-financial competing interests.

ACKNOWLEDGMENTS

We especially thank Drs. Takashi Sasaki, Jun Kohyama, Ayako Y. Murayama, and Ms. Kanae Ohtsu (at Keio University), Drs.

Figure 7. Derivation of Transgene-free iPSCs from Porcine Fibroblasts

(A) Representative images of primary colonies (left), passaged cells (center), and putative iPSC colonies (right). Scale bars, 500 μm.

(B) RT-PCR analysis of PSC marker genes (*OCT4*, *NANOG*, and *SOX2*) in N01F iPSC nos. 1–2 (n = 2, independent experiments) using primers specific for endogenous porcine sequences. See Note S9 for further transcriptomic analyses of porcine cells.

(D and E) Immunocytochemical staining of N01F iPSC nos. 1–2 using primary antibodies of PSC markers. Scale bars, 100 μm. See Note S10 for further assessment of PSC marker immunoreactivity. (E) AP staining of N01F iPSC nos. 1–2. Scale bars, 200 μm.

(F) Genomic PCR analysis for the detection of residual episomal vectors using specific primers for the OriP sequence (Yu et al., 2009). Primers for the porcine *OCT4* (*ssOCT4*) locus were used as an internal control.

(G) Representative images of endodermal (SOX17, HNF3β-positive), mesodermal (αSMA-positive) and ectodermal (βIII-tubulin, MSI1-positive) cells differentiated from N01F iPSC no. 1 by *in vitro* differentiation assay. Scale bars, 100 μm.

(H) Chromosome counting of N01F iPSC no. 1 by G-banding. Fifty cells were used in each cell line. Blue bars show euploid (2n = 38) cells. Numerics on bars show the number of counted cells (in 50 cells) harboring each number of chromosomes.

(I) Q-banding-based karyotyping of a euploid cell from the N01F iPSC no. 1 line. High-resolution images of karyotyping are shown in Table S3.

Hirotaka Kondo and Hisashi Shibuya (at Nihon University). We also thank all the laboratory members of H.O. and K.E. for their encouragement and generous support for the current study. This study was performed under the Strategic Research Program for Brain Sciences and Brain Mapping by Integrated Neurotechnologies for Disease Studies (Brain/MINDS) of the the AMED (JP20dm0207001 to H.O.), KAKENHI 19J12871 and 20K22660 (to S.Y.), 20H03156 (to K.E.), and internal budgets from Keio University, including the Program for the Advancement of Research in Core Projects on Longevity of the Keio University Global Research Institute from Keio University (to H.O.).

Received: April 21, 2020
Revised: February 24, 2021
Accepted: March 2, 2021
Published: April 1, 2021

REFERENCES

Ben-Nun, I.F., Montague, S.C., Houck, M.L., Tran, H.T., Garitaonandia, I., Leonardo, T.R., Wang, Y.C., Charter, S.J., Laurent, L.C., Ryder, O.A., et al. (2011). Induced pluripotent stem cells from highly endangered species. Nat. Methods *8*, 829–831.

Bradley, A., Evans, M., Kaufman, M.H., and Robertson, E. (1984). Formation of germ-line chimaeras from embryo-derived teratocarcinoma cell lines. Nature *309*, 255–256.

Cordova, A., King, W.A., and Mastromonaco, G.F. (2017). Choosing a culture medium for SCNT and iSCNT reconstructed embryos: from domestic to wildlife species. J. Anim. Sci. Technol. *59*, 24.

Debowski, K., Warthemann, R., Lentes, J., Salinas-Riester, G., Dressel, R., Langenstroth, D., Gromoll, J., Sasaki, E., and Behr, R. (2015). Non-viral generation of marmoset monkey iPS cells by a six-factor-in-one-vector approach. PLoS One *10*, e0118424.

Du, X., Feng, T., Yu, D., Wu, Y., Zou, H., Ma, S., Feng, C., Huang, Y., Ouyang, H., Hu, X., et al. (2015). Barriers for deriving transgene-free pig iPS cells with episomal vectors. Stem Cells *33*, 3228–3238.

Evans, M.J., and Kaufman, M.H. (1981). Establishment in culture of pluripotential cells from mouse embryos. Nature *292*, 154–156.

Festuccia, N., Osorno, R., Halbritter, F., Karwacki-Neisius, V., Navarro, P., Colby, D., Wong, F., Yates, A., Tomlinson, S.R., and Chambers, I. (2012). Esrrb is a direct Nanog target gene that can substitute for Nanog function in pluripotent cells. Cell Stem Cell *11*, 477–490.

Fujimori, K., Ishikawa, M., Otomo, A., Atsuta, N., Nakamura, R., Akiyama, T., Hadano, S., Aoki, M., Saya, H., Sobue, G., et al. (2018). Modeling sporadic ALS in iPSC-derived motor neurons identifies a potential therapeutic agent. Nat. Med. *24*, 1579–1589.

Galat, V., Galat, Y., Perepitchka, M., Jennings, L.J., Iannaccone, P.M., and Hendrix, M.J. (2016). Transgene reactivation in induced pluripotent stem cell derivatives and reversion to pluripotency of induced pluripotent stem cell-derived mesenchymal stem cells. Stem Cells Dev. *25*, 1060–1072.

Hamanaka, S., Yamaguchi, T., Kobayashi, T., Kato-Itoh, M., Yamazaki, S., Sato, H., Umino, A., Wakiyama, Y., Arai, M., Sanbo, M., et al. (2011). Generation of germline-competent rat induced pluripotent stem cells. PLoS One *6*, e22008.

Hayashi, K., Ogushi, S., Kurimoto, K., Shimamoto, S., Ohta, H., and Saitou, M. (2012). Offspring from oocytes derived from in vitro primordial germ cell-like cells in mice. Science *338*, 971–975.

Hayashi, K., Ohta, H., Kurimoto, K., Aramaki, S., and Saitou, M. (2011). Reconstitution of the mouse germ cell specification pathway in culture by pluripotent stem cells. Cell *146*, 519–532.

Hayashi, T., Ozaki, H., Sasagawa, Y., Umeda, M., Danno, H., and Nikaido, I. (2018). Single-cell full-length total RNA sequencing uncovers dynamics of recursive splicing and enhancer RNAs. Nat. Commun. *9*, 619.

Honda, A., Choijookhuu, N., Izu, H., Kawano, Y., Inokuchi, M., Honsho, K., Lee, A.R., Nabekura, H., Ohta, H., Tsukiyama, T., et al. (2017). Flexible adaptation of male germ cells from female iPSCs of endangered *Tokudaia osimensis*. Sci. Adv. *3*, e1602179.

James, D., Levine, A.J., Besser, D., and Hemmati-Brivanlou, A. (2005). TGFbeta/activin/nodal signaling is necessary for the maintenance of pluripotency in human embryonic stem cells. Development *132*, 1273–1282.

Kamiya, D., Banno, S., Sasai, N., Ohgushi, M., Inomata, H., Watanabe, K., Kawada, M., Yakura, R., Kiyonari, H., Nakao, K., et al. (2011). Intrinsic transition of embryonic stem-cell differentiation into neural progenitors. Nature *470*, 503–509.

Kisa, F., Shiozawa, S., Oda, K., Yoshimatsu, S., Nakamura, M., Koya, I., Kawai, K., Suzuki, S., and Okano, H. (2017). Naive-like ESRRB(+) iPSCs with the capacity for rapid neural differentiation. Stem Cell Rep. *9*, 1825–1838.

Kobayashi, T., Zhang, H., Tang, W.W.C., Irie, N., Withey, S., Klisch, D., Sybirna, A., Dietmann, S., Contreras, D.A., Webb, R., et al. (2017). Principles of early human development and germ cell program from conserved model systems. Nature *546*, 416–420.

Li, S., Lan, H., Men, H., Wu, Y., Li, N., Capecchi, M.R., Bryda, E.C., and Wu, S. (2017). Derivation of transgene-free rat induced pluripotent stem cells approximating the quality of embryonic stem cells. Stem Cells Transl. Med. *6*, 340–351.

Liu, Z., Cai, Y., Wang, Y., Nie, Y., Zhang, C., Xu, Y., Zhang, X., Lu, Y., Wang, Z., Poo, M., et al. (2018). Cloning of macaque monkeys by somatic cell nuclear transfer. Cell *172*, 881–887 e887.

Maekawa, M., Yamaguchi, K., Nakamura, T., Shibukawa, R., Kodanaka, I., Ichisaka, T., Kawamura, Y., Mochizuki, H., Goshima, N., and Yamanaka, S. (2011). Direct reprogramming of somatic cells is promoted by maternal transcription factor Glis1. Nature *474*, 225–229.

Maherali, N., Sridharan, R., Xie, W., Utikal, J., Eminli, S., Arnold, K., Stadtfeld, M., Yachechko, R., Tchieu, J., Jaenisch, R., et al. (2007). Directly reprogrammed fibroblasts show global epigenetic remodeling and widespread tissue contribution. Cell Stem Cell *1*, 55–70.

Marchetto, M.C.N., Narvaiza, I., Denli, A.M., Benner, C., Lazzarini, T.A., Nathanson, J.L., Paquola, A.C.M., Desai, K.N., Herai, R.H., Weitzman, M.D., et al. (2013). Differential L1 regulation in pluripotent stem cells of humans and apes. Nature *503*, 525–529.

Martin, G.R. (1981). Isolation of a pluripotent cell line from early mouse embryos cultured in medium conditioned by teratocarcinoma stem cells. Proc. Natl. Acad. Sci. U S A *78*, 7634–7638.

Matoba, S., Liu, Y., Lu, F., Iwabuchi, K.A., Shen, L., Inoue, A., and Zhang, Y. (2014). Embryonic development following somatic cell nuclear transfer impeded by persisting histone methylation. Cell *159*, 884–895.

Nair, V. (2008). Retrovirus-induced oncogenesis and safety of retroviral vectors. Curr. Opin. Mol. Ther. *10*, 431–438.

Nakajima, M., Yoshimatsu, S., Sato, T., Nakamura, M., Okahara, J., Sasaki, E., Shiozawa, S., and Okano, H. (2019). Establishment of induced pluripotent stem cells from common marmoset fibroblasts by RNA-based reprogramming. Biochem. Biophys. Res. Commun. *515*, 593–599.

Nichols, J., and Smith, A. (2009). Naive and primed pluripotent states. Cell Stem Cell *4*, 487–492.

Nii, T., Marumoto, T., Kawano, H., Yamaguchi, S., Liao, J., Okada, M., Sasaki, E., Miura, Y., and Tani, K. (2014). Analysis of essential pathways for self-renewal in common marmoset embryonic stem cells. FEBS Open Bio *4*, 213–219.

Ogorevc, J., Orehek, S., and Dovc, P. (2016). Cellular reprogramming in farm animals: an overview of iPSC generation in the mammalian farm animal species. J. Anim. Sci. Biotechnol. *7*, 10.

Okano, H., and Yamanaka, S. (2014). iPS cell technologies: significance and applications to CNS regeneration and disease. Mol. Brain *7*, 22.

Okita, K., Ichisaka, T., and Yamanaka, S. (2007). Generation of germline-competent induced pluripotent stem cells. Nature *448*, 313–317.

Okita, K., Matsumura, Y., Sato, Y., Okada, A., Morizane, A., Okamoto, S., Hong, H., Nakagawa, M., Tanabe, K., Tezuka, K., et al. (2011). A more efficient method to generate integration-free human iPS cells. Nat. Methods *8*, 409–412.

Okita, K., Yamakawa, T., Matsumura, Y., Sato, Y., Amano, N., Watanabe, A., Goshima, N., and Yamanaka, S. (2013). An efficient nonviral method to generate integration-free human-induced pluripotent stem cells from cord blood and peripheral blood cells. Stem Cells *31*, 458–466.

Petkov, S., Dressel, R., Rodriguez-Polo, I., and Behr, R. (2020). Controlling the switch from neurogenesis to pluripotency during marmoset monkey somatic cell reprogramming with self-replicating mRNAs and small molecules. Cells *9*, 2422.

Sakakibara, S., Imai, T., Hamaguchi, K., Okabe, M., Aruga, J., Nakajima, K., Yasutomi, D., Nagata, T., Kurihara, Y., Uesugi, S., et al. (1996). Mouse-Musashi-1, a neural RNA-binding protein highly enriched in the mammalian CNS stem cell. Dev. Biol. *176*, 230–242.

Sansom, S.N., Griffiths, D.S., Faedo, A., Kleinjan, D.J., Ruan, Y., Smith, J., van Heyningen, V., Rubenstein, J.L., and Livesey, F.J. (2009). The level of the transcription factor Pax6 is essential for controlling the balance between neural stem cell self-renewal and neurogenesis. PLoS Genet. *5*, e1000511.

Sasaki, E., Hanazawa, K., Kurita, R., Akatsuka, A., Yoshizaki, T., Ishii, H., Tanioka, Y., Ohnishi, Y., Suemizu, H., Sugawara, A., et al. (2005). Establishment of novel embryonic stem cell lines derived from the common marmoset (*Callithrix jacchus*). Stem Cells *23*, 1304–1313.

Shiozawa, S., Nakajima, M., Okahara, J., Kuortaki, Y., Kisa, F., Yoshimatsu, S., Nakamura, M., Koya, I., Yoshimura, M., Sasagawa, Y., et al. (2020). Primed to naive-like conversion of the common marmoset embryonic stem cells. Stem Cells Dev. *29*, 761–773.

Smith, A.G. (2001). Embryo-derived stem cells: of mice and men. Annu. Rev. Cell Dev. Biol. *17*, 435–462.

Sumi, T., Oki, S., Kitajima, K., and Meno, C. (2013). Epiblast ground state is controlled by canonical Wnt/beta-catenin signaling in the postimplantation mouse embryo and epiblast stem cells. PLoS One *8*, e63378.

Takahashi, K., Tanabe, K., Ohnuki, M., Narita, M., Ichisaka, T., Tomoda, K., and Yamanaka, S. (2007). Induction of pluripotent stem cells from adult human fibroblasts by defined factors. Cell *131*, 861–872.

Takahashi, K., and Yamanaka, S. (2006). Induction of pluripotent stem cells from mouse embryonic and adult fibroblast cultures by defined factors. Cell *126*, 663–676.

Thomas, K.R., and Capecchi, M.R. (1987). Site-directed mutagenesis by gene targeting in mouse embryo-derived stem cells. Cell *51*, 503–512.

Thomson, J.A., Itskovitz-Eldor, J., Shapiro, S.S., Waknitz, M.A., Swiergiel, J.J., Marshall, V.S., and Jones, J.M. (1998). Embryonic stem cell lines derived from human blastocysts. Science *282*, 1145–1147.

Thomson, J.A., Kalishman, J., Golos, T.G., Durning, M., Harris, C.P., Becker, R.A., and Hearn, J.P. (1995). Isolation of a primate embryonic stem cell line. Proc. Natl. Acad. Sci. U S A *92*, 7844–7848.

Tomioka, I., Maeda, T., Shimada, H., Kawai, K., Okada, Y., Igarashi, H., Oiwa, R., Iwasaki, T., Aoki, M., Kimura, T., et al. (2010). Generating induced pluripotent stem cells from common marmoset (*Callithrix jacchus*) fetal liver cells using defined factors, including Lin28. Genes Cells *15*, 959–969.

Vermilyea, S.C., Guthrie, S., Meyer, M., Smuga-Otto, K., Braun, K., Howden, S., Thomson, J.A., Zhang, S.C., Emborg, M.E., and Golos, T.G. (2017). Induced pluripotent stem cell-derived dopaminergic neurons from adult common marmoset fibroblasts. Stem Cells Dev. *26*, 1225–1235.

Watanabe, T., Yamazaki, S., Yoneda, N., Shinohara, H., Tomioka, I., Higuchi, Y., Yagoto, M., Ema, M., Suemizu, H., Kawai, K., et al. (2019). Highly efficient induction of primate iPS cells by combining RNA transfection and chemical compounds. Genes Cells *24*, 473–484.

Wernig, M., Lengner, C.J., Hanna, J., Lodato, M.A., Steine, E., Foreman, R., Staerk, J., Markoulaki, S., and Jaenisch, R. (2008). A drug-inducible transgenic system for direct reprogramming of multiple somatic cell types. Nat. Biotechnol. *26*, 916–924.

Wernig, M., Meissner, A., Foreman, R., Brambrink, T., Ku, M., Hochedlinger, K., Bernstein, B.E., and Jaenisch, R. (2007). In vitro reprogramming of fibroblasts into a pluripotent ES-cell-like state. Nature *448*, 318–324.

West, F.D., Uhl, E.W., Liu, Y., Stowe, H., Lu, Y., Yu, P., Gallegos-Cardenas, A., Pratt, S.L., and Stice, S.L. (2011). Brief report: chimeric pigs produced from induced pluripotent stem cells demonstrate germline transmission and no evidence of tumor formation in young pigs. Stem Cells *29*, 1640–1643.

Wu, J., Okamura, D., Li, M., Suzuki, K., Luo, C., Ma, L., He, Y., Li, Z., Benner, C., Tamura, I., et al. (2015). An alternative pluripotent state confers interspecies chimaeric competency. Nature *521*, 316–321.

Wu, S., Wu, Y., Zhang, X., and Capecchi, M.R. (2014). Efficient germ-line transmission obtained with transgene-free induced pluripotent stem cells. Proc. Natl. Acad. Sci. U S A *111*, 10678–10683.

Wu, Y., Zhang, Y., Mishra, A., Tardif, S.D., and Hornsby, P.J. (2010). Generation of induced pluripotent stem cells from newborn marmoset skin fibroblasts. Stem Cell Res. *4*, 180–188.

Yamanaka, S. (2012). Induced pluripotent stem cells: past, present, and future. Cell Stem Cell *10*, 678–684.

Yoshimatsu, S., Nakamura, M., Nakajima, M., Nemoto, A., Sato, T., Sasaki, E., Shiozawa, S., and Okano, H. (2019a). Evaluating the efficacy of small molecules for neural differentiation of common marmoset ESCs and iPSCs. Neurosci. Res. *155*, 1–11.

Yoshimatsu, S., Sato, T., Yamamoto, M., Sasaki, E., Nakajima, M., Nakamura, M., Shiozawa, S., Noce, T., and Okano, H. (2020). Generation of a male common marmoset embryonic stem cell line DSY127-BV8VT1 carrying double reporters specific for the germ cell linage using the CRISPR-Cas9 and PiggyBac transposase systems. Stem Cell Res. *44*, 101740.

Yoshimatsu, S., Sone, T., Nakajima, M., Sato, T., Okochi, R., Ishikawa, M., Nakamura, M., Sasaki, E., Shiozawa, S., and Okano, H. (2019b). A versatile toolbox for knock-in gene targeting based on the Multisite Gateway technology. PLoS One *14*, e0221164.

Yu, J., Hu, K., Smuga-Otto, K., Tian, S., Stewart, R., Slukvin, I.I., and Thomson, J.A. (2009). Human induced pluripotent stem cells free of vector and transgene sequences. Science *324*, 797–801.

Yu, J., Vodyanik, M.A., Smuga-Otto, K., Antosiewicz-Bourget, J., Frane, J.L., Tian, S., Nie, J., Jonsdottir, G.A., Ruotti, V., Stewart, R., et al. (2007). Induced pluripotent stem cell lines derived from human somatic cells. Science *318*, 1917–1920.

Stem Cell Reports, Volume *16*

Supplemental Information

Non-viral Induction of Transgene-free iPSCs from Somatic Fibroblasts of Multiple Mammalian Species

Sho Yoshimatsu, Mayutaka Nakajima, Aozora Iguchi, Tsukasa Sanosaka, Tsukika Sato, Mari Nakamura, Ryusuke Nakajima, Eri Arai, Mitsuru Ishikawa, Kent Imaizumi, Hirotaka Watanabe, Junko Okahara, Toshiaki Noce, Yuta Takeda, Erika Sasaki, Rüdiger Behr, Kazuya Edamura, Seiji Shiozawa, and Hideyuki Okano

Supplemental Notes

Note S1. Induction of marmoset primordial germ cell-like cells (PGCLCs) (related to Figure S5).

For PGCLC induction, since our preliminary attempts with only cytokines (BMP4, SCF, EGF and LIF) were unsuccessful, we exploited the combinatorial usage of the dexamethasone/doxycycline-inducible *SOX17/BLIMP1* overexpression system and cytokines (BMP4, SCF, EGF and LIF) (Figure S5C−D) (Supplementary Methods) according to the reported methods in previous studies on PGCLC induction from human and porcine PSCs (Gao et al., 2019; Kobayashi et al., 2017).

However, compared to the BLIMP1-Venus-positive rates of Day10-differentiated cells from marmoset ESCs (Figure S5E−F; ~50% from No. 40 ESCs and ~40% from DSY127 ESCs), the BLIMP1-Venus-positive rate of Day10-differentiated cells from marmoset iPSC (E01F A-2-2) was extremely low (Figure S5 E−F; 1−2%). Nevertheless, by mRNA-seq analysis, we discovered the PGCLCs (BLIMP1-Venus-positive cells on Day 10) from both of marmoset ESCs and iPSCs were plotted together in PCA (Figure S5G).

Compared to marmoset ESCs and iPSCs, the resultant PGCLCs showed enhanced expression of PGC specification markers, including *SOX17*, *PRDM1* (*BLIMP1*), *NANOS3*, *SOX15*, *KIT*, *ALPL* and *TFAP2C* (Figure S5H−I). In addition, the marmoset PGCLCs showed sustained expression of pluripotency-related genes such as *POU5F1* (*OCT4*), *UTF1*, *NANOG*, and *LIN28A*, while *SOX2* expression was significantly decreased (Figure S5H−I), which is consistent with previous findings on primate PGCs and PGCLCs (Irie et al., 2015; Sasaki et al., 2016; Sasaki et al., 2015), but clearly distinct from mouse PGCs (Campolo et al., 2013; Ohinata et al., 2009).

Moreover, by differential expressed gene (DEG) analysis of PGCLCs and PSCs (Figure S5I and Supplemental Table 1), we discovered significantly upregulated cell-surface markers including *KIT* (CD117) and *CXCR4* (CD184), which may make it possible to monitor marmoset PGCLC differentiation without the usage of genetic reporters. RNA from a whole testis of an adult marmoset (Adult_Testis) was used as a positive control for mature germ cell markers, including *DDX4*, *DAZL*, *PIWIL1*, *PIWIL4*, *SYCP3* and *SYCP1* (Figure S5I). Although DSY127 ESC/E01F iPSC-derived PGCLCs showed a marginal increase in the expression of *DDX4*, other markers were not upregulated in all PGCLC samples (Figure S5I).

Note S2. The high neurogenic potential of iNSLCs (related to Figure S6).

To analyze the neurogenic potential of the iNSLCs, we performed a direct neurosphere formation assay (Yoshimatsu et al., 2019a) using the MHM medium, which supports neuronal survival without restricting differentiation toward other cell lineages. The marmoset iNSLCs, iPSCs and ESCs were cultured in suspension for one week, followed by further differentiation in an adherent culture (Figure S6G). βIII-tubulin(+) neurons were not derived from the ESCs and iPSCs using this method (Figure S6H), which was consistent with previous observations (Yoshimatsu et al., 2019a). Surprisingly, under the same condition, βIII-tubulin(+) neurons were derived from iNSLCs with 14−78% efficiency (Figure S6H−I). The iNSLCs showed various degrees of cell viability and neuronal differentiation efficiency following differentiation, which may be due to the differential ectopic expression levels of the remaining transgenes (Figure 4F). Among the iNSLCs, I5061F B-0 iNSLCs showed the highest efficiency of neuronal differentiation, which we decided to use for further differentiation assays.

To investigate the gliogenic potential of the iNSLC, day 7 primary neurospheres were dissociated into single cells and cultured in suspension for an additional week for the formation of secondary neurospheres, which was followed by further differentiation in an adherent culture. As a result, we observed ~ 30% GFAP-positive glial cells among the total population of cells (Figure S6J).

It was previously reported that the regional identity of induced neural progenitors derived from primate fibroblasts by ectopic expression of *OCT4*, *SOX2*, *KLF4* and *C-MYC* can be controlled by the supplementation of morphogens that are critical for neural development (Lu et al., 2013). When testing various morphogens, we succeeded in the posteriorization/ventralization of the iNSLCs by supplementation of retinoic acid, WNT agonist CHIR99021, and SHH agonist Purmorphamine to the medium during secondary neurosphere formation, which resulted in the emergence of ChAT/Islet1-positive motor neurons (~ 20% double-positive neurons out of the total population of cells; Figure S6K), and ~ 1% Galactocerebroside (GalC)-positive oligodendrocytes (Figure S6L).

Note S3. Microarray analysis of marmoset fibroblasts, iNSLCs, ESCs and iPSCs.

To elucidate the global differences and similarities of gene expression among these cells, we performed transcriptomic analyses by 3'IVT microarray. We used total RNA extracted

from the following six samples: embryonic marmoset fibroblasts (E01F fibroblasts), embryonic marmoset iPSCs (E01F A-2-2 iPSCs), embryonic marmoset iNSLCs (E01F A-2 iNSLCs), adult marmoset iPSCs (I5061F B-3-3 iPSCs), adult marmoset iNSLCs (I5061F B-3 iNSLCs) and marmoset ESCs (No.40 ESCs). The iNSLC clones, E01F A-2 and I5061 B-3, were parental of the iPSC clones, E01F A-2-2 and I5061F B-3-3, respectively.

By hierarchical clustering of global gene expression as shown below, we found that the PSCs (ESCs and iPSCs), fibroblasts, and iNSLCs were clearly distinguished from each other.

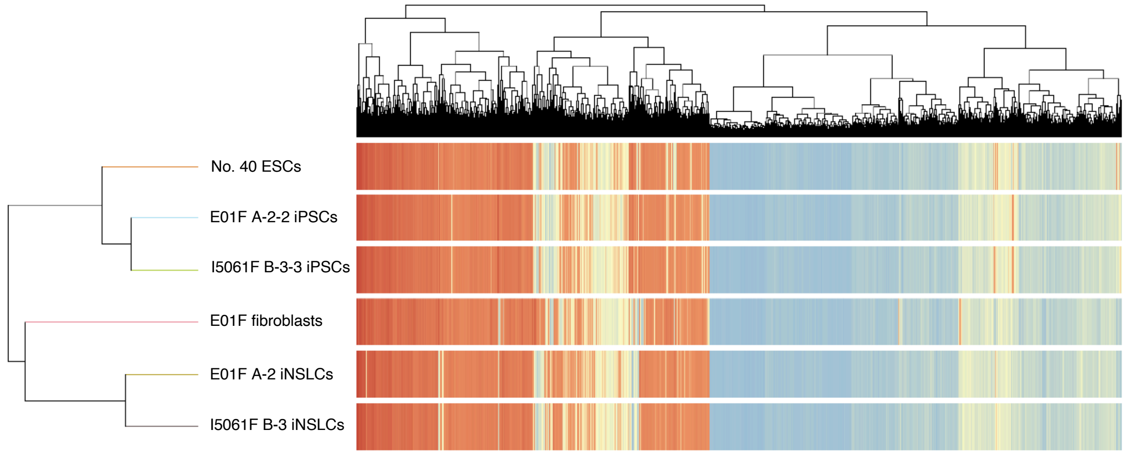

In addition, when looking at individual genes (shown below), we detected high expression of NSC markers *PAX6, ASCL1, MSI and MSI2* in the iNSLCs, which correlated with the results of the qPCR analysis (Figure 4G). Meanwhile, PSC markers *DPPA2, POU5F1* (*OCT4*), *UTF1, RARG*, *DPPA5, NANOG* and *GDF3* were specifically expressed in the PSCs, but not in the iNSLCs (Figure S7B). Notably, *SALL4* was strongly expressed in both iNSLCs and PSCs, but not in the fibroblasts (Figure S7B). Hou *et al.* reported that during a chemical compound-based reprogramming of murine fibroblasts toward pluripotency, *SALL4* expression was upregulated along with pluripotency-related marker genes at the initial stages of the reprogramming process (Hou et al., 2013). Importantly, some of these compounds (Forskolin, CHIR99021 and 616452 (TGF-β inhibitor)) were also supplemented in the NSM of our study, which may be why *SALL4* was upregulated in the iNSLCs. On the other hand, we did not detect any *SALL4* expression in the *in vivo*-derived NSCs (data not shown). Thus, the high expression of *SALL4* in the iNSLCs may suggest that the iNSLC state reflects an early phase of the reprogramming process toward pluripotency.

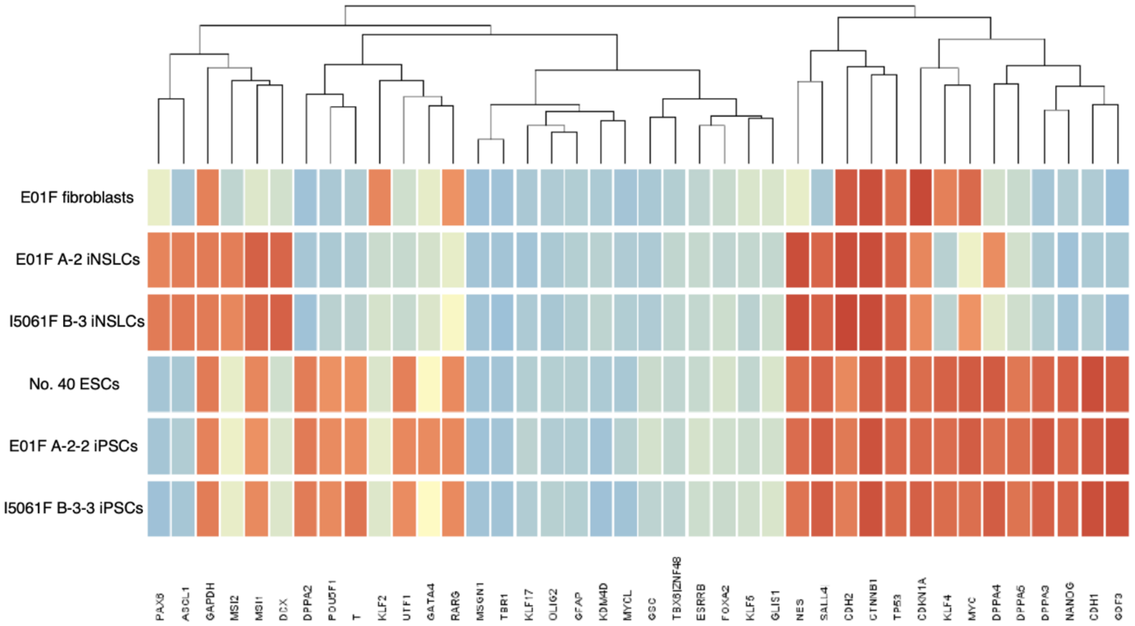

Furthermore, by principal component analysis (PCA) of the 6 types of cells (shown below), fibroblasts and the other 5 types of cells were clearly separated by component 1 (95.45%). Additionally, iNSLCs and PSCs were clearly separated by component 2 (2.17%).

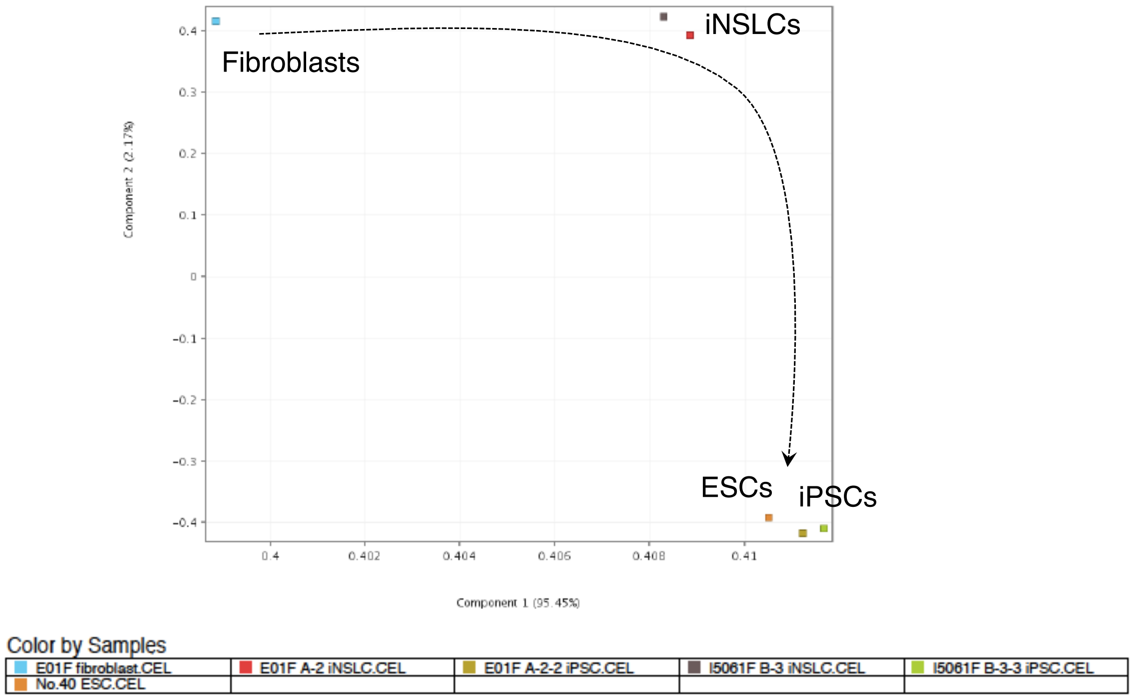

Note S4. Reprogramming of marmoset *in vivo*-derived NSCs toward a pluripotent state.

To investigate an iPSC-reprogramming potential of marmoset NSCs, we used primary

NSCs derived from the biopsy of cerebral cortexes from two embryonic marmosets (named CTXNS1 and CTXNS2). We transfected the EP-B vector set (Figure 1A, bottom) into the NSCs, pre-expanded them in MHM medium for four days (as shown below right, white scale bar = 200 μm), and plated them onto MEFs using ESM.

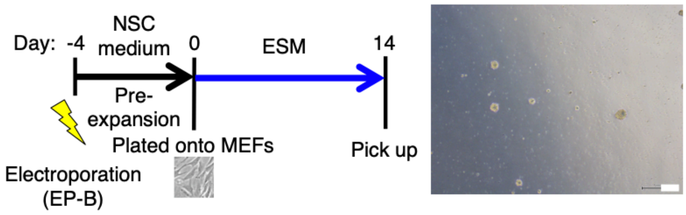

Surprisingly, although we could not induce colony formation from marmoset fibroblasts with ESM, we succeeded in deriving putative iPSC colonies (a representative colony on day 13 is shown below, scale bar = 200 μm) from the NSCs at an efficiency of 0.055 ± 0.012% (n = 2, independent experiments using NSCs from two marmosets).

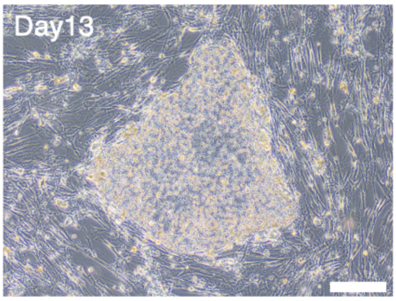

Using one putative iPSC clone from each marmoset (CTXNS1 B-1 and CTXNS2 B-1), we confirmed pluripotency marker expression (scale bars = 100 μm) as shown below.
In addition, we confirmed the three-germ-layer differentiation potential of the NSC-derived iPSCs (scale bars = 100 μm) as shown below.

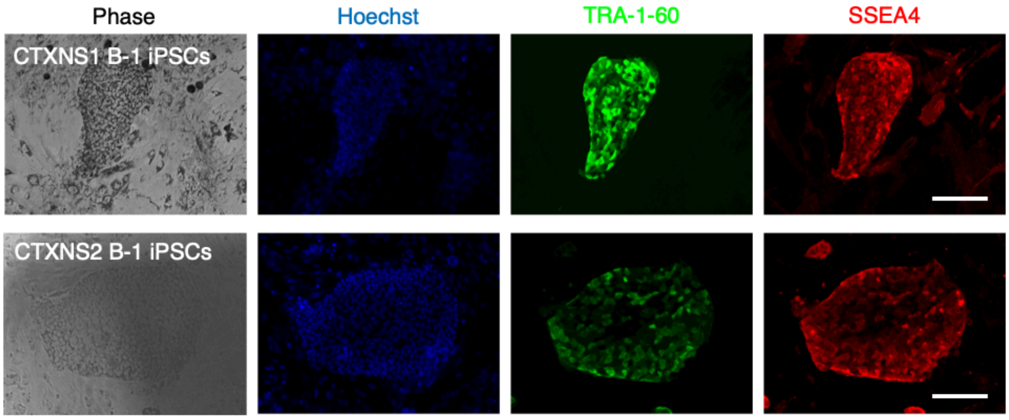

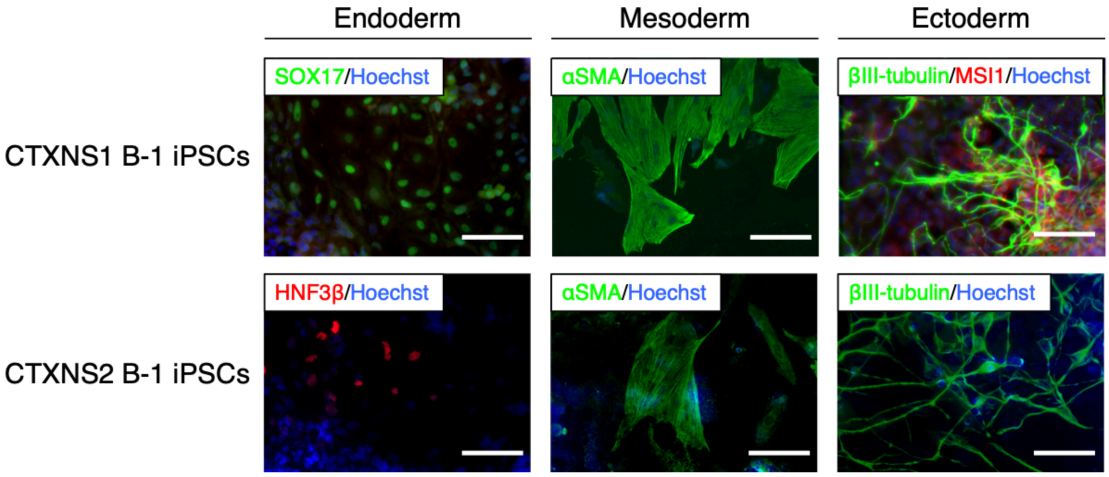

Also, by genomic PCR analysis, we found the removal of episomal vectors from the iPSCs at passage 5 as shown on the right, and female origin of both the iPSC lines were confirmed (Figure S4C).

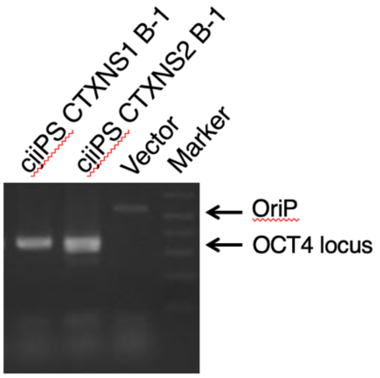

Reprogramming of NSCs toward a pluripotent state has been previously reported using mouse (Di Stefano et al., 2009; Kim et al., 2008) and human cells (Kim et al., 2009a), but among these reports, there was a variation in iPSC derivation efficiency compared to the reprogramming of fibroblasts (Di Stefano et al., 2009; Kim et al., 2009a; Kim et al., 2008). In contrast, we clearly demonstrated that marmoset NSCs can be reprogrammed to iPSCs more efficiently than fibroblasts (Figure 1). Thus, the transcriptional similarity of the iNSLCs to NSCs may provide an explanation for the successful conversion of the iNSLCs into a pluripotent state.

Note S5. Derivation of human iNSLCs.

To validate the applicability of our reprogramming methodology to somatic fibroblasts of other species beside marmosets, we attempted to reprogram human Bj fibroblasts using the same method. Following transfection of the EP-A or EP-B vector set and induction using NSM for three weeks, we observed the emergence of primary colonies, which were positive for MSI1, SOX2 and PAX6 (as shown below, scale bars = 100 μm).

But these cells were negative for representative human PSC markers such as TRA-1-60, SSEA4, SSEA3 and TRA-1-81 (as shown below, scale bars = 100 μm).

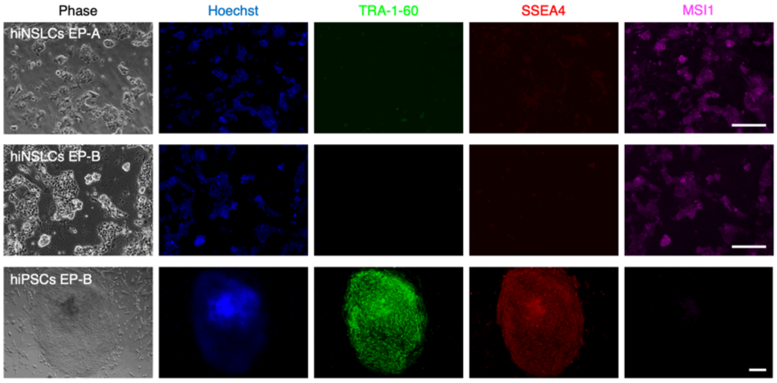

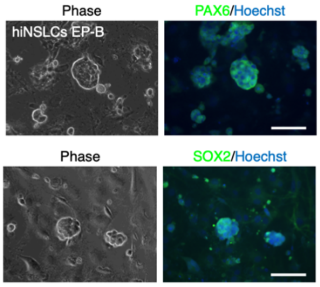

This was quite distinct from hiPSCs, which were derived from Bj fibroblasts using a conventional iPSC medium and EP-B vector set. When using the EP-B vector set, the derivation efficiency of primary iNSLC colonies using NSM was 0.016 ± 0.002% (n = 3, independent experiments), which was significantly higher than that when using a conventional hiPSC medium, 0.003 ± 0.002% (n = 3, independent experiments).

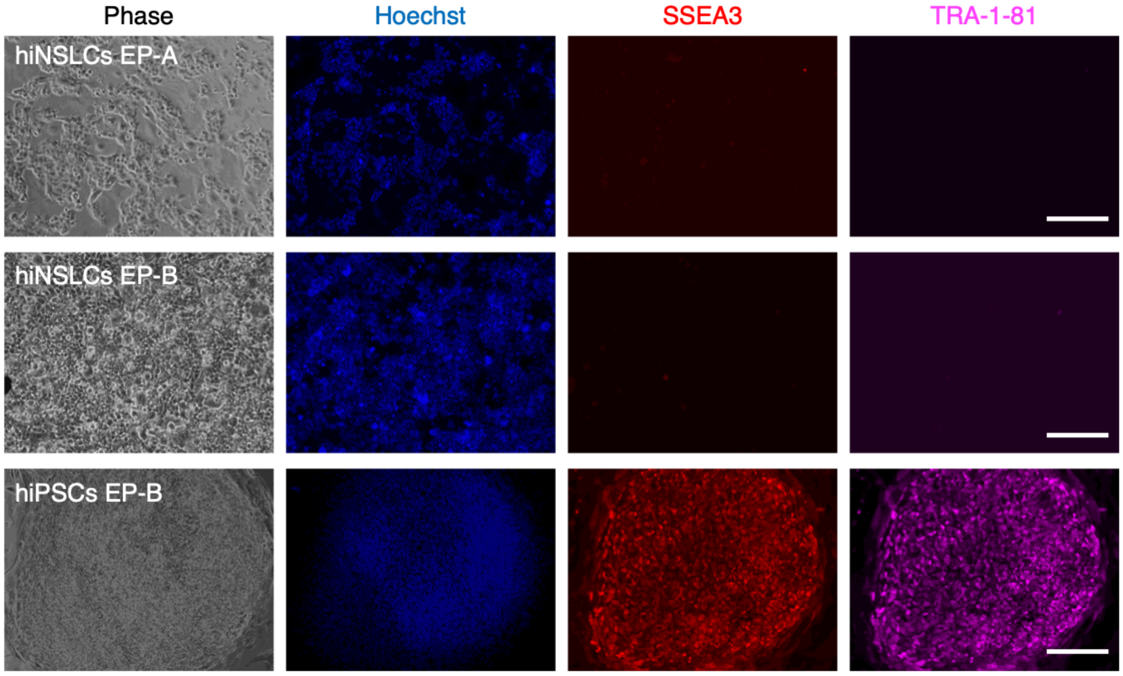

To assess the neural differentiation potential of the human iNSLCs, we performed the direct neurosphere formation assay (Note S2 and Figure S6G) (Yoshimatsu et al., 2019a). Although few cells (less than 1%) differentiated into βIII-tubulin-positive neurons by the original protocol (data not shown), prolonged adherent culture (1.5 month) resulted in robust neural differentiation (~ 80% βIII-tubulin and MAP2-double positive cells; as shown in the right, scale bar = 100 μm), showing their high neural differentiation potential

and comparatively slower differentiation kinetics than that of marmoset iNSLCs.

Moreover, we performed mRNA-seq using RNA from human samples including Bj fibroblast-derived iNSLCs (BjFibro_EPB_NSM_d5 and P1), iPSCs and original fibroblasts with reference to previously deposited data of human naïve-like or primed-state ESCs (Chan et al., 2013). Hierarchical clustering analysis using the expression of major pluripotency/ectoderm-related genes indicated that the iNSLCs harbored a unique gene expression property distinct from that of both naïve-like and primed-state PSCs (as shown below, Log_2FC scaling).

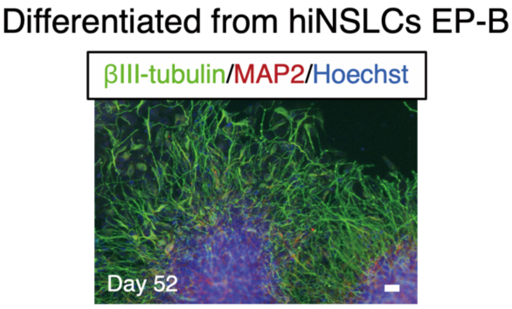

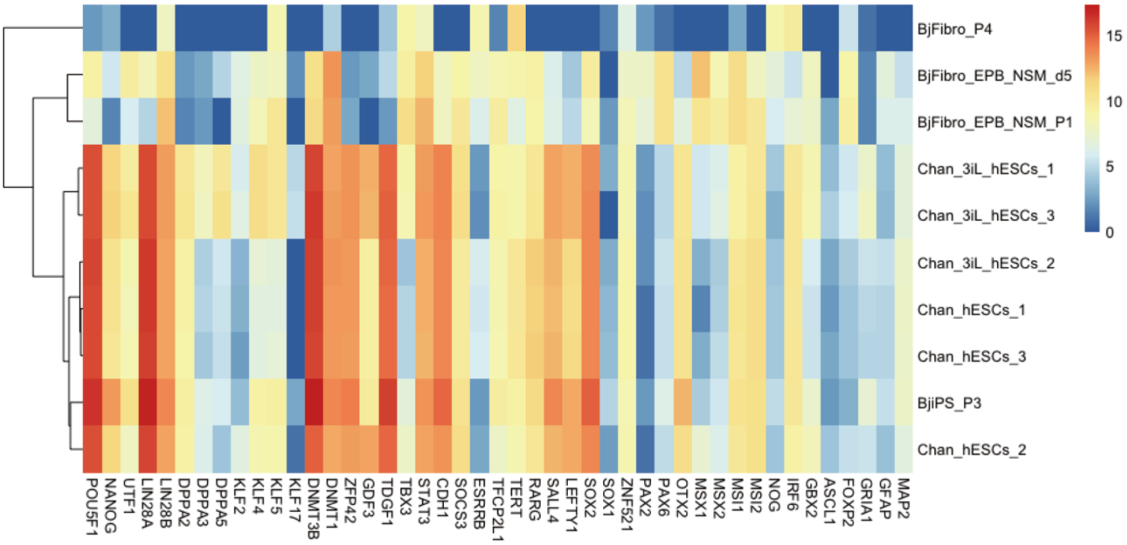

Based on these findings, we speculated that in our method, somatic fibroblasts from various mammalian species are likely to be reprogrammed to pluripotency via a route that passes through an NSC-like state.

Note S6. Transcriptomic analysis of canine fibroblasts, iNSLCs and iPSCs (related to Figure 6).

As shown on the right, by PCA using mRNA-seq data, we found that the primary colony-forming cells such as putative canine iNSLCs showed a clearly distinct profile of gene expression from the K9 iPSC #1−3 and original fibroblasts.

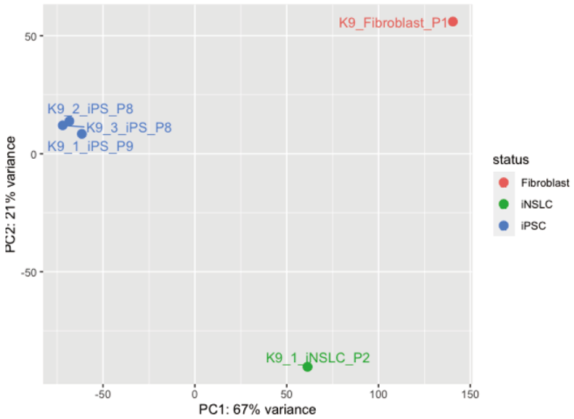

In addition, the iNSLCs showed enriched expression of some NSC/neuroblast markers such as *ASCL1* and *DCX*, while the iPSCs showed strong expression of major PSC markers including *POU5F1* (*OCT4*), *DNMT3A*, *DNMT3B* and *LIN28A* (*LIN28*), as shown below (Log_2FC scaling).

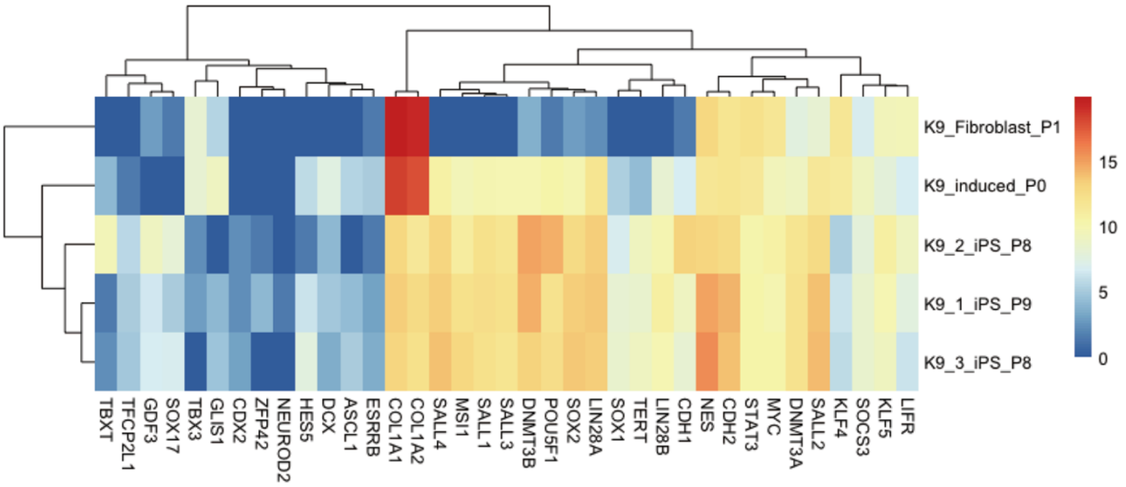

Note S7. Culture conditions for enhancement of PSC marker expression in ciPSCs (related to Figure 6).

In the culture condition using ESM, canine iPSCs stained weakly or negatively for NANOG, SSEA4 and TRA-1-60 (as shown below, scale bars = 100 μm).

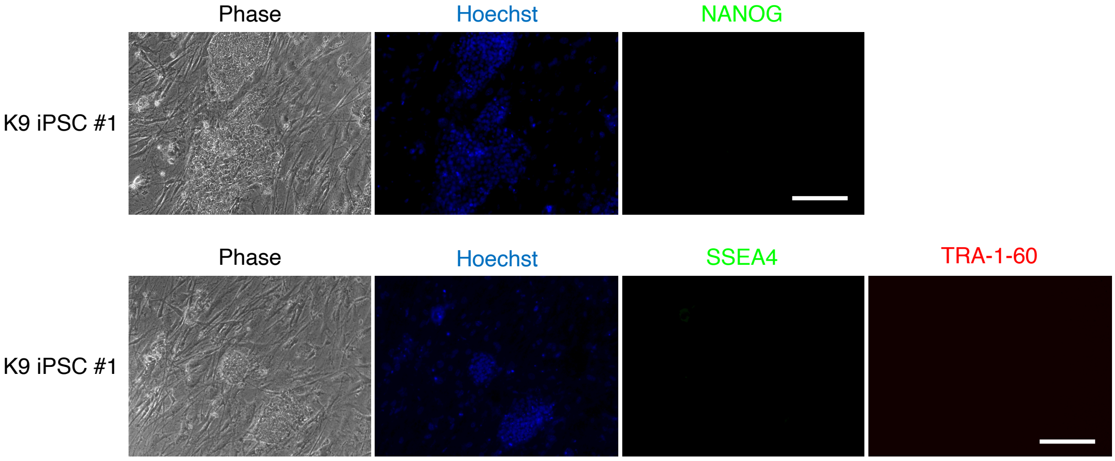

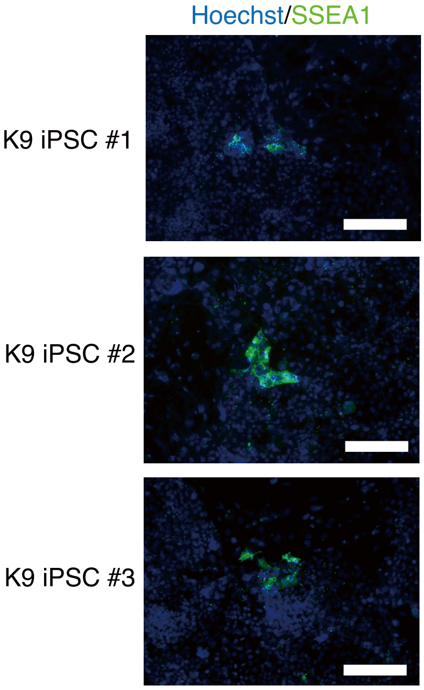

Among the PSC-related surface markers, only SSEA1 was positive for a small portion (~ 1%) of the canine iPSCs (as shown on the right, scale bars = 100 μm), while SSEA3, SSEA4, TRA-1-60 and TRA-1-81 were negative (data not shown).

To explore culture conditions which could enhance PSC marker expression in the ciPSCs, we tested a condition recently reported for the culture of expanded pluripotent stem cells (EPSCs) in human and porcine cells (Gao et al., 2019), since it has been reported that the EPSC culture condition enabled the derivation of transgene-independent porcine iPSCs with robust PSC marker expression for the first time (Du et al., 2015). Although our initial attempts were not successful because of the slow cell growth of ciPSCs in the original EPSC medium (data not shown), we discovered that the supplementation of bFGF (10 ng/ml) and TGF-β1 (10 ng/ml) in the EPSC medium, named EPSbt mem hereon, enabled the expansion of ciPSCs. As shown below (scale bars = 100 μm), by immunocytochemistry, we found that canine iPSCs (K9 iPSC #1) became strongly positive for OCT4, SOX2 and also SSEA1, whose expression was reported in several studies using canine ESC-like cells (Hatoya et

al., 2006; Schneider et al., 2007) and ciPSCs (Tsukamoto et al., 2018). Also, the iPSCs in EPSbt mem were weakly positive for NANOG and TRA-1-60, but still negative for SSEA3 and TRA-1-81.

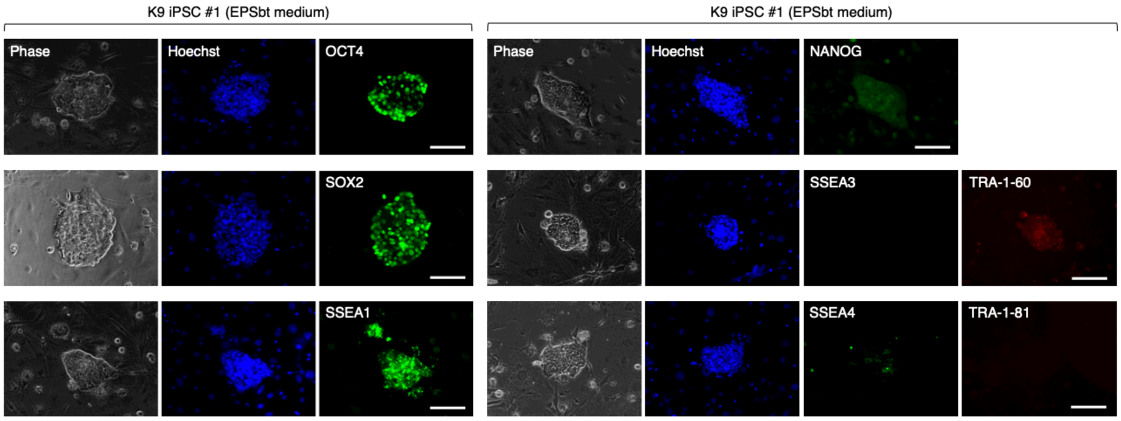

Note S8. Assessment of the *in vivo* three-germ-layer differentiation potential of ciPSCs through teratoma formation.

As shown below right (scale bar = 100 µm), the *in vivo* three-germ-layer differentiation potential of canine iPSCs (K9 iPSC #1) was assessed by teratoma formation (a macroscopic image of the teratoma is shown below left) in NOD/SCID mice. As a result, the ciPSC differentiated into ectoderm (neural tube and sebaceous glands), mesoderm (cartilage and follicular epithelium) and endoderm (glandular epithelium).

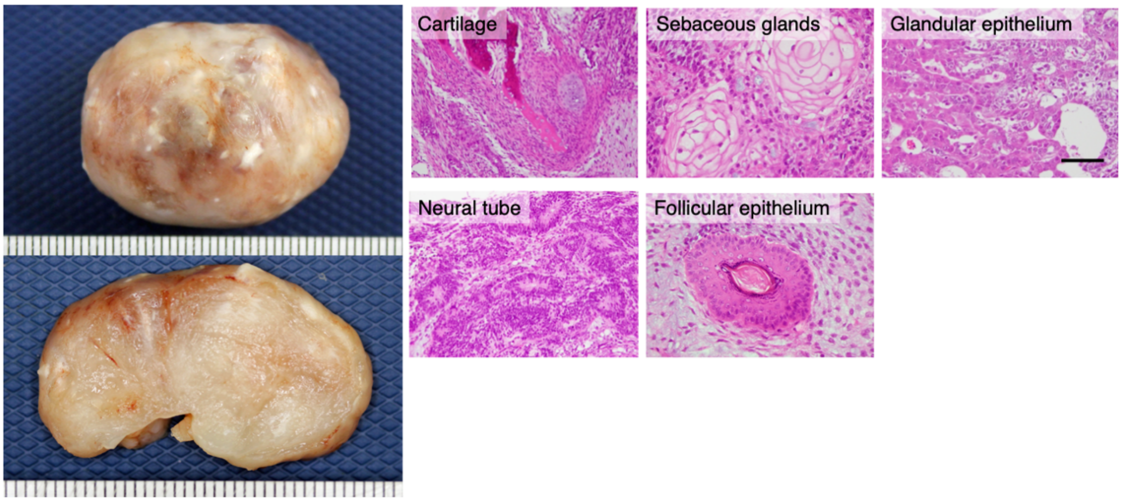

Note S9. Transcriptomic analysis of porcine fibroblasts, iNSLCs and iPSCs (related to Figure 7).

We also performed mRNA-seq analysis for dissecting the gene expression profiles of the porcine cells, using deposited data of piPSCs and early embryos from a previous study (Secher et al., 2017). By hierarchical clustering analysis using the expression of major pluripotency/ectoderm/fibroblast-related genes, N01F iPSC #1−2 (N01F N1−2 iPS) were closely clustered with epiblasts and gastrulation epiblasts, which may reflect their primed-state pluripotency (as shown below, Log_2FC scaling). We note that, distinct from marmoset and canine cells, the difference in gene expression property between cells during induction in NSM (putative iNSLCs; N01F_induced_P1) and piPSCs were comparatively subtle, while the putative iNSLCs showed enhanced expression of multiple ectodermal markers, including *SOX1*, *ASCL1*, *NEUROD1*, *DCX* and *PAX6*.

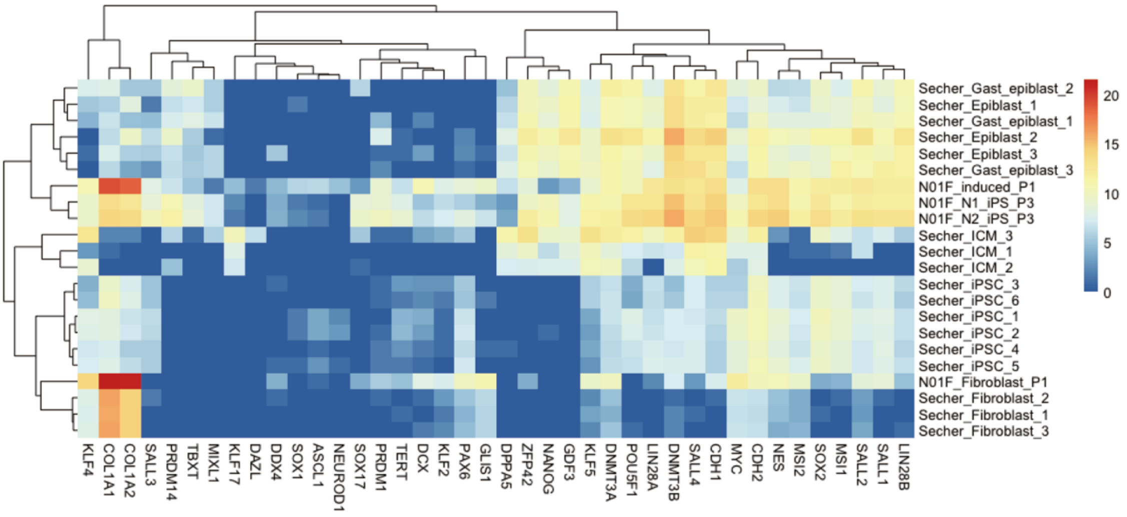

Note S10. Culture conditions for enhancement of PSC marker expression in piPSCs (related to Figure 7).

AP staining and immunocytochemical analyses showed that the piPSCs (N01F iPSC #1) strongly expressed AP, OCT4 and SOX2 (Figure 7C−E), while these cells were negative for NANOG, SSEA4 and TRA-1-60, as shown below (scale bars = 100 µm).

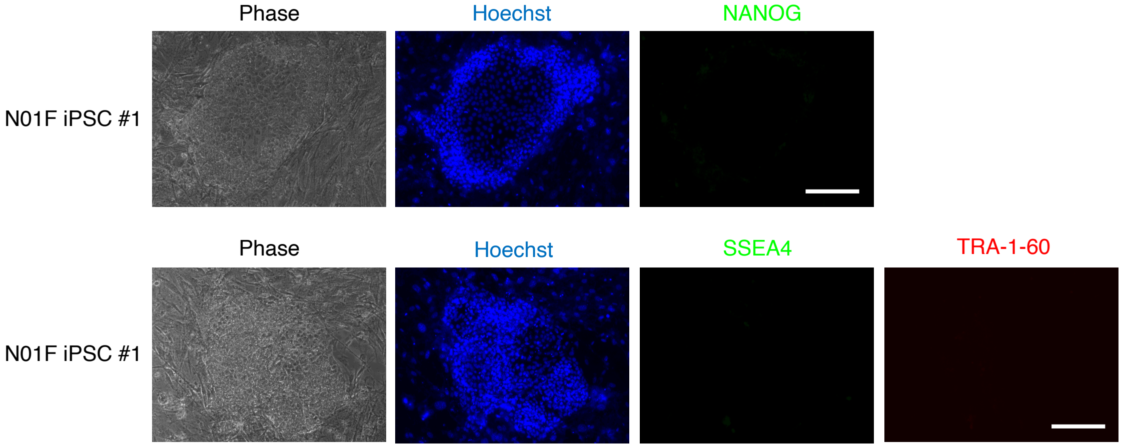

To enhance PSC marker expression of the piPSCs, we also tested the EPSbt mem for culturing the piPSCs. As shown below (scale bars = 100 µm), immunocytochemical analysis revealed that porcine iPSCs (N01F iPSC #1) became strongly positive for OCT4, SOX2, SSEA1, NANOG, SSEA3 and SSEA4, while still negative for TRA-1-60 and TRA-1-81.

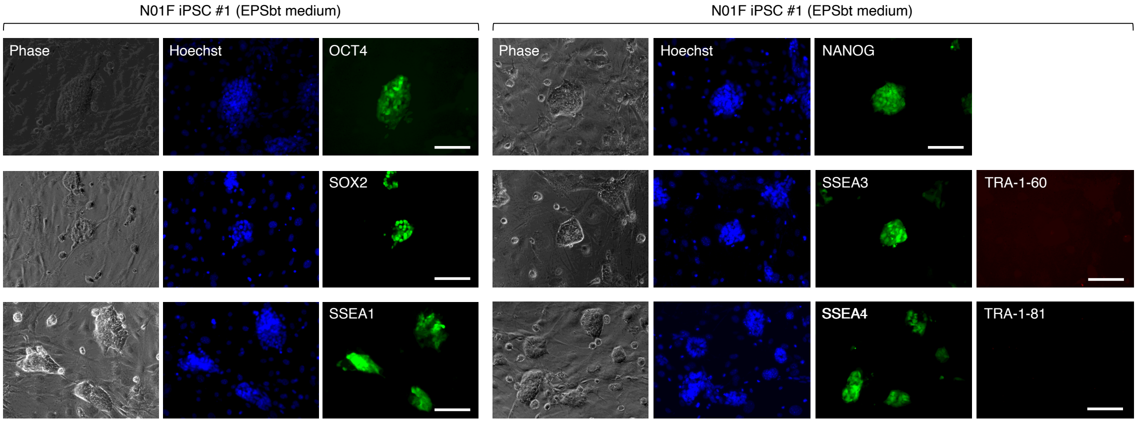

In addition, these cells retained the three-germ layer differentiation capacity by EB formation as shown below (scale bars = 100 µm).

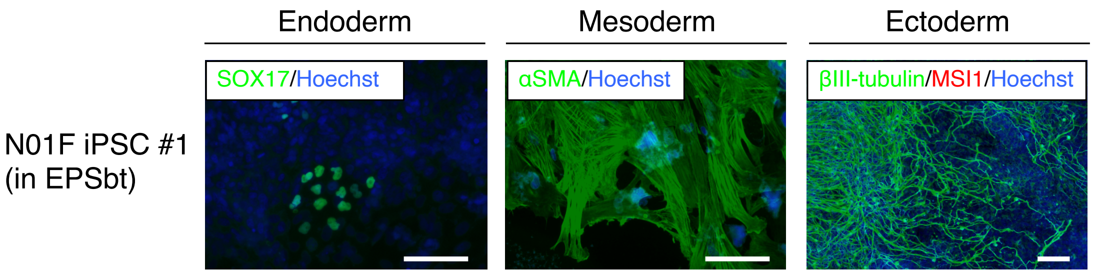

Note S11. Two possible mechanisms of transgene-free iPSC derivation from fibroblasts through an NSC-like state (related to the Discussion section in the main manuscript).

First, when using chemical inhibitors, it has been suggested that the derivation of NSC-like cells from fibroblasts is more efficient than the direct derivation of iPSCs, which favor transgene expression over chemical induction for their generation. For instance, Lu *et al*. reported the derivation of neural progenitors from fibroblasts of humans and macaque monkeys using a medium containing N2/B27 supplements and LIF/CHIR/SB431542 (a TGF-β inhibitor), which are similar to the components of NSM, as well as Sendai virus vectors harboring *OCT4*, *SOX2*, *KLF4* and *C-MYC* (Lu et al., 2013). The existence of this phenomenon was also supported by similar studies using somatic fibroblasts of mice (Kim et al., 2011) and humans (Wang et al., 2013), which emphasizes a sharp difference from the conventional reprogramming route of hiPSCs through an incipient mesendodermal-like state (Takahashi et al., 2014). Although Nakajima-Koyama *et al*. reported the reprogramming of murine adult astrocytes toward a pluripotent state through an NSC-like state (Nakajima-Koyama et al., 2015), and the several studies implied the existence of an NSC-mediated reprogramming route (Kim et al., 2011; Lu et al., 2013; Wang et al., 2013), substantial demonstration of the somatic cell-to-NSC-to-PSC reprogramming phenomenon has not been performed for any non-ectodermal cells including somatic fibroblasts in any mammalian species.

Second, NSCs have a higher potential to be reprogrammed into iPSCs than fibroblasts. Although there is a variation of results among reports, Di Stefano *et al.* showed that NSCs were reprogrammed into iPSCs more efficiently than embryonic fibroblasts in mice (Di Stefano et al., 2009). Additionally, for reprogramming NSCs toward pluripotency, only *OCT4* overexpression was required in both mouse (Kim et al., 2009b) and human NSCs (Kim et al., 2009a). Taken together, it is plausible that the

iNSLCs in our study, which continued to express exogenous transgenes and were transcriptionally similar to NSCs, possess a high potential to be converted into iPSCs.

Note S12. Significance of the derivation of transgene-free ciPSCs and piPSCs in this study (related to the Discussion section in the main manuscript).

Earlier studies on the generation of ciPSCs used transgene-integrating viral vectors, and reported the dependency on transgene expression for the maintenance of the pluripotent state (Lee et al., 2011; Luo et al., 2011; Nishimura et al., 2017; Shimada et al., 2010). More recently, two studies demonstrated the generation of transgene-free ciPSCs using Sendai virus vectors (Chow et al., 2017; Tsukamoto et al., 2018). However, to our knowledge, the present study is the first report to have utilized non-viral methods for deriving transgene-free ciPSCs.

Although significant efforts have been made to derive transgene-free piPSCs (Cong et al., 2019; Ogorevc et al., 2016), multiple studies reported the difficulty in deriving transgene-free iPSCs from porcine fibroblasts, even with intensive drug selection (Du et al., 2015; Wu et al., 2009). Importantly, although the pig is the only non-rodent mammalian species reported to have achieved successful germline-transmitting chimera formation from iPSCs through blastocyst injection (West et al., 2011), residual expression of transgenes affected the developmental potential of the iPSCs, which resulted in the stillborn or premature death of the offspring (West et al., 2011). More recently, Gao *et al.* successfully derived piPSCs using a PiggyBac-transposition-based Dox-inducible vector system and the EPSC medium. Unlike some earlier studies (Du et al., 2015; Esteban et al., 2009; Ezashi et al., 2009; Wu et al., 2009), piPSCs derived by Gao *et al.* retained pluripotency without Dox supplementation i.e. transgene expression (Gao et al., 2019). However, for the first time, this present study succeeded in the complete removal of the transgene sequences from the piPSCs.

Supplemental Figure Legends

Figure S1 (related to Figure 1). Derivation of primary colonies from marmoset fibroblasts using episomal vectors.

(A) Representative bright-field (BF) and green-fluorescence (EGFP) images of E01F fibroblasts 4 days after transfection of the EP-A vector set. Scale bars = 100 μm. (B) Representative images of E01F and E02M fibroblasts 30 days after transfection of the

EP-A vector set, which were cultured in ESM. No colony was derived. Scale bars = 200 μm.

(C) Immunocytochemical staining of primary colonies derived from E01F fibroblasts in NSM using the SOX2 antibody. Hoechst was used for blue nuclear staining. Scale bars = 100 μm.

(D) AP staining of primary colonies derived from embryonic E01F fibroblasts (EP-A and EP-B transfected) in NSM. Cells stained dark purple were AP-positive. Scale bars = 200 μm.

(E) Immunocytochemical staining of primary colonies derived from E01F fibroblasts (EP-A and EP-B transfected) in NSM using primary antibodies of PSC markers such as TRA-1-60 and SSEA4. Scale bars = 100 μm.

(F) Immunocytochemical staining of primary colonies derived from E01F fibroblasts (EP-B transfected) in NSM using MSI1 and TRA-1-60 antibodies. Scale bars = 100 μm.

Figure S2 (related to Figures 2−3). Derivation and characterization of marmoset transgene-free iPSCs.

(A) AP staining of twelve iPSC lines derived from adult marmosets (I5061F and CM421F). Scale bars = 500 μm.

(B) Immunocytochemical staining of eight iPSC lines derived from adult marmosets using TRA-1-60 and SSEA4 antibodies. Scale bars = 100 μm.

(C−H) qPCR analysis of iPSCs using specific primers for endogenous (endo) PSC marker genes (*OCT4*, *NANOG*, *SOX2*, *KLF4*, *ZFP42*, *LIN28*, *DPPA5* and *TERT*) (n = 3, independent experiments).

Figure S3 (related to Figures 2−3). DNA analysis.

(A−D) Genomic PCR analysis of iPSCs derived from embryonic (A) and adult marmosets (B) for the detection of residual episomal vectors using specific primers for the OriP sequence (Yu et al., 2009). Primers for the endogenous marmoset *OCT4* locus were used as an internal control. PCR analyses shown in (C) and (D) were performed to independently amplify OriP and *OCT4* sequences, which was simultaneously performed in (A).

(E) Assessment of the OriP detection sensitivity of PCR using pCE-hUL, supplemented 100 pg, 10 pg, 1 pg, or 0.1 pg in each PCR solution. Approximately, 0.1 pg of pCE-hUL

(11235 bp) is 8.13×10^3 copies. For the genomic PCR analysis, we used 100 ng of genomic DNA in each PCR solution, which is approximately equivalent to 1.5×10^4 copies of marmoset genomic DNA (approximately 30 Mbp).

(F) Schematic of episomal vectors used in this study (vector sets were shown in Figure 1A) and the binding sites of the primers (black arrows) for each episomal vector. Each primer set was abbreviated as following in Figure S3D−F: OC, SK, UL, MP, KN and KG.

(G) The primers specific for each vector were validated by PCR using the genomic DNA of E01F fibroblasts (WT; negative control) and EP-B transfected E01F fibroblasts (Day4; positive control)

(H) Genomic PCR analysis for iPSCs and iNSLCs derived from embryonic marmosets using specific primers for each episomal vector. Genomic DNA extracted from two embryo-derived OriP(−) iPSCs (E01F A-2-2 and E02M B-0-7), two embryo-derived OriP(+) iPSCs (E01F A-2-4 and E02M B-0-11), two embryo-derived OriP(+) iNSLCs (E01F A-2 and E02M B-4), and two adult-derived OriP(−) iPSCs (I5061F B-3-3 and CM421F B-0-12) were used.

(I) Genomic PCR analysis for iNSLCs that were competent for primed conversion (E01F A-2 iNSLC P1 and I5061F B-3 iNSLC P1).

Figure S4 (related to Figure 2 and 3). Karyotyping analysis of marmoset iPSCs.

(A) Chromosome counting of six marmoset iPSC lines (E01F A-2-2, E01F A-2-6, E01F A-2-7, E02M B-0-7, I5061F B-3-3 and CM421F B-0-4) by G-banding. Fifty cells were used in each cell line (except I5061F B-3-3, which we used forty-three cells). Gray bars show euploid (2n = 46) cells. Numerics on bars show the number of counted cells (in fifty cells) harboring each number of chromosomes.

(B) Q-banding-based karyotyping of euploid cells from six marmoset iPSC lines (E01F A-2-2, E01F A-2-6, E01F A-2-7, E02M B-0-7, I5061F B-3-3 and CM421F B-0-4).

(C) Sex of the marmoset ESCs and iPSCs used in this study was validated by PCR using primers specific for the marmoset *GAPDH* (control) and *SRY* loci. Only DSY127 and E02M B-0-7 showed SRY-positive PCR bands. High resolution images of karyotyping are shown in Supplementary Data 3.

Figure S5. PGCLC induction from marmoset PSCs.

(A) Graphical schematic of reporter constructs (*BLIMP1-Venus* and *VASA-tdTomato*) for

marmoset iPSCs. Using *Cas9/gRNA* and *BLIMP1-Venus* targeting vector as described previously (Yoshimatsu et al., 2020; Yoshimatsu et al., 2019c), the endogenous *BLIMP1* locus was targeted (top). Following hygromycin selection, and subsequent G418 and ganciclovir selection, the resultant iPSCs harbored the *BLIMP1-Venus* knock-in alleles and VASA-tdTomato transgene (bottom). Black and gray boxes indicate endogenous exons (coding sequence and UTR) of *BLIMP1*. ITR, *Piggybac* inverted terminal repeats; PGK, Mouse *phosphoglycerate kinase 1* promoter; Hyg ΔTK (HygTK), *hygromycin resistance gene* fused to the N-term-truncated *thymidine kinase*; pA, polyadenylation signal sequence; Neo; *neomycin resistance gene*. Instead of neomycin, G418 (an analogue of neomycin) was used for the selection experiment.

(B) Genotyping PCR of *BLIMP1* targeted iPSCs. Eight iPSC clones after hygromycin selection were genotyped for the detection of *BLIMP1-Venus-HygTK* knock-in (top). The homozygous knock-in clone #8 (named BV8) was used for transfection of a *HyPBase* expression vector and *VASA-tdTomato* reporter. Following G418 and ganciclovir selection, the excision of the PGK-HygTK cassette was confirmed by PCR (bottom). The clone #1 (named BV8VT1) was used for further induction experiments.

(C) Timetable of PGCLC induction (from Day 0 ~ 10). See Supplementary Methods for the detail.

(D) Transgene vectors of the dexamethasone/doxycycline-inducible *SOX17/BLIMP1* overexpression system. CAG, CAG promoter; rtTAM2, *reverse tetracycline-controlled transactivator M2*; IRES, internal ribosome entry site from *encephalomyocarditis virus*; TRE, tetracycline-responsive promoter element; Puro ΔTK, *puromycin resistance gene* fused to the N-term-truncated *thymidine kinase*; HS4, chicken *β-globin* insulator.

(E) Representative phase-contrast/fluorescence images of Day9 aggregates from marmoset ESCs (No.40 BVSCVT2 (Yoshimatsu et al., 2019c) and DSY127 BV8VT1 (Yoshimatsu et al., 2020)) and E01F A-2-2 BV8VT1 iPSCs. Scale bars, 100 μm.

(F) FACS analysis of BLIMP1-Venus fluorescence in single-cell-dissociated Day10 aggregates from marmoset ESCs and iPSCs. Venus fluorescence was detected by the FITC filter. 7-AAD (APC filter) was used for the removal of dead cells.

(G) PCA of marmoset ESCs and iPSCs (PSCs; green dots) and PGCLCs (Day10 BLIMP1-Venus-positive cells; red dots) and a whole testis from a 3-year-old marmoset (Adult_Testis; a blue dot).

(H) Volcano plot of DEG analysis (PGCLC vs PSC samples).

(I) Heatmapping of pluripotency and germ cell linage-related marker genes.

Figure S6 (related to Figure 4). Characterization of marmoset iNSLCs.

(A) qPCR analysis of early ectodermal markers (*SOX1* and *ZFP521*) in iNSLCs.

(B) qPCR analysis of early-mesodermal (*T*) and endodermal (*SOX17*) markers in iNSLCs.

(C−D) qPCR analysis of PSC markers (*OCT4*, *NANOG*, *SOX2*, *KLF4*) and NSC markers (*SOX1* and *PAX6*) in the iNSLCs at early passages (P1−2). For *OCT4* and *NANOG*, primers for both endogenous (endo) and exogenous (exo) sequences were used.

(E−F) Immunocytochemical staining of iNSLCs using primary antibodies of NSC (MSI1, PAX6 and SOX2) and neuroblast (DCX) markers. Scale bars = 100 μm.

(G) Timetable for the direct neurosphere formation assay.

(H) Differentiation efficiency of βIII-tubulin-positive neurons (βIII-tubulin(+)) from ESCs, iPSCs and iNSLCs.

(I) Representative images of βIII-tubulin-positive neurons derived from the iNSLCs by the method shown in (A). Scale bars = 100 μm.

(J−L) Immunocytochemical analysis of differentiated cells from the I5061F B-0 iNSLCs, using primary antibodies of βIII-tubulin (neurons), GFAP (glial cells), Islet1 and ChAT (motor neurons), GalC (oligodendrocytes). Scale bars = 50 μm.

Figure S7. Assessment of the gene expression difference between iPSCs and iNSLCs (related to Figure 5).

(A) Volcano plot of DEG analysis (iPSC vs iNSLC samples) using data from bulk mRNA-seq analysis. Significance was defined as fold change (FC) > 5 and $p < 0.01$. Genes that were significantly expressed in iPSCs are shown in warm colors. Genes that were significantly expressed in iNSLCs are shown in cold colors.

(B) Single-cell PCA of No.40 ESCs (n = 4; red dots), an E01F A-2-2 iPSC (n = 1; an orange dot), E01F A-2 iNSLCs at an early passage (P1; n = 65; black dots) and an E01F fibroblast (n = 1; a green dot) using RamDA-seq data.

(C) Box plots of *ASCL1*, *DCX*, *PAX6* and *NEUROG2* (genes significantly expressed in iNSLCs). Triangles show the expression levels in respective (single) cells.

(D) Box plots of *TDGF1*, *UTF1*, *EPCAM*, *ZFP42* and *DPPA2* (genes significantly expressed in iPSCs). Triangles show the expression levels in respective (single) cells.

Figure S1

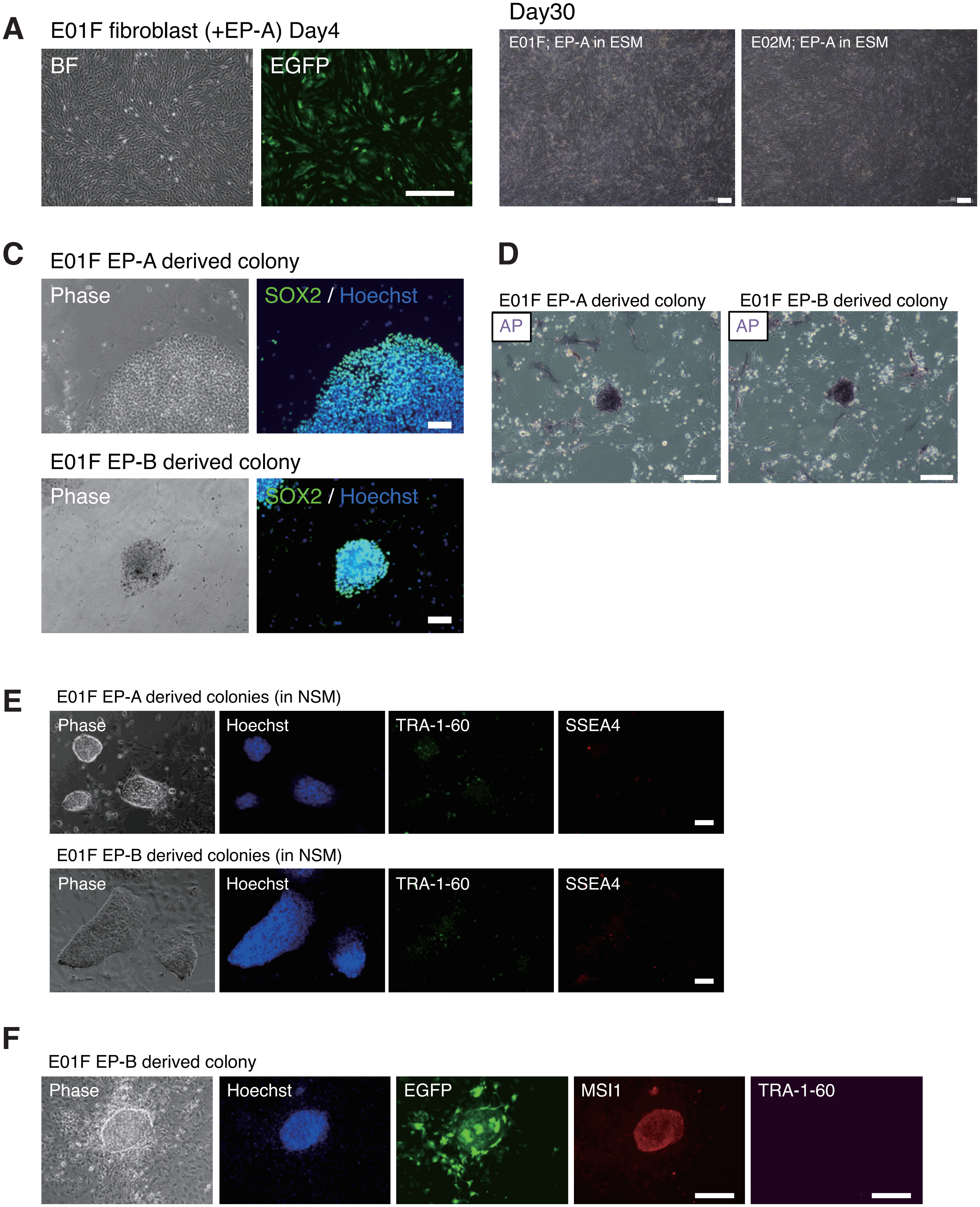
A
E01F fibroblast (+EP-A) Day4
BF
EGFP
B
Day30
E01F; EP-A in ESM
E02M; EP-A in ESM
C
E01F EP-A derived colony
Phase
SOX2 / Hoechst
E01F EP-B derived colony
Phase
SOX2 / Hoechst
D
E01F EP-A derived colony
E01F EP-B derived colony
AP
AP
E
E01F EP-A derived colonies (in NSM)
Phase
Hoechst
TRA-1-60
SSEA4
E01F EP-B derived colonies (in NSM)
Phase
Hoechst
TRA-1-60
SSEA4
F
E01F EP-B derived colony
Phase
Hoechst
EGFP
MSI1
TRA-1-60

Figure S2

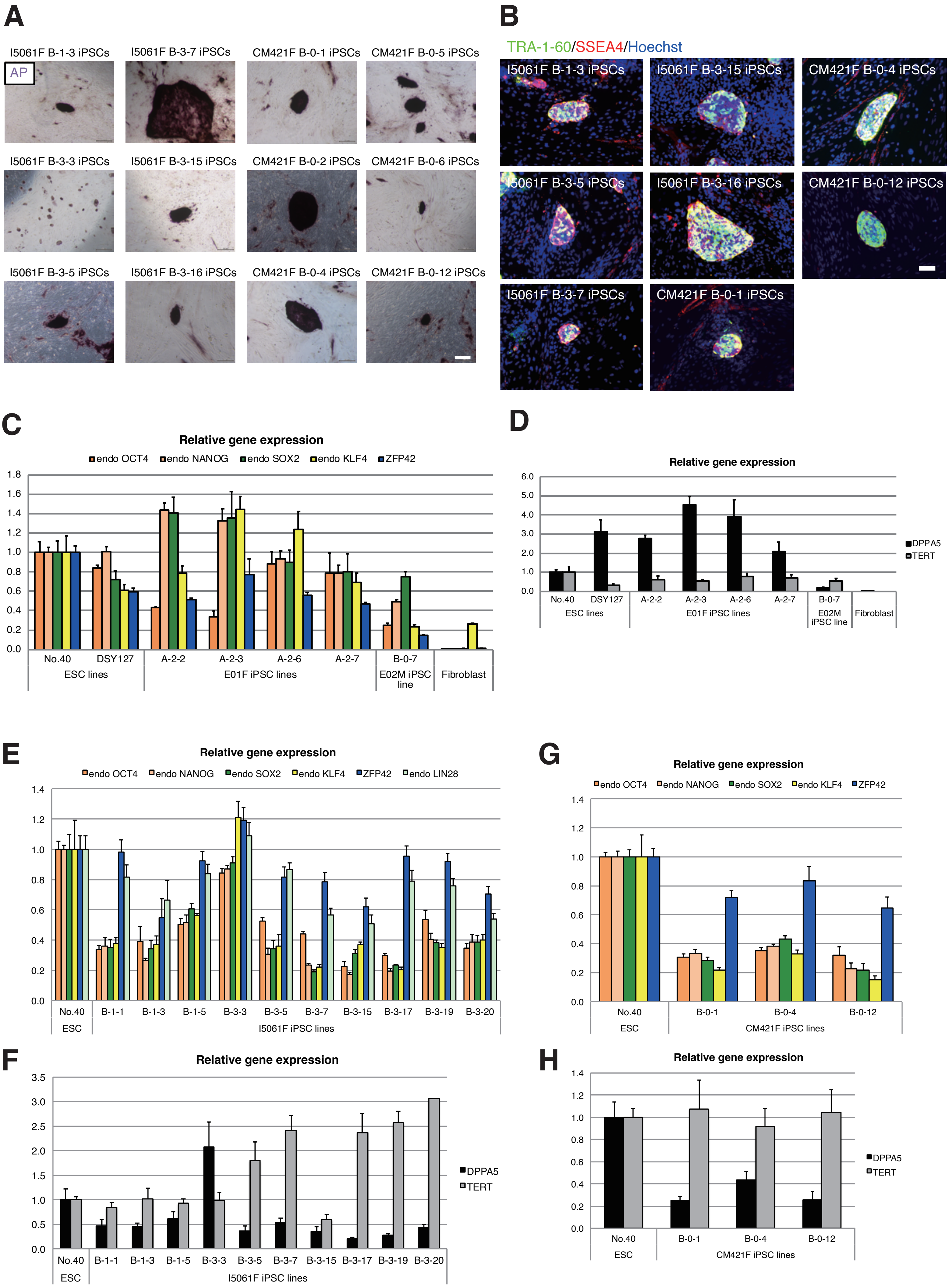
A
I5061F B-1-3 iPSCs
AP
I5061F B-3-7 iPSCs
CM421F B-0-1 iPSCs
CM421F B-0-5 iPSCs
I5061F B-3-3 iPSCs
I5061F B-3-15 iPSCs
CM421F B-0-2 iPSCs
CM421F B-0-6 iPSCs
I5061F B-3-5 iPSCs
I5061F B-3-16 iPSCs
CM421F B-0-4 iPSCs
CM421F B-0-12 iPSCs
B
TRA-1-60/SSEA4/Hoechst
I5061F B-1-3 iPSCs
I5061F B-3-15 iPSCs
CM421F B-0-4 iPSCs
I5061F B-3-5 iPSCs
I5061F B-3-16 iPSCs
CM421F B-0-12 iPSCs
I5061F B-3-7 iPSCs
CM421F B-0-1 iPSCs
C
Relative gene expression
endo OCT4
endo NANOG
endo SOX2
endo KLF4
ZFP42
No.40
DSY127
ESC lines
A-2-2
A-2-3
A-2-6
A-2-7
E01F iPSC lines
B-0-7
E02M iPSC line
Fibroblast
D
Relative gene expression
DPPA5
TERT
No.40
DSY127
ESC lines
A-2-2
A-2-3
A-2-6
A-2-7
E01F iPSC lines
B-0-7
E02M iPSC line
Fibroblast
E
Relative gene expression
endo OCT4
endo NANOG
endo SOX2
endo KLF4
ZFP42
endo LIN28
No.40
ESC
B-1-1
B-1-3
B-1-5
B-3-3
B-3-5
B-3-7
B-3-15
B-3-17
B-3-19
B-3-20
I5061F iPSC lines
F
Relative gene expression
DPPA5
TERT
No.40
ESC
B-1-1
B-1-3
B-1-5
B-3-3
B-3-5
B-3-7
B-3-15
B-3-17
B-3-19
B-3-20
I5061F iPSC lines
G
Relative gene expression
endo OCT4
endo NANOG
endo SOX2
endo KLF4
ZFP42
No.40
ESC
B-0-1
B-0-4
B-0-12
CM421F iPSC lines
H
Relative gene expression
DPPA5
TERT
No.40
ESC
B-0-1
B-0-4
B-0-12
CM421F iPSC lines

Figure S3

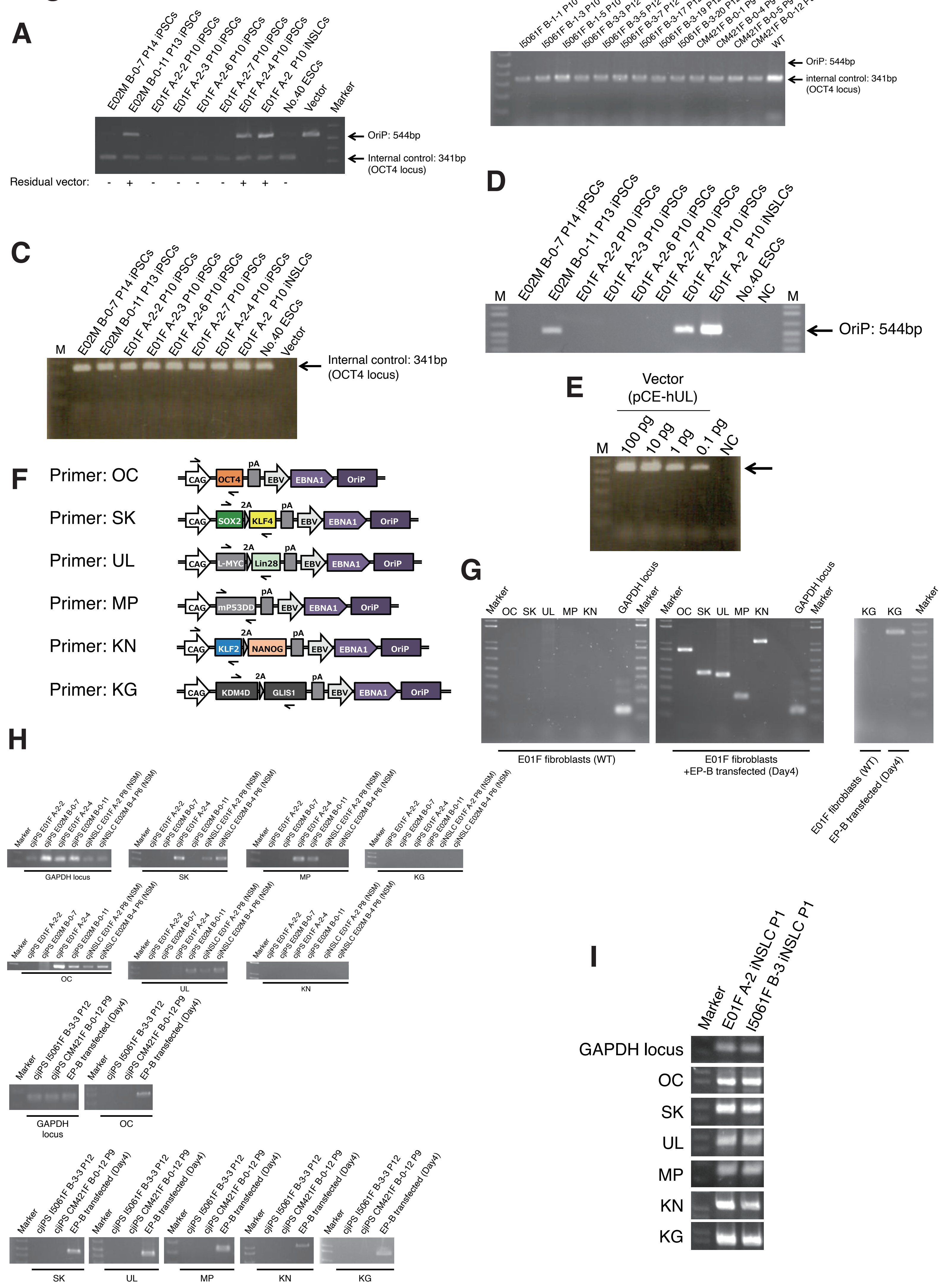
A
E02M B-0-7 P14 iPSCs
E02M B-0-11 P13 iPSCs
E01F A-2-2 P10 iPSCs
E01F A-2-3 P10 iPSCs
E01F A-2-6 P10 iPSCs
E01F A-2-7 P10 iPSCs
E01F A-2-4 P10 iPSCs
E01F A-2 P10 iNSLCs
No.40 ESCs
Vector
Marker
OriP: 544bp
Internal control: 341bp (OCT4 locus)
Residual vector: - + - - - - + + -
B
iPSC clones derived from adult marmosets
I5061F B-1-1 P10
I5061F B-1-3 P10
I5061F B-1-5 P10
I5061F B-3-3 P12
I5061F B-3-5 P12
I5061F B-3-7 P12
I5061F B-3-17 P12
I5061F B-3-19 P12
I5061F B-3-20 P12
CM421F B-0-1 P9
CM421F B-0-4 P9
CM421F B-0-5 P9
CM421F B-0-12 P9
WT
OriP: 544bp
internal control: 341bp (OCT4 locus)
C
M
Internal control: 341bp (OCT4 locus)
D
M
NC
OriP: 544bp
E
Vector (pCE-hUL)
100 pg
10 pg
1 pg
0.1 pg
F
Primer: OC
Primer: SK
Primer: UL
Primer: MP
Primer: KN
Primer: KG
CAG
OCT4
SOX2
KLF4
L-MYC
Lin28
mP53DD
KLF2
NANOG
KDM4D
GLIS1
2A
pA
EBV
EBNA1
OriP
G
Marker
OC SK UL MP KN
GAPDH locus
KG
E01F fibroblasts (WT)
E01F fibroblasts +EP-B transfected (Day4)
E01F fibroblasts (WT)
EP-B transfected (Day4)
H
cjiPS E01F A-2-2
cjiPS E02M B-0-7
cjiPS E01F A-2-4
cjiPS E02M B-0-11
cjiNSLC E01F A-2 P8 (NSM)
cjiNSLC E02M B-4 P6 (NSM)
GAPDH locus
SK
MP
KG
OC
UL
KN
cjiPS I5061F B-3-3 P12
cjiPS CM421F B-0-12 P9
EP-B transfected (Day4)
I
Marker
E01F A-2 iNSLC P1
I5061F B-3 iNSLC P1
GAPDH locus
OC
SK
UL
MP
KN
KG

Figure S4

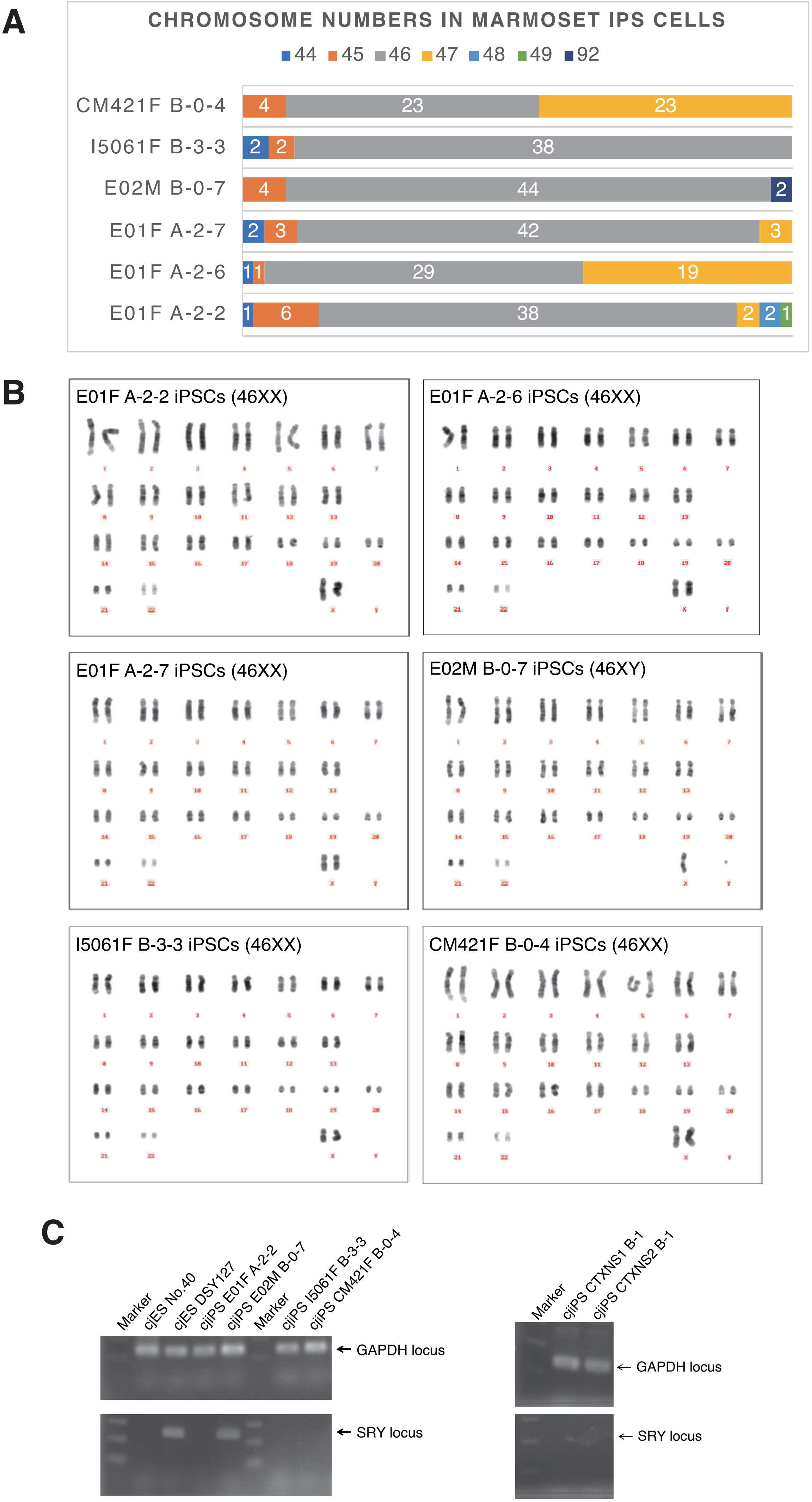
A
CHROMOSOME NUMBERS IN MARMOSET IPS CELLS
44 45 46 47 48 49 92
CM421F B-0-4
I5061F B-3-3
E02M B-0-7
E01F A-2-7
E01F A-2-6
E01F A-2-2
4 23 23
2 2 38
4 44 2
2 3 42 3
1 1 29 19
1 6 38 2 2 1
B
E01F A-2-2 iPSCs (46XX)
E01F A-2-6 iPSCs (46XX)
E01F A-2-7 iPSCs (46XX)
E02M B-0-7 iPSCs (46XY)
I5061F B-3-3 iPSCs (46XX)
CM421F B-0-4 iPSCs (46XX)
C
Marker
cjES No.40
cjES DSY127
cjiPS E01F A-2-2
cjiPS E02M B-0-7
Marker
cjiPS I5061F B-3-3
cjiPS CM421F B-0-4
GAPDH locus
SRY locus
Marker
cjiPS CTXNS1 B-1
cjiPS CTXNS2 B-1
GAPDH locus
SRY locus

Figure S5

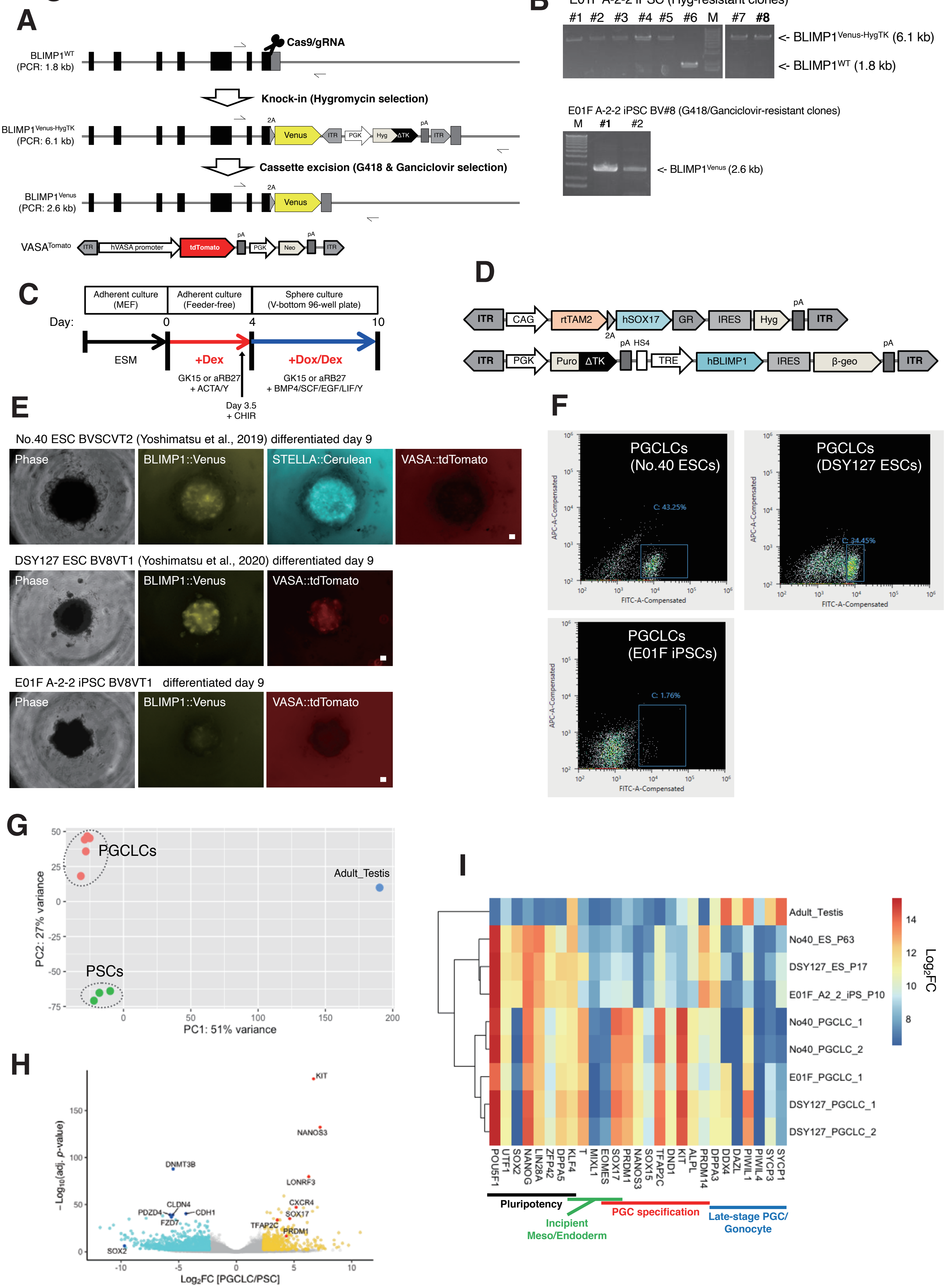

A
BLIMP1WT (PCR: 1.8 kb)
Cas9/gRNA
Knock-in (Hygromycin selection)
BLIMP1Venus-HygTK (PCR: 6.1 kb)
2A
Venus
ITR
PGK
Hyg
ΔTK
pA
Cassette excision (G418 & Ganciclovir selection)
BLIMP1Venus (PCR: 2.6 kb)
VASATomato
hVASA promoter
tdTomato
Neo
B
E01F A-2-2 iPSC (Hyg-resistant clones)
#1 #2 #3 #4 #5 #6 M #7 #8
<- BLIMP1Venus-HygTK (6.1 kb)
<- BLIMP1WT (1.8 kb)
E01F A-2-2 iPSC BV#8 (G418/Ganciclovir-resistant clones)
M #1 #2
<- BLIMP1Venus (2.6 kb)
C
Adherent culture (MEF)
Adherent culture (Feeder-free)
Sphere culture (V-bottom 96-well plate)
Day: 0 4 10
ESM
+Dex
+Dox/Dex
GK15 or aRB27 + ACTA/Y
GK15 or aRB27 + BMP4/SCF/EGF/LIF/Y
Day 3.5 + CHIR
D
ITR CAG rtTAM2 hSOX17 GR IRES Hyg pA ITR
2A
ITR PGK Puro ΔTK pA HS4 TRE hBLIMP1 IRES β-geo pA ITR
E
No.40 ESC BVSCVT2 (Yoshimatsu et al., 2019) differentiated day 9
Phase
BLIMP1::Venus
STELLA::Cerulean
VASA::tdTomato
DSY127 ESC BV8VT1 (Yoshimatsu et al., 2020) differentiated day 9
E01F A-2-2 iPSC BV8VT1 differentiated day 9
F
PGCLCs (No.40 ESCs)
C: 43.25%
PGCLCs (DSY127 ESCs)
C: 34.45%
PGCLCs (E01F iPSCs)
C: 1.76%
APC-A-Compensated
FITC-A-Compensated
G
PGCLCs
Adult_Testis
PSCs
PC2: 27% variance
PC1: 51% variance
H
KIT
NANOS3
DNMT3B
LONRF3
CLDN4
CXCR4
PDZD4
CDH1
SOX17
FZD7
TFAP2C
PRDM1
SOX2
−Log10(adj. p-value)
Log2FC [PGCLC/PSC]
I
Adult_Testis
No40_ES_P63
DSY127_ES_P17
E01F_A2_2_iPS_P10
No40_PGCLC_1
No40_PGCLC_2
E01F_PGCLC_1
DSY127_PGCLC_1
DSY127_PGCLC_2
Log2FC
POU5F1 UTF1 SOX2 NANOG LIN28A ZFP42 DPPA5 KLF4 T MIXL1 EOMES SOX17 PRDM1 NANOS3 SOX15 TFAP2C DND1 KIT ALPL PRDM14 DPPA3 DDX4 DAZL PIWIL1 PIWIL4 SYCP3 SYCP1
Pluripotency
Incipient Meso/Endoderm
PGC specification
Late-stage PGC/ Gonocyte

Figure S6

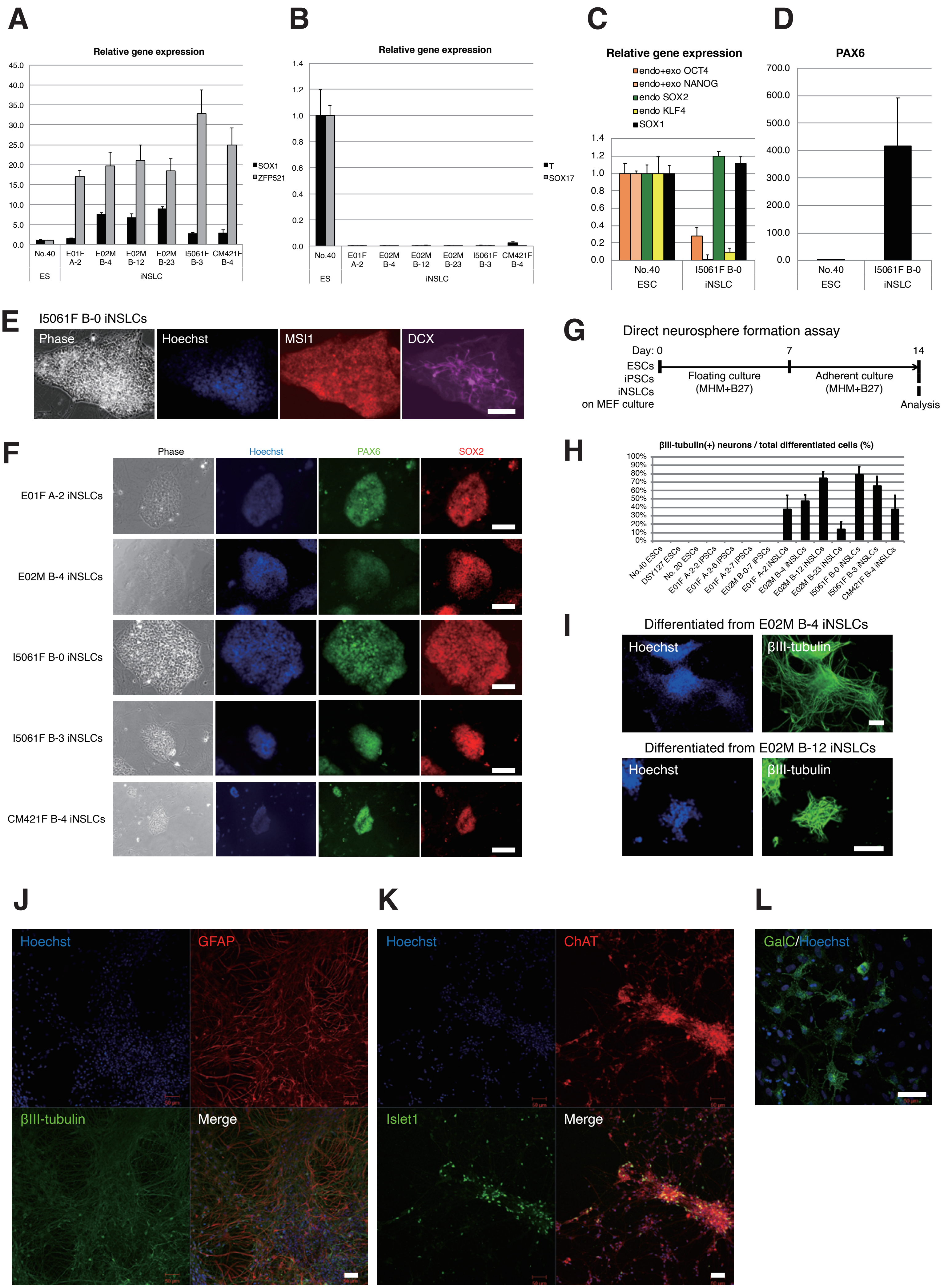
A
Relative gene expression
SOX1
ZFP521
No.40
E01F A-2
E02M B-4
E02M B-12
E02M B-23
I5061F B-3
CM421F B-4
ES
iNSLC
B
Relative gene expression
T
SOX17
C
Relative gene expression
endo+exo OCT4
endo+exo NANOG
endo SOX2
endo KLF4
SOX1
No.40 ESC
I5061F B-0 iNSLC
D
PAX6
E
I5061F B-0 iNSLCs
Phase
Hoechst
MSI1
DCX
F
Phase
Hoechst
PAX6
SOX2
E01F A-2 iNSLCs
E02M B-4 iNSLCs
I5061F B-0 iNSLCs
I5061F B-3 iNSLCs
CM421F B-4 iNSLCs
G
Direct neurosphere formation assay
Day: 0
7
14
ESCs
iPSCs
iNSLCs
on MEF culture
Floating culture (MHM+B27)
Adherent culture (MHM+B27)
Analysis
H
βIII-tubulin(+) neurons / total differentiated cells (%)
No.40 ESCs
DSY127 ESCs
No. 20 ESCs
E01F A-2-2 iPSCs
E01F A-2-6 iPSCs
E01F A-2-7 iPSCs
E02M B-0-7 iPSCs
E01F A-2 iNSLCs
E02M B-4 iNSLCs
E02M B-12 iNSLCs
E02M B-23 iNSLCs
I5061F B-0 iNSLCs
I5061F B-3 iNSLCs
CM421F B-4 iNSLCs
I
Differentiated from E02M B-4 iNSLCs
Hoechst
βIII-tubulin
Differentiated from E02M B-12 iNSLCs
J
Hoechst
GFAP
βIII-tubulin
Merge
K
Hoechst
ChAT
Islet1
Merge
L
GalC/Hoechst

Figure S7

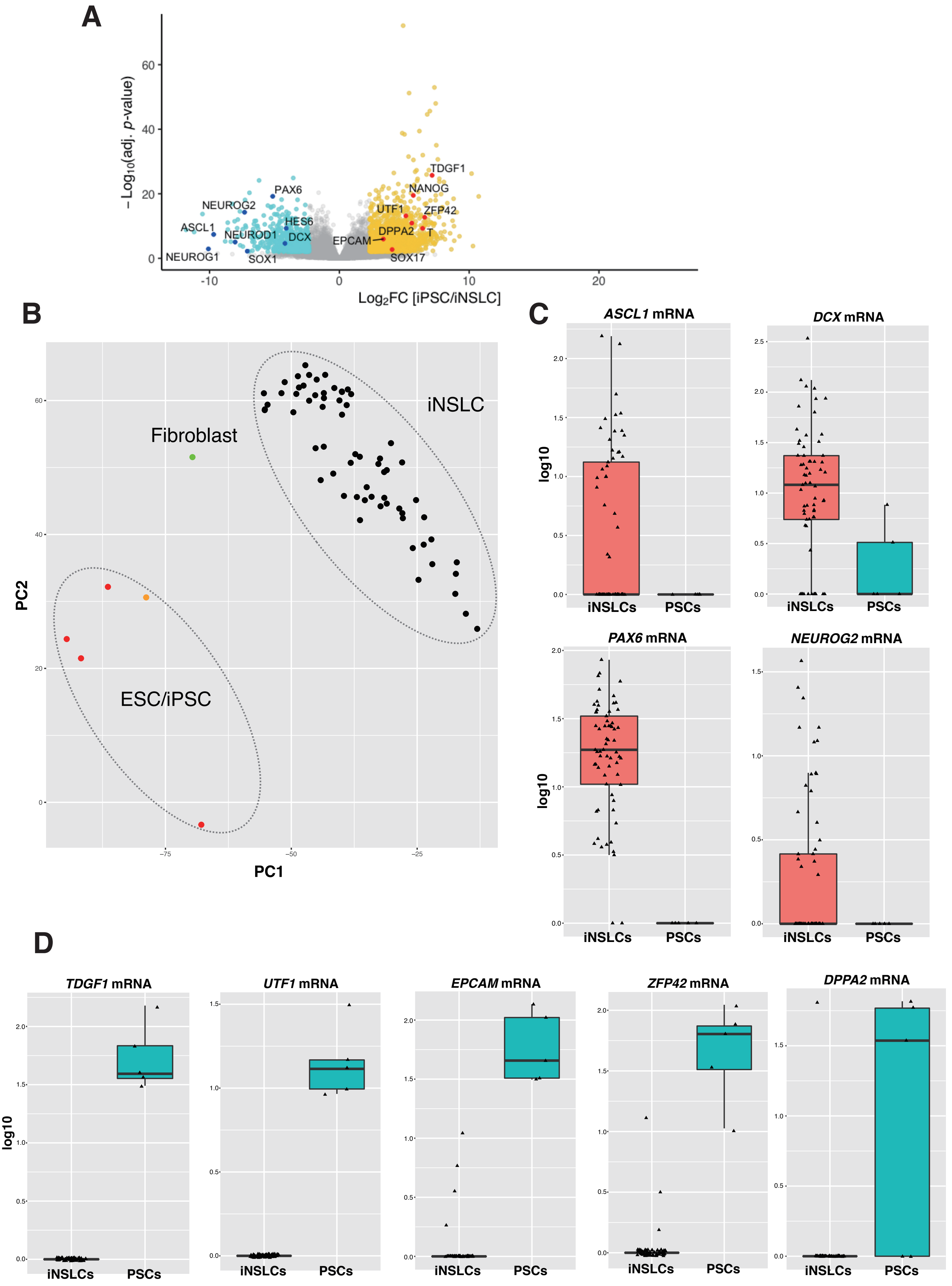

A
−Log10(adj. p-value)
Log2FC [iPSC/iNSLC]
NEUROG2
PAX6
ASCL1
NEUROD1
HES6
DCX
NEUROG1
SOX1
EPCAM
UTF1
DPPA2
SOX17
TDGF1
NANOG
ZFP42
T
B
PC1
PC2
Fibroblast
iNSLC
ESC/iPSC
C
ASCL1 mRNA
DCX mRNA
PAX6 mRNA
NEUROG2 mRNA
log10
iNSLCs
PSCs
D
TDGF1 mRNA
UTF1 mRNA
EPCAM mRNA
ZFP42 mRNA
DPPA2 mRNA

Supplemental Experimental Procedures

Experimental animals.

As summarized in below, two adult marmosets (female, ages 4–6 years), four embryonic marmosets (E95–96, one male and three female), an adult beagle dog (female, 9 years old) and a post-neonatal pig (female, 1.5 months old) were used in the present study. Embryonic marmosets were obtained by Caesarian section as previously described (Shimada et al., 2012).

Animal ID (in this study)	Species	Age*1	Sex	Collected cell origin
E01F	*Callithrix jacchus* (marmoset)	E95	Female	Dorsal skin (Fibroblasts)
E02M	*Callithrix jacchus* (marmoset)	E96	Male	Dorsal skin (Fibroblasts)
I5061F	*Callithrix jacchus* (marmoset)	6.0 years old	Female	Ear skin (Fibroblasts)
CM421F	*Callithrix jacchus* (marmoset)	4.9 years old	Female	Ear skin (Fibroblasts)
CTXNS1	*Callithrix jacchus* (marmoset)	E95	Female	Cerebral cortex (Neural stem cells)
CTXNS2	*Callithrix jacchus* (marmoset)	E95	Female	Cerebral cortex (Neural stem cells)
K9	*Canis lupus familiaris* (dog)	9.0 years old	Female	Ear skin (Fibroblasts)
N01F	*Sus scrofa* (pig)	1 month old	Female	Ear skin (Fibroblasts)

*1: Age when the fibroblasts were collected.

Cell culture

Fibroblasts were collected from the biopsies of dorsal skin (embryonic marmoset) or ear

skin (adult marmosets, an adult dog, and a post-neonatal pig). The fibroblasts were expanded on a 0.1% gelatin-coated tissue culture dish or plate in M10 medium consisting of Dulbecco's Modified Eagle Medium supplemented with 10% inactivated fetal bovine serum and 1% Penicillin/Streptomycin solution (P/S) (all purchased from Thermo Fisher). *In vivo*-derived neural stem cells were collected from the biopsy of cerebral cortexes of embryonic marmosets and were cultured in a suspension culture using MHM medium (Shimada et al., 2012) supplemented with 2% B27, 20 ng/ml human recombinant bFGF (Thermo Fisher), 20 ng/ml human recombinant EGF (EGF; Thermo Fisher), 10 ng/ml human recombinant LIF (LIF; Nacalai Tesque) and 1 μg/ml heparin sodium salt (Nacalai Tesque).

We used three marmoset ESC lines, No. 40 and No. 20 ESCs (CMES40 and CMES20) (Sasaki et al., 2005), and DSY127 (kindly provided by Sumitomo Dainippon Pharma Co., Ltd.), which were cultured as previously described (Nii et al., 2014; Yoshimatsu et al., 2019b). In brief, the ESCs were cultured on 30 Gy-irradiated mouse embryonic fibroblasts (MEFs, 2.5×10^6 cells / 6-well plate or 100mm dish) in ES medium (ESM). ESM consisted of 1x Knockout Dulbecco's modified Eagle's medium (Thermo Fisher) supplemented with 20% Knockout Serum Replacement (KSR; Thermo Fisher), 0.1 mM MEM Non-Essential Amino Acids Solution (NEAA; Sigma), 1 mM L-glutamine (L-glu; Thermo Fisher), 0.2 mM β-mercaptoethanol (2ME; Thermo Fisher), 1% P/S and 10 ng/ml bFGF. For passaging, the ESCs were pre-treated with 10 μM Y-27632 (Thermo Fisher) in ESM at 37°C for an hour. Then, the cells were dissociated by 0.25% trypsin-ethylenediaminetetraacetic acid (Trypsin; Nacalai Tesque), mechanically separated from MEFs, and plated onto new MEFs. Passaging was routinely performed at a 1:20 dilution in the current study.

Transfection, induction, and calculation of colony derivation efficiency.

Vector transfection into fibroblasts was performed as described previously (Debowski et al., 2015; Du et al., 2015). In brief, Nucleofector 2b (Lonza) and Basic Fibroblast Nucleofector Kit (Lonza) were used for introducing a total of 3.65 μg DNA vectors into 1×10^6 fibroblasts using V-13 condition for marmoset fibroblasts, U-23 for canine fibroblasts, T-16 for porcine fibroblasts.

For transfection into fibroblasts using the EP-A set, 0.63 μg pCE-hOCT3/4, 0.63 μg pCE-hSK, 0.63 μg pCE-hUL, 0.63 μg pCE-mp53DD, 0.63 μg pCXLE-EGFP and 0.5

μg pCXB-EBNA1 were added to a 100 μl nucleofection solution, which was then used for transfection using the Nucleofector 2b. For transfection into fibroblasts using the EP-B set, 0.47 μg pCE-hOCT3/4, 0.47 μg pCE-hSK, 0.47 μg pCE-hUL, 0.47 μg pCE-mp53DD, 0.47 μg pCXLE-EGFP, 0.47 μg pCE-K2N, 0.47 μg pCE-KdGl and 0.36 μg pCXB-EBNA1 were added to a 100 μl nucleofection solution, which was then used for transfection using the Nucleofector 2b.

The transfected fibroblasts were expanded on a 0.1% gelatin-coated tissue culture dish in M10 medium for 3−9 days following transfection. 10 ng/ml bFGF was also supplemented for transfected fibroblasts derived from adult marmosets and dog. After dissociation using Trypsin, the number of the fibroblasts and the transfection efficiency (calculated according to the EGFP fluorescence) were quantified using the Countess II FL (Themo Fisher). Cells were then transferred onto MEFs cultured on a 0.1% gelatin-coated tissue culture 6-well plate at a density of $1.0-10 \times 10^4$ cells per well. Twenty-four hours later, the medium was changed to 50% M10 medium and 50% NSM. After two days, the medium was changed to NSM. Medium change was performed every other day until colony picking. The induction medium NSM is composed of an 1:1 mixture of Neurobasal (Thermo Fisher) and DMEM/F-12 (Thermo Fisher), supplemented with 5% KSR, 1% N2 supplement (N2; Thermo Fisher), 2% B27 supplement (B27; Thermo Fisher), 1 mM L-glu, 1% NEAA, 0.1 mM 2-ME, 50 μg/ml AlbuMax I (Thermo Fisher), 10 ng/ml LIF, 3 μM CHIR99021 (CHIR; Axon Medchem), 1μM PD0325901 (Wako), 10μM Forskolin (Sigma) and 5 μM A83-01 (Santacruz). Primary colonies were mechanically isolated or dissociated in bulk, and transferred onto new MEFs for expansion using NSM supplemented with 10 μM Y-27632, which was removed two days after transferring. For primed conversion, early passage (P1-3) primary colony-forming cells (iNSLCs) that have been expanded were transferred onto new MEFs, and the medium was changed to ESM two days after transferring. After primed conversion for 3 weeks, iPSC colonies were mechanically picked and transferred onto new MEFs on a 24-well plate. For primed conversion of the iNSLCs derived from adult marmoset fibroblasts, 20 ng/ml Activin-A (ACTA; R&D Systems) was supplemented in the ESM during conversion. The iPSCs were cultured as described above for ESCs. Several iPSC lines were cultured in ESM supplemented with 10 ng/ml ACTA and 10 ng/ml TGF-β1 (Thermo Fisher) for enhancing cell growth.

Vector transfection into NSCs derived from embryonic marmosets was

performed using the NEPA21 Super Electroporator (Nepagene) as described previously for mouse NSCs (Tsuyama et al., 2015). In brief, marmoset NSCs were dissociated into single cells using TrypLE Select (Thermo Fischer). Electroporation was performed in the following condition: two poring pulses (270 V, 0.5 msec pulse length, 50 msec pulse interval and 10% decoy rate), with subsequent five transfer pulses (20V, 50 msec pulse length and interval, 40% decoy rate). We introduced a total of 11.7 μg DNA vectors into 1×10^6 NSCs. 1.5 μg pCE-hOCT3/4, 1.5 μg pCE-hSK, 1.5 μg pCE-hUL, 1.5 μg pCE-mp53DD, 1.5 μg pCXLE-EGFP, 1.5 μg pCE-K2N, 1.5 μg pCE-KdGl and 1.2 μg pCXB-EBNA1 were added to 100 μl of 1× Opti-MEM (Thermo Fisher), which was then used for electroporation. After transfection, the cells were maintained in a suspension culture using the MHM medium supplemented with 2% B27, 20 ng/ml bFGF, 20 ng/ml EGF, 10 ng/ml LIF and 1 μg/ml heparin sodium salt for 4 days. Then, the cells were transferred onto MEFs in the same medium. Two days later, the medium was changed to ESM.

Cell stocks of fibroblasts and iNSLCs were generated by slow-freezing using CELLBANKER1 (Zenoaq) at -80°C or -150°C. Cell stocks of ESCs and iPSCs were generated by vitrification using Cell Reservoir One for vitrification (Nacalai Tesque) in liquid N_2.

The derivation efficiency (%) was calculated as follows: (number of colonies / number of EGFP-positive transfected fibroblasts passaged onto MEFs) × 100.

Nomenclature

Marmoset ID (Table S1) and vector set (Fig. 1A) were used to name marmoset iNSLC (cjiNSLC) lines. For example, "cjiNSLC E01F A-2" corresponds to a marmoset iNSLC line which was derived from the fibroblasts of the E01F marmoset by using the EP-A vector set, while “cjiNSLC E02M B-0” corresponds to bulk iNSLCs derived from the fibroblasts of the E02M marmoset by using the EP-B vector set (without mechanical isolation of a colony).

Marmoset ID, vector set, and clone number of the parental iNSLC line were used to name marmoset iPSC (cjiPSC) lines. For example, "cjiPSC E01F A-2-2" corresponds to a marmoset iPSC line which was converted from the cjiNSLC E01F A-2. However, for the iPSC lines converted from bulk iNSLCs, "0" was used as the clone number of the parental iNSLC line. For example, "cjiPSC E02M B-0-7" was converted from bulk iNSLCs which were derived from E02M fibroblasts using the EP-B vector set.

Vector construction

Vectors used for reprogramming are summarized in Figure 1A. In the EP-B set, two novel episomal vectors, pCE-K2N and pCE-KdGl, were constructed using the expression-cassette-excised pCE-hUL as a backbone (pCE backbone). For constructing pCE-K2N, the *KLF2-F2A-NANOG* fragment was amplified by PCR from the pPB-C6F/TdTomato vector (Addgene #140826) (Kisa et al., 2017), and inserted into the pCE backbone. For constructing pCE-KdGl, the entire coding sequence of the marmoset *KDM4D* (single exon gene) was amplified from the genomic DNA, and fused to the human *GLIS1*, which was amplified from pCXLE-GLIS1 (kindly provided by Shinya Yamanaka) via a *Thoseaasigna* virus self-cleaving 2A peptide sequence (T2A), and inserted into the pCE backbone.

Vectors used for PGCLC experiments are shown in Figure S5A and S5D. Construction of the *BLIMP1-Venus* and *VASA-tdTomato* vectors (Figure S5A) were described previously (Yoshimatsu et al., 2019c). For construction of pPBCAG-rtTAM2-2A-SOX17GR-IH (Figure S5D, top), *T2A-SOX17-GR* was inserted into SmaI/NotI-digested pPBCAG-rtTAM2-IH (Addgene #140827) (Kisa et al., 2017) by Seamless cloning using GeneArt™ Seamless PLUS Cloning and Assembly Kit (Thermo Fisher). The *T2A-SOX17-GR* fragment was composed of *T2A* (a self-cleaving 2A peptide sequence from *Thosea asigna* virus capsid protein), human *SOX17* fused to the I747T-mutant ligand binding domain (500 – 777 aa) of the human *glucocorticoid receptor* (*GR*) gene (Brocard et al., 1998). pPB-tet-PH-PRDM1 (Figure S5D, bottom) contained the human *PRDM1* (*BLIMP1*) gene under the control of tetracycline-responsive promoter element with a Puromycin resistance cassette, which was kindly provided by Drs. Yuhki Nakatake and Minoru Ko (Keio University).

pCE-K2N, pCE-KdGl and pPBCAG-rtTAM2-2A-SOX17GR-IH have been deposited into Addgene (https://www.addgene.org; #154879, #154880 and #165079).

qPCR and RT-PCR

Extraction of total cellular RNA, reverse transcription and quantitative reverse transcription PCR (qPCR) analysis were performed as previously described (Kisa et al., 2017). For quantification of gene expression, we utilized a relative standard curve method. *GAPDH* was used as the internal control. All qPCR data was biologically and technically

triplicated. Expression level of each gene in No. 40 cjESCs was set to 1.0 in the qPCR analysis. Reverse transcription PCR (RT-PCR) analysis was performed using the TaKaRa Ex Taq (Takara) according to the manufacturer's introductions. In brief, 1× ExTaq Buffer, 0.4 mM each dNTP, 5 μM each primer, 0.5% ExTaq and 5% cDNA (reverse-transcribed from 4 ng/μl RNA) were diluted in nuclease-free water, and PCR was performed under the following condition: 95°C 20 sec, 30 repeats of 95°C 30 sec and 60°C 1 min, and then kept at 4°C until gel electrophoresis. Primers are listed in the next page.

Genomic PCR

For genomic PCR, cells were lysed overnight at 55°C in Cell Lysis Buffer consisting of 0.2 M Tris-HCl, 10 mM EDTA, 0.2% SDS and 0.2 M NaCl in nuclease-free water with 10 μg/ml Proteinase K. Genomic DNA was purified by a standard method using phenol-chloroform and ethanol, and subsequently diluted in TE buffer. PrimeSTAR Max DNA Polymerase (Takara) was used for genotyping PCR according to the manufacturer's introductions. In brief, a total 10 μl PCR solution consisted of 100 ng genomic DNA, 5 μl 2x PrimeSTAR Max DNA Polymerase, 1.6 μM each primer, 2% DMSO in nuclease-free water. PCR was performed under the following condition: 94°C 30 sec, 45 repeats of 98°C 10 sec and 68°C 1 min, followed by 68°C 10 min and then kept at 4°C until electrophoresis using 1% agarose gel. For the detection of residual episomal vectors, 0.5 ng of each episomal vector (pCE-hUL for the detection of OriP) was used as a positive control. Primers are listed below.

Gene*, reference	Usage	Sequence	Amplicon size
cj+hs+clf+ss *GAPDH* (Yoshimatsu et al., 2019b)	qPCR, RT-PCR	GCACCGTCAAGGCTGAGAAC	138 bp
		TGGTGAAGACGCCAGTGGA	
cj *OCT4*	qPCR	GCAAGCCCTCATTTCACCAG	77 bp
		CAAAATCCGAAGCCAGGTGTC	
	qPCR	GGAGGAAGCTGACAACAATGAAA	64 bp

cj+hs *OCT4* (Yoshimatsu et al., 2019b)		GGCCTGCACGAGGGTTT	
cj *NANOG* (Yoshimatsu et al., 2019b)	qPCR	ACGAACATGCCACCTGAAGA	107 bp
		TACGAGGAAGGGGAGGAGGT	
cj+hs *NANOG*	qPCR	GCCTGGAGCAGTCCCTTCTA	89 bp
		TCCAAGTCACTGGCAGGAGA	
cj *SOX2*	qPCR	ACAGTTGCAAACGTGGAGAGAAG	109 bp
		ACCACAGAGATGGTTTGCCAGTA	
cj *KLF4*	qPCR	CCCAGCTGAGTCAACTTGTGAG	155 bp
		ACCCCCTTGGCATTTTGTAAGT	
cj+hs *KLF4*	qPCR	AAGAGTTCCCATCTCAAGGCACA	91 bp
		GGGCGAATTTCCATCCACAG	
cj *ZFP42*	qPCR	CAAGCTCCCTTCTGGAATGTTCT	176 bp
		TTCTGCGAGCTGTTTAGGATCTG	
cj *DPPA5*	qPCR	ATCCAGAAGTGTTCCAGGTCCAG	286 bp
		CAGTTCATCCAAGGGCTCAGTT	
cj+hs *TERT*	qPCR	AGAGTGTCTGGAGCAAGTTGC	183 bp
		CGTAGTCCATGTTCACAATCG	
cj *LIN28*	qPCR	GCACAGGGAAAGCCAACA	216 bp
		GTGATGGTGTGAACCCCAAC	
cj+hs *LIN28* (Piskounova et al., 2011)	qPCR	AAGCGCAGATCAAAAGGAGA	113 bp
		CTGATGCTCTGGCAGAAGTG	
		CTTCGTGCCTACCCTTTTCAAGT	
clf *OCT4*	RT-PCR	CCCTCTGTGTCTGTCACCACTCT	184 bp
		TCTACACCCTTTGTGTTCCCAGA	
clf *NANOG*	RT-PCR	TTCCAGCAAAATTCTATGGGTGA	253 bp
		TAATGGGACACTATCGAGGCAGA	
clf *SOX2*	RT-PCR	ACAGTTGCAAACGTGGAAAAGAA	197 bp
		AACCTGTATGGCCATTTTTGCTT	
ss *OCT4*	RT-PCR	CGGAAGAGAAAGCGGACAAGTAT	199 bp

		CTCGTTGCGAATAGTCACTGCTT	
ss *NANOG*	RT-PCR	TCTTCACCAATGCCTGAGGTTTA	125 bp
		TGAATAAGCAGATCCATGGAGGA	
ss *SOX2*	RT-PCR	CCACCTACAGCATGTCCTACTCG	125 bp
		CCTGGAGTGGGAAGAAGAGGTAA	
		AACTTTCTGCAAAGCTCCTACCG	
cj+hs+clf *GAPDH* locus	Genomic PCR	GCACCGTCAAGGCTGAGAAC	138 bp
		TGGTGAAGACGCCAGTGGA	
cj+hs+clf *OCT4* locus	Genomic PCR	GGAGGAAGCTGACAACAATGAAA	341 bp
		GGCCTGCACGAGGGTTT	
ss *OCT4* locus	Genomic PCR	CGGAAGAGAAAGCGGACAAGTAT	298 bp
		CTCGTTGCGAATAGTCACTGCTT	
OriP (Yu et al., 2009)	Genomic PCR	TTCCACGAGGGTAGTGAACC	544 bp
		TCGGGGGTGTTAGAGACAAC	
cj+hs *SRY* locus	Genomic PCR	AACGTCCAGGATAGAGTGAAGCGA	240 bp
		CTTCCGACGAGGTCGATACTTATA	
OC: CAG-OCT4 (pCE-hOCT3/4)	Genomic PCR	TGCTAACCATGTTCATGCCTTCT	844 bp
		AAATTCTCCAGGTTGCCTCTCAC	
SK: SOX2-KLF4 (pCE-hSK)	Genomic PCR	ACTTCACATGTCCCAGCACTACC	469 bp
		ATCGTTGAACTCCTCGGTCTCTC	
UL: LMYC-Lin28 (pCE-hUL)	Genomic PCR	CAGCAGCAGTTGCAGAAAAGAAT	448 bp
		TAAAGGTGAACTCCACTGCCTCA	
MP: mp53DD (pCE-mp53DD)	Genomic PCR	CACAGTCGGATATCAGCCTCAAG	237 bp
		TAGACTGGCCCTTCTTGGTCTTC	
KN: CAG-KLF2 (pCE-K2N)	Genomic PCR	TGCTAACCATGTTCATGCCTTCT	1001 bp
		AGGGCTTCTCACCTGTGTGC	
KG: KDM4D-GLIS1 (pCE-KdGl)	Genomic PCR	GAAGTCGAGTTGCCTAGGAGAGC	851 bp
		CGGAGTCCATTTACACAGGTGAC	

*Primers specific for each species are indicated as cj, *Callithrix jacchus* (marmoset); hs, *Homo sapiens* (human); clf, *Canis lupus familiaris* (dog); ss, *Sus scrofa* (pig). All primers are designed not to amplify murine cDNA/genomic DNA sequences.

Three-germ-layer differentiation assay

For *in vivo* three-germ-layer differentiation assay, teratoma formation was performed as previously described (Tomioka et al., 2010). Teratomas were fixed with 4% PFA and subjected to Hematoxylin-Eosin staining or Hematoxilyn staining with immunocytochemical staining using an anti-Neurofilament 200kDa antibody (MAB5262; Merck).

For *in vitro* three-germ-layer differentiation assay, iPSCs were detached from MEFs *en bloc*, and transferred to a suspension culture for two weeks using M10 medium or EB medium (Yoshimatsu et al., 2019b). For further differentiation, the cells were plated onto poly-L-ornithine/fibronectin-coated glass coverslips for an additional two weeks in the same medium.

Direct neurosphere formation assay was performed as following: ESCs, iPSCs and iNSLCs were detached from MEFs and transferred to a suspension culture in MHM medium (Shimada et al., 2012) supplemented with 2% B27. After one week of suspension culture at which point primary neurospheres were formed, the cells were plated onto poly-L-ornithine/fibronectin-coated glass coverslips for an additional week of culture in the same medium.

For deriving gliogenic cells by the direct neurosphere formation assay, primary neurospheres were dissociated into single cells using TrypLE Select and transferred to a suspension culture in the same medium for an additional week to enable secondary neurosphere formation. Then, the cells were plated onto poly-L-ornithine/fibronectin-coated glass coverslips for one week of culture in the same medium. For posteriorization/ventralization of the iNSLCs, 1 μM retinoic acid (Tocris Bioscience), 2 μM Purmorphamine (Merck) and 3 μM CHIR were supplemented in the medium during secondary neurosphere formation, followed by an adherent culture in the same medium for one week.

Generation of PGC-reporter iPSCs, PGCLC induction and FACS

The generation of marmoset iPSCs (E01F A-2-2) harboring *BLIMP1-Venus* and *VASA-tdTomato* reporters was performed as described previously (Yoshimatsu et al., 2020; Yoshimatsu et al., 2019c). In brief, the iPSCs were transfected with 8 μg of pUC-DEST-cjBLIMP1-Venus-HygTK (Addgene #141028) and 2 μg of PX459 (Addgene #48139) containing single-guide RNA sequence for the marmoset PRDM1 gene

(GGAAAATCTTAAGGATCCAT) by lipofection, and subsequently selected in the presence of Hygromycin (25 µg/ml) for two weeks. Then, Hygromycin-resistant iPSC colonies were mechanically isolated and clonally expanded for genotyping PCR (Figure S5B, top). Next, the #8 (BV8) iPSC clone was transfected with 2 µg of pCMV-HyPBase (kindly provided by Dr. Kosuke Yusa, Sanger Institute) and 2 µg of pPB-VASA-tdTomato-pNeo, and subsequently selected in the presence of G418 (50 µg/ml) and Ganciclovir (1 µM). Then, resistant iPSC colonies were mechanically isolated and clonally expanded for PCR (Figure S5B, bottom). The #1 (BV8VT1) iPSC clone was used for further experiments.

For generation of marmoset ESCs and iPSCs with dexamethasone/doxycycline-inducible *SOX17/BLIMP1* transgenes, cells were transfected with 4 µg of pPBCAG-rtTAM2-2A-SOX17GR-IH, 4 µg of pPB-tet-PH-PRDM1 (Figure S5D) and 2 µg of pCMV-HyPBase (kindly provided by Dr. Kosuke Yusa) by lipofection as described previously (Yoshimatsu et al., 2019c), and subsequently selected in the presence of Puromycin (1 µg/ml) and Hygromycin (25 µg/ml) for two weeks.

The timetable of PGCLC induction is shown in Figure S5C. In brief, sub-confluent marmoset ESCs and iPSCs were dissociated using Trypsin, then plated onto a Poly-L-Ornithine (Sigma)-coated well without any dilution in Medium 1 (Day 0). The Medium 1 consisted of GK15 or αRB27 (GK15 for No. 40 ESCs and E01F A-2-2 iPSCs, αRB27 for DSY127 ESCs) (Sakai et al., 2020) supplemented with 100 ng/ml ACTA, 2 µg/ml Dexamethasone (Dex) and 10 µM Y-27632. The cells were cultured in the same medium until Day 4. Medium change was performed every other day. Supplementation of 3 µM CHIR was performed on Day 3.5. On Day 4, the cells were detached into single cells using TrypLE Select, then suspended in Medium 2, and 1×10^4 cells were aggregated in low attachment V-shaped 96-well plates (100 µl/well, Sumitomo Bakelite). The Medium 2 consisted of GK15 or αRB27 (GK15 for No. 40 ESCs and E01F A-2-2 iPSCs, αRB27 for DSY127 ESCs) supplemented with 500 ng/ml human recombinant BMP4, 100 ng/ml SCF, 50 ng/ml EGF, 10 ng/ml LIF, 2 µg/ml Dex, 2 µg/ml Doxycycline (Dox) and 10 µM Y-27632. Following single-cell dissociation of Day10-differentiated cells using Accutase (Nacalai Tesque), BLIMP1-Venus positive cells (detected by the FITC filter) were sorted using a Cell Sorter SH800Z (Sony Biotechnology). 7-AAD (Thermo Fisher) was used for the removal of dead cells (detected by APC filter).

Alkaline phosphatase staining and immunocytochemistry.

For alkaline phosphatase (AP) staining, cells were fixed with 100% ethanol for 10 min at room temperature (RT). AP staining was performed using the SIGMAFAST™ BCIP®/NBT (Sigma) according to the manufacturer's introductions.

For immunocytochemical analysis, cells were fixed with 4% paraformaldehyde (PFA) for 15-30 min at room temperature. After incubating with blocking buffer (PBS containing 0.05% Tween 20 (Sigma) and 10% goat or donkey serum) for 30−60 min at RT, the cells were incubated with primary antibodies at 4°C overnight. After incubation with primary antibodies, the cells were washed with PBS three times, and incubated with Alexa488-, Alexa555-, or Alexa647-conjugated secondary antibodies (Thermo Fisher) and 10 mg/ml Hoechst 33258 (Sigma) for one hour at RT. Primary antibodies used in this study were as follows: OCT4 (1:200; H134; Santa Cruz), NANOG (1:500; 1E6C4; Cell Signaling), SOX2 (1:200; AF1979; R&D Systems), SSEA3 (1:500, ab16286, Abcam), SSEA4 (1:500; MAB4304; Merck, TRA-1-60 (1:500; MAB4360; Merck), TRA-1-81 (1:500, MAB4381, Merck), βIII-tubulin (1:400; 2G10; Abcam), MAP2 (1:500; AB5392; Abcam), PAX6 (1:500; PD022, MBL), MSI1 (1:500; D270-3; MBL), αSMA (1:1000; 1A4; Sigma), SOX17 (1:200; AF1924; R&D Systems), HNF3β/FOXA2 (1:500; D5606; Cell Signaling) , ChAT (1:300, Aves Labs), Islet1 (1:300, 39.4D5, DSHB), Galactocerebroside (1:1000, MAB342, Merck), GFAP (1:300, BT-575, Biomedical Technologies), Doublecortin (1:200, AB5910, Merck).

Karyotyping. Q-banding and G-banding-based karyotyping analyses were performed by Chromosome Science Labo. Ltd. (http://www.chromoscience.jp)

3’IVT microarray analysis.

Extraction of total cellular RNA was performed as described above. Microarray analysis was performed using the GeneChip Marmoset Gene 1.0 ST Array (Thermo Fisher), which is specific for mRNAs (3’IVT; designed for exons in 3’ side) of marmoset genes (33,971 genes were analyzed using 656,668 probes in total), according to the manufacturer's introductions. The microarray data was analyzed using the GeneSpring software (Agilent).

Bulk mRNA-seq analysis.

Poly(A)+ RNA was selected and converted to a library of cDNA fragments (mean length: 350 bp) with adaptors attached to both ends for sequencing using the KAPA mRNA Capture Kit (KK8440; Kapa Biosystems), KAPA RNA HyperPrep Kit (KK8542; Kapa Biosystems), KAPA Pure Beads (KK8543; Kapa Biosystems) and SeqCap Adapter Kit A (Roche) according to the manufacturer's instructions. The cDNA libraries were quantified using the KAPA Library Quantification Kits (KK4828; Kapa Biosystems), and were sequenced using an Illumina HiSeqX to obtain 150-nucleotide sequences (paired-end). Data of mRNA-seq (*fastq* file format) were quality-checked, and low-quality reads (score < 30), adapter sequences, and overrepresented sequences such as poly-A chain were trimmed using the *Trim Galore!* (ver.0.4.0). The remaining reads were mapped to the *Homo sapiens* (hg19), *Callithrix jacchus* (cj3.2.1.86), *Canis lupus familiaris* (CanFam3.1) and *Sus scrofa* (Sscrofa11.1) genome using the *STAR* (ver.2.5.3a) (Dobin and Gingeras, 2015), and the output file (BAM file format) were summarized using the *featureCounts* (1.5.2) (Liao et al., 2014). The summarized data were processed by the *DESeq2* (3.3.0) (Love et al., 2014) for estimating their size factors, followed by the removal of reads not expressed in any of the samples. Subsequently, the data were normalized by varianceStabilizingTransformation (vst).

For mRNA-seq analysis, we also included deposited data of previous studies in GEO (https://www.ncbi.nlm.nih.gov/geo/) and DDBJ (https://www.ddbj.nig.ac.jp/) as following: marmoset ESCs (No40_ES_P63, DSY127_ES_P17, No20_ES_P45) and iPSCs (RNAiPS_1) in GSE152259 (Nakajima et al., 2019); marmoset ESCs (cjes001_P83, P91, P94) and iPSCs (DPZcj_iPSC1_P18, P19, P22, P24) in GSE64966 (Debowski et al., 2015); adult marmoset cortex in GSE152264 (Yoshimatsu et al., 2019a), marmoset early-stage embryos in GSE138944 (Shiozawa et al., 2020); porcine iPSCs and early-stage embryos: GSE92889 (Secher et al., 2017); human ESCs in ERA260913 (Chan et al., 2013).

We have deposited mRNA-seq data of the present study in The Gene Expression Omnibus database of NIH (https://www.ncbi.nlm.nih.gov/geo/), and the accession number is GSE152493.

Single-cell RamDA-seq analysis.

Single-cell RamDA cDNA library was prepared using GenNext® RamDA-seqTM Single Cell Kit (Toyobo) and Nextera XT DNA Library Preparation Kit (Illumina)

according to the manufacturer's introductions. Single cell sorting was performed using a Cell Sorter SH800Z (Sony Biotechnology). For sorting, FITC Mouse anti-Human TRA-1-60 Antigen (BD) for marmoset ESCs and iPSCs, and Alexa Fluor 647 Mouse anti-Human Alkaline Phosphatase (BD) for marmoset iNSLCs were used for the removal MEF contamination. In addition, 7-AAD (Thermo Fisher) was used for the removal of dead cells. Libraries were sequenced using an Illumina HiSeq 2000 to obtain 150-nucleotide sequences (paired-end). Sequenced reads were trimmed using *trimmomatic* based on the quality of reads and adaptor sequences. The remaining reads were mapped to the *Callithrix jacchus* (cj1700) genome using *STAR*, and gene expression level were counted using the (1.5.2). Normalization and downstream analysis were conducted using the *R* software.

Statistical analysis

All data were expressed as means ± S.E.M. The statistical significance of differences was analyzed by Student's *t*-test. Differences of $p < 0.05$ were showed as *, $p < 0.01$ were showed as ** and $p < 0.001$ were showed as ***, which were considered statistically significant.

Supplementary References

Brocard, J., Feil, R., Chambon, P., and Metzger, D. (1998). A chimeric Cre recombinase inducible by synthetic,but not by natural ligands of the glucocorticoid receptor. Nucleic Acids Res *26*, 4086-4090.

Campolo, F., Gori, M., Favaro, R., Nicolis, S., Pellegrini, M., Botti, F., Rossi, P., Jannini, E.A., and Dolci, S. (2013). Essential role of Sox2 for the establishment and maintenance of the germ cell line. Stem Cells *31*, 1408-1421.

Chan, Y.S., Goke, J., Ng, J.H., Lu, X., Gonzales, K.A., Tan, C.P., Tng, W.Q., Hong, Z.Z., Lim, Y.S., and Ng, H.H. (2013). Induction of a human pluripotent state with distinct regulatory circuitry that resembles preimplantation epiblast. Cell Stem Cell *13*, 663-675.

Chow, L., Johnson, V., Regan, D., Wheat, W., Webb, S., Koch, P., and Dow, S. (2017). Safety and immune regulatory properties of canine induced pluripotent stem cell-derived mesenchymal stem cells. Stem Cell Res *25*, 221-232.

Cong, X., Zhang, S.M., Ellis, M.W., and Luo, J. (2019). Large Animal Models for the Clinical Application of Human Induced Pluripotent Stem Cells. Stem Cells Dev *28*, 1288-

1298.

Debowski, K., Warthemann, R., Lentes, J., Salinas-Riester, G., Dressel, R., Langenstroth, D., Gromoll, J., Sasaki, E., and Behr, R. (2015). Non-viral generation of marmoset monkey iPS cells by a six-factor-in-one-vector approach. PLoS One *10*, e0118424.

Di Stefano, B., Prigione, A., and Broccoli, V. (2009). Efficient genetic reprogramming of unmodified somatic neural progenitors uncovers the essential requirement of Oct4 and Klf4. Stem Cells Dev *18*, 707-716.

Dobin, A., and Gingeras, T.R. (2015). Mapping RNA-seq Reads with STAR. Current protocols in bioinformatics *51*, 11.14.11-19.

Du, X., Feng, T., Yu, D., Wu, Y., Zou, H., Ma, S., Feng, C., Huang, Y., Ouyang, H., Hu, X.*, et al.* (2015). Barriers for Deriving Transgene-Free Pig iPS Cells with Episomal Vectors. Stem Cells *33*, 3228-3238.

Esteban, M.A., Xu, J., Yang, J., Peng, M., Qin, D., Li, W., Jiang, Z., Chen, J., Deng, K., Zhong, M.*, et al.* (2009). Generation of induced pluripotent stem cell lines from Tibetan miniature pig. J Biol Chem *284*, 17634-17640.

Ezashi, T., Telugu, B.P., Alexenko, A.P., Sachdev, S., Sinha, S., and Roberts, R.M. (2009). Derivation of induced pluripotent stem cells from pig somatic cells. Proc Natl Acad Sci U S A *106*, 10993-10998.

Gao, X., Nowak-Imialek, M., Chen, X., Chen, D., Herrmann, D., Ruan, D., Chen, A.C.H., Eckersley-Maslin, M.A., Ahmad, S., Lee, Y.L.*, et al.* (2019). Establishment of porcine and human expanded potential stem cells. Nat Cell Biol *21*, 687-699.

Hatoya, S., Torii, R., Kondo, Y., Okuno, T., Kobayashi, K., Wijewardana, V., Kawate, N., Tamada, H., Sawada, T., Kumagai, D.*, et al.* (2006). Isolation and characterization of embryonic stem-like cells from canine blastocysts. Mol Reprod Dev *73*, 298-305.

Hou, P., Li, Y., Zhang, X., Liu, C., Guan, J., Li, H., Zhao, T., Ye, J., Yang, W., Liu, K.*, et al.* (2013). Pluripotent stem cells induced from mouse somatic cells by small-molecule compounds. Science *341*, 651-654.

Irie, N., Weinberger, L., Tang, W.W., Kobayashi, T., Viukov, S., Manor, Y.S., Dietmann, S., Hanna, J.H., and Surani, M.A. (2015). SOX17 is a critical specifier of human primordial germ cell fate. Cell *160*, 253-268.

Kim, J., Efe, J.A., Zhu, S., Talantova, M., Yuan, X., Wang, S., Lipton, S.A., Zhang, K., and Ding, S. (2011). Direct reprogramming of mouse fibroblasts to neural progenitors. Proc Natl Acad Sci U S A *108*, 7838-7843.

Kim, J.B., Greber, B., Arauzo-Bravo, M.J., Meyer, J., Park, K.I., Zaehres, H., and Scholer, H.R. (2009a). Direct reprogramming of human neural stem cells by OCT4. Nature *461*, 649-643.

Kim, J.B., Sebastiano, V., Wu, G., Arauzo-Bravo, M.J., Sasse, P., Gentile, L., Ko, K., Ruau, D., Ehrich, M., van den Boom, D.*, et al.* (2009b). Oct4-induced pluripotency in adult neural stem cells. Cell *136*, 411-419.

Kim, J.B., Zaehres, H., Wu, G., Gentile, L., Ko, K., Sebastiano, V., Arauzo-Bravo, M.J., Ruau, D., Han, D.W., Zenke, M.*, et al.* (2008). Pluripotent stem cells induced from adult neural stem cells by reprogramming with two factors. Nature *454*, 646-650.

Kisa, F., Shiozawa, S., Oda, K., Yoshimatsu, S., Nakamura, M., Koya, I., Kawai, K., Suzuki, S., and Okano, H. (2017). Naive-like ESRRB(+) iPSCs with the Capacity for Rapid Neural Differentiation. Stem Cell Reports *9*, 1825-1838.

Kobayashi, T., Zhang, H., Tang, W.W.C., Irie, N., Withey, S., Klisch, D., Sybirna, A., Dietmann, S., Contreras, D.A., Webb, R.*, et al.* (2017). Principles of early human development and germ cell program from conserved model systems. Nature *546*, 416-420.

Lee, A.S., Xu, D., Plews, J.R., Nguyen, P.K., Nag, D., Lyons, J.K., Han, L., Hu, S., Lan, F., Liu, J.*, et al.* (2011). Preclinical derivation and imaging of autologously transplanted canine induced pluripotent stem cells. J Biol Chem *286*, 32697-32704.

Liao, Y., Smyth, G.K., and Shi, W. (2014). featureCounts: an efficient general purpose program for assigning sequence reads to genomic features. Bioinformatics (Oxford, England) *30*, 923-930.

Love, M.I., Huber, W., and Anders, S. (2014). Moderated estimation of fold change and dispersion for RNA-seq data with DESeq2. Genome biology *15*, 550.

Lu, J., Liu, H., Huang, C.T., Chen, H., Du, Z., Liu, Y., Sherafat, M.A., and Zhang, S.C. (2013). Generation of integration-free and region-specific neural progenitors from primate fibroblasts. Cell Rep *3*, 1580-1591.

Luo, J., Suhr, S.T., Chang, E.A., Wang, K., Ross, P.J., Nelson, L.L., Venta, P.J., Knott, J.G., and Cibelli, J.B. (2011). Generation of leukemia inhibitory factor and basic fibroblast growth factor-dependent induced pluripotent stem cells from canine adult somatic cells. Stem Cells Dev *20*, 1669-1678.

Nakajima, M., Yoshimatsu, S., Sato, T., Nakamura, M., Okahara, J., Sasaki, E., Shiozawa, S., and Okano, H. (2019). Establishment of induced pluripotent stem cells from common

marmoset fibroblasts by RNA-based reprogramming. Biochem Biophys Res Commun *515*, 593-599.
Nakajima-Koyama, M., Lee, J., Ohta, S., Yamamoto, T., and Nishida, E. (2015). Induction of Pluripotency in Astrocytes through a Neural Stem Cell-like State. J Biol Chem *290*, 31173-31188.
Nii, T., Marumoto, T., Kawano, H., Yamaguchi, S., Liao, J., Okada, M., Sasaki, E., Miura, Y., and Tani, K. (2014). Analysis of essential pathways for self-renewal in common marmoset embryonic stem cells. FEBS Open Bio *4*, 213-219.
Nishimura, T., Hatoya, S., Kanegi, R., Wijesekera, D.P.H., Sanno, K., Tanaka, E., Sugiura, K., Hiromitsu Tamada, N.K., Imai, H., and Inaba, T. (2017). Feeder-independent canine induced pluripotent stem cells maintained under serum-free conditions. Mol Reprod Dev *84*, 329-339.
Ogorevc, J., Orehek, S., and Dovc, P. (2016). Cellular reprogramming in farm animals: an overview of iPSC generation in the mammalian farm animal species. J Anim Sci Biotechnol *7*, 10.
Ohinata, Y., Ohta, H., Shigeta, M., Yamanaka, K., Wakayama, T., and Saitou, M. (2009). A signaling principle for the specification of the germ cell lineage in mice. Cell *137*, 571-584.
Piskounova, E., Polytarchou, C., Thornton, J.E., LaPierre, R.J., Pothoulakis, C., Hagan, J.P., Iliopoulos, D., and Gregory, R.I. (2011). Lin28A and Lin28B inhibit let-7 microRNA biogenesis by distinct mechanisms. Cell *147*, 1066-1079.
Sakai, Y., Nakamura, T., Okamoto, I., Gyobu-Motani, S., Ohta, H., Yabuta, Y., Tsukiyama, T., Iwatani, C., Tsuchiya, H., Ema, M.*, et al.* (2020). Induction of the germ cell fate from pluripotent stem cells in cynomolgus monkeysdagger. Biol Reprod *102*, 620-638.
Sasaki, E., Hanazawa, K., Kurita, R., Akatsuka, A., Yoshizaki, T., Ishii, H., Tanioka, Y., Ohnishi, Y., Suemizu, H., Sugawara, A.*, et al.* (2005). Establishment of novel embryonic stem cell lines derived from the common marmoset (Callithrix jacchus). Stem Cells *23*, 1304-1313.
Sasaki, K., Nakamura, T., Okamoto, I., Yabuta, Y., Iwatani, C., Tsuchiya, H., Seita, Y., Nakamura, S., Shiraki, N., Takakuwa, T.*, et al.* (2016). The Germ Cell Fate of Cynomolgus Monkeys Is Specified in the Nascent Amnion. Dev Cell *39*, 169-185.
Sasaki, K., Yokobayashi, S., Nakamura, T., Okamoto, I., Yabuta, Y., Kurimoto, K., Ohta, H., Moritoki, Y., Iwatani, C., Tsuchiya, H.*, et al.* (2015). Robust In Vitro Induction of

Human Germ Cell Fate from Pluripotent Stem Cells. Cell Stem Cell *17*, 178-194.

Schneider, M.R., Adler, H., Braun, J., Kienzle, B., Wolf, E., and Kolb, H.J. (2007). Canine embryo-derived stem cells--toward clinically relevant animal models for evaluating efficacy and safety of cell therapies. Stem Cells *25*, 1850-1851.

Secher, J.O., Ceylan, A., Mazzoni, G., Mashayekhi, K., Li, T., Muenthaisong, S., Nielsen, T.T., Li, D., Li, S., Petkov, S.*, et al.* (2017). Systematic in vitro and in vivo characterization of Leukemia-inhibiting factor- and Fibroblast growth factor-derived porcine induced pluripotent stem cells. Mol Reprod Dev *84*, 229-245.

Shimada, H., Nakada, A., Hashimoto, Y., Shigeno, K., Shionoya, Y., and Nakamura, T. (2010). Generation of canine induced pluripotent stem cells by retroviral transduction and chemical inhibitors. Mol Reprod Dev *77*, 2.

Shimada, H., Okada, Y., Ibata, K., Ebise, H., Ota, S., Tomioka, I., Nomura, T., Maeda, T., Kohda, K., Yuzaki, M.*, et al.* (2012). Efficient derivation of multipotent neural stem/progenitor cells from non-human primate embryonic stem cells. PLoS One *7*, e49469.

Shiozawa, S., Nakajima, M., Okahara, J., Kuortaki, Y., Kisa, F., Yoshimatsu, S., Nakamura, M., Koya, I., Yoshimura, M., Sasagawa, Y.*, et al.* (2020). Primed to naive-like conversion of the common marmoset embryonic stem cells. Stem Cells Dev.

Takahashi, K., Tanabe, K., Ohnuki, M., Narita, M., Sasaki, A., Yamamoto, M., Nakamura, M., Sutou, K., Osafune, K., and Yamanaka, S. (2014). Induction of pluripotency in human somatic cells via a transient state resembling primitive streak-like mesendoderm. Nat Commun *5*, 3678.

Tomioka, I., Maeda, T., Shimada, H., Kawai, K., Okada, Y., Igarashi, H., Oiwa, R., Iwasaki, T., Aoki, M., Kimura, T.*, et al.* (2010). Generating induced pluripotent stem cells from common marmoset (Callithrix jacchus) fetal liver cells using defined factors, including Lin28. Genes Cells *15*, 959-969.

Tsukamoto, M., Nishimura, T., Yodoe, K., Kanegi, R., Tsujimoto, Y., Alam, M.E., Kuramochi, M., Kuwamura, M., Ohtaka, M., Nishimura, K.*, et al.* (2018). Generation of Footprint-Free Canine Induced Pluripotent Stem Cells Using Auto-Erasable Sendai Virus Vector. Stem Cells Dev *27*, 1577-1586.

Tsuyama, J., Bunt, J., Richards, L.J., Iwanari, H., Mochizuki, Y., Hamakubo, T., Shimazaki, T., and Okano, H. (2015). MicroRNA-153 Regulates the Acquisition of Gliogenic Competence by Neural Stem Cells. Stem Cell Reports *5*, 365-377.

Wang, L., Wang, L., Huang, W., Su, H., Xue, Y., Su, Z., Liao, B., Wang, H., Bao, X., Qin, D., *et al.* (2013). Generation of integration-free neural progenitor cells from cells in human urine. Nat Methods *10*, 84-89.

West, F.D., Uhl, E.W., Liu, Y., Stowe, H., Lu, Y., Yu, P., Gallegos-Cardenas, A., Pratt, S.L., and Stice, S.L. (2011). Brief report: chimeric pigs produced from induced pluripotent stem cells demonstrate germline transmission and no evidence of tumor formation in young pigs. Stem Cells *29*, 1640-1643.

Wu, Z., Chen, J., Ren, J., Bao, L., Liao, J., Cui, C., Rao, L., Li, H., Gu, Y., Dai, H., *et al.* (2009). Generation of pig induced pluripotent stem cells with a drug-inducible system. J Mol Cell Biol *1*, 46-54.

Yoshimatsu, S., Nakamura, M., Nakajima, M., Nemoto, A., Sato, T., Sasaki, E., Shiozawa, S., and Okano, H. (2019a). Evaluating the efficacy of small molecules for neural differentiation of common marmoset ESCs and iPSCs. Neurosci Res.

Yoshimatsu, S., Okahara, J., Sone, T., Takeda, Y., Nakamura, M., Sasaki, E., Kishi, N., Shiozawa, S., and Okano, H. (2019b). Robust and efficient knock-in in embryonic stem cells and early-stage embryos of the common marmoset using the CRISPR-Cas9 system. Sci Rep *9*, 1528.

Yoshimatsu, S., Sato, T., Yamamoto, M., Sasaki, E., Nakajima, M., Nakamura, M., Shiozawa, S., Noce, T., and Okano, H. (2020). Generation of a male common marmoset embryonic stem cell line DSY127-BV8VT1 carrying double reporters specific for the germ cell linage using the CRISPR-Cas9 and PiggyBac transposase systems. Stem Cell Res *44*, 101740.

Yoshimatsu, S., Sone, T., Nakajima, M., Sato, T., Okochi, R., Ishikawa, M., Nakamura, M., Sasaki, E., Shiozawa, S., and Okano, H. (2019c). A versatile toolbox for knock-in gene targeting based on the Multisite Gateway technology. PLoS One *14*, e0221164.

Yu, J., Hu, K., Smuga-Otto, K., Tian, S., Stewart, R., Slukvin, II, and Thomson, J.A. (2009). Human induced pluripotent stem cells free of vector and transgene sequences. Science *324*, 797-801.
